# A Blueprint for the Stabilization of Sub‐Valent Alkaline Earth Complexes**

**DOI:** 10.1002/chem.202301850

**Published:** 2023-08-14

**Authors:** Alex W. J. Bowles, Yu Liu, Matthew P. Stevens, Iñigo J. Vitorica‐Yrezabal, Claire L. McMullin, Fabrizio Ortu

**Affiliations:** ^1^ School of Chemistry University of Leicester University Road Leicester LE1 7RH UK; ^2^ Department of Chemistry The University of Manchester Oxford Road Manchester M13 9PL UK; ^3^ Department of Chemistry University of Bath Claverton Down Bath BA2 7AY UK

**Keywords:** alkaline earth, barium, calcium, strontium, sub-valent

## Abstract

The study of sub‐valent Group 2 chemistry is a relatively new research field, being established in 2007 with the report of the first Mg(I) dimers. These species are stabilized by the formation of a Mg−Mg covalent bond; however, the extension of this chemistry to heavier alkaline earth (AE) metals has been frustrated by significant synthetic challenges, primarily associated with the instability of heavy AE−AE interactions. Here we present a new blueprint for the stabilization of heavy AE(I) complexes, based upon the reduction of AE(II) precursors with planar coordination geometries. We report the synthesis and structural characterisation of homoleptic trigonal planar AE(II) complexes of the monodentate amides {N(SiMe_3_)_2_}^−^ and {N(Mes)(SiMe_3_)}^−^. DFT calculations showed that the LUMOs of these complexes all show some *d‐*character for AE = Ca−Ba. DFT analysis of the square planar Sr(II) complex [Sr{N(SiMe_3_)_2_}(dioxane)_2_]_∞_ revealed analogous frontier orbital *d*‐character. AE(I) complexes that could be accessed by reduction of these AE(II) precursors were modelled computationally, revealing exergonic formation in all cases. Crucially, NBO calculations show that some *d‐*character is preserved in the SOMO of theoretical AE(I) products upon reduction, showing that *d*‐orbitals could play a crucial role in achieving stable heavy AE(I) complexes.

## Graphical abstract

Group 2 metals are classically found in the +2 oxidation state and their sub‐valent chemistry is hugely underdeveloped. Here we present a blueprint for a new approach towards the synthesis and stabilization of heavy Group 2 metals in the +1 oxidation state, based on the use of highly equatorial ligand environments (i. e. trigonal planar, square planar). Our computational analysis highlights the potential role played by d‐orbitals to access these species.

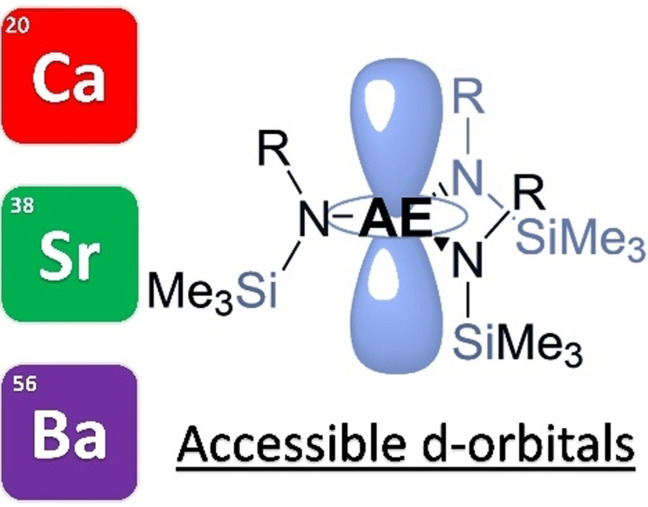

## Introduction

**Figure 1:**
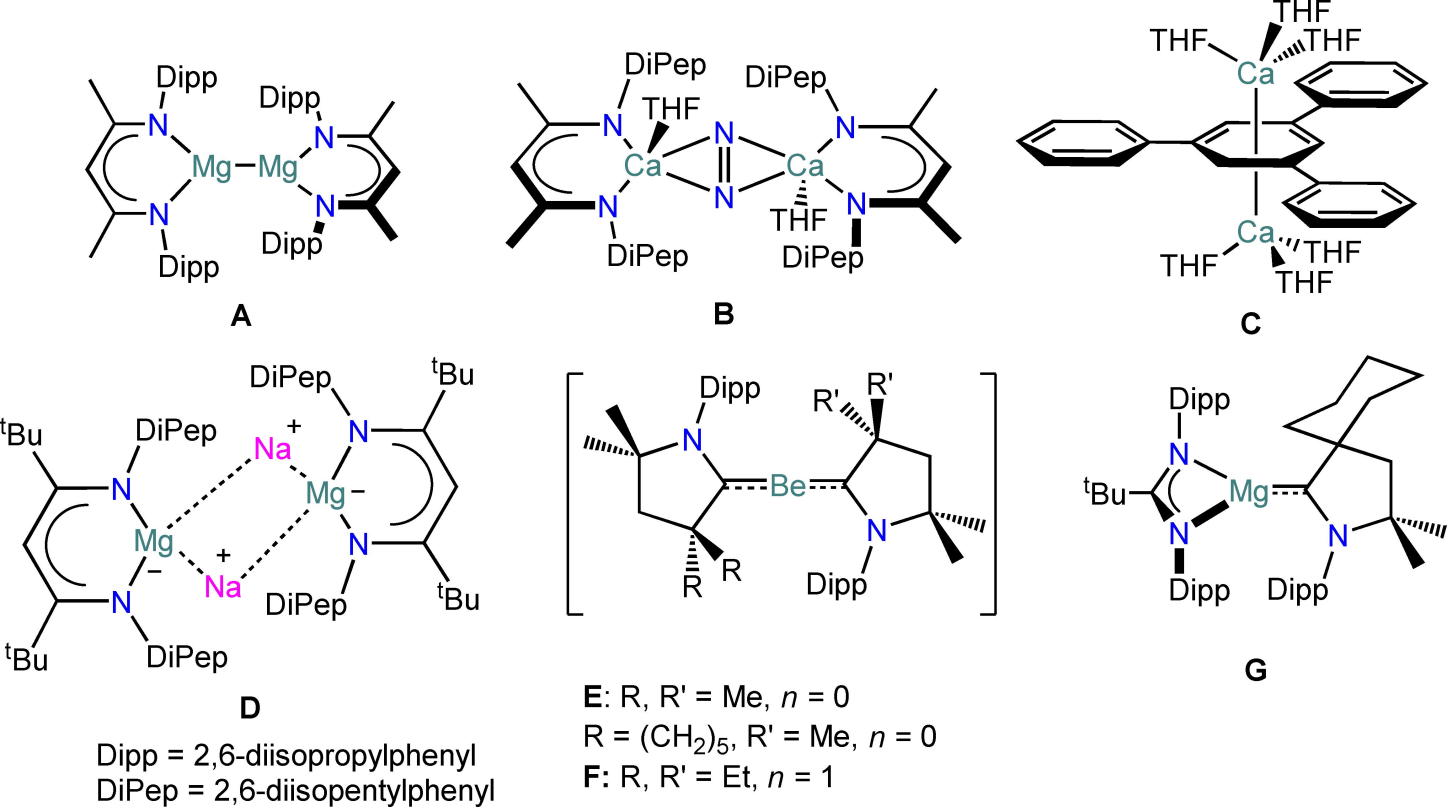
Landmark examples in sub‐valent AE chemistry.[^2^, ^13^, ^14^, ^16^, ^17^, ^18^, ^19^]

The chemistry of the alkaline earth (AE=Mg‐Ba) elements is classically dominated by the +2 oxidation state, due to the stability of AE(II) cations. This arises from their closed‐shell configuration, and is further demonstrated by their large reduction potentials: (AE^+2/0^: Mg=−2.37 V; Ca=−2.87 V; Sr=−2.90 V; Ba=−2.91 V).^[1]^ Consequently, classical AE(II) cations are considered redox‐inert, thus posing a significant obstacle for catalytic applications where reversible oxidative and reductive steps are required. Molecular sub‐valent AE compounds were first reported by Jones and co‐workers in 2007 with the landmark isolation of Mg(I) dimers (e.g. [{Mg(BDI^Dipp^)}_2_] (**A**); BDI^Dipp^={[(Dipp)NC(Me)]_2_CH}^−^; Dipp=2,6‐diisopropylphenyl) (Figure 1).^[2]^ Since this initial discovery, Mg(I) dimers have been used as bespoke reducing agents towards an array of organic and inorganic substrates.[^3^, ^4^, ^5^, ^6^, ^7^] Mg(I) dimers are usually obtained via reduction of heteroleptic Mg(II) halide precursors with alkali metals.[^8^, ^9^] However, extension of this methodology to isolate the heavier congeners (i. e. Ca−Ba) has not been successful, largely due to the instability of AE−AE interactions.[^10^, ^11^, ^12^] The attempted synthesis of a Ca(I) analogue of **A** by the alkali metal reduction of [{Ca(^tBu^BDI^DiPep^)(μ‐I)}_2_] (^tBu^BDI^DiPep^={[(DiPep)NC(^t^Bu)]_2_CH}^−^; DiPep=2,6‐diisopentylphenyl) by Harder and co‐workers gave the di‐reduced benzene complex [{Ca(BDI^DiPep^)}_2_(μ‐C_6_H_6_)] and the dinitrogen activation product [{Ca(BDI^DiPep^)(THF)}_2_(μ‐N_2_)] (**B**), which are proposed to form via a transient Ca(I) species.^[13]^ Harder and co‐workers have also shown that reduction of [{Ca(BDI^DiPep^)(μ‐I)}_2_] using mechanochemical methods affords a highly reactive deep purple material that the authors proposed to be the radical Ca(I) complex [Ca(BDI^DiPep^)(THF)].^[7]^ Despite continued efforts, structurally authenticated formally sub‐valent calcium complexes remain limited to the inverse‐sandwich complex [{Ca(THF)_3_}_2_(tpb)] (**C**; tpb=1,3,5‐triphenylbenzene) reported by Westerhausen and co‐workers, though the oxidation state of Ca in this complex remains unclear.[^14^, ^15^]

Monovalent AE radicals, AE(R) (R=methyl, cyclopentadienyl) have only been obtained as transient species in the gas phase.[^11^, ^16^, ^17^, ^18^, ^19^] Harder and co‐workers proposed that super‐bulky ligand systems could cause an elongation of the Mg−Mg interaction, that would in turn lead to the stabilization of molecular Mg(I) radicals.[^20^, ^21^] Rösch et al. reduced the Mg(II) precursor [Mg(^tBu^BDI^DiPep^)(I)] with Na to give the Mg(0) dimer [{Mg(^tBu^BDI^DiPep^)Na}_2_] (**D**).^[22]^ Furthermore, Braunschweig^[23]^ and Gilliard^[24]^ reported Be(0) and Be(I) species, [Be(CAAC)_2_] (**E**; CAAC=cyclic (alkyl)(amino)carbene) and [Be(CAAC)_2_][X] (**F**; X=[Be(TEMPO)_3_]^−^, [B(C_6_F_5_)_4_]^−^; TEMPO=2,2,6,6‐tetramethylpiperidinyloxyl). More recently, Jędrzkiewicz et al. reported the isolation of a monomeric formal Mg(I) radical [Mg{^t^BuC (NDipp)_2_}(CAAC)] (**G**).^[25]^ Compounds **E**‐**G** all display a high degree of charge delocalisation from the metal centre to the π‐system of the ligand.[^26^, ^27^] Furthermore, transient monomeric AE radicals have been reported by Harder^[28]^ and Jones,^[29]^ via mechanochemical and photochemical methods respectively.

**Scheme 1:**
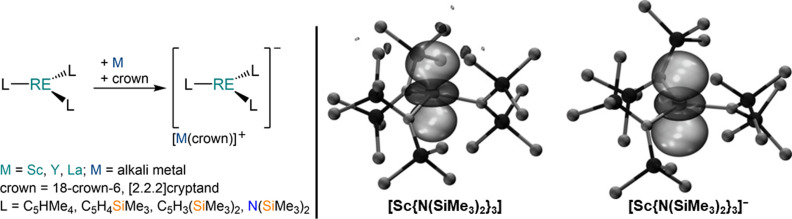
Synthesis of RE(II) *tris*‐amido and *tris*‐cyclopentadienyl complexes by Lappert and Evans (left),[^32^, ^33^, ^34^, ^35^, ^36^, ^37^, ^39^, ^40^, ^41^] and frontier orbitals (right) of [Sc{N(SiMe_3_)_2_}_3_] (LUMO) and [Sc{N(SiMe_3_)_2_}_3_]^−^ (SOMO) (reproduced with permission from *Angew. Chem. Int. Ed*., Ref. [39]).^[39]^

The instability of AE radical intermediates and AE−AE bonds for the heavy metals, as well as charge delocalisation, all currently frustrate efforts to isolate monovalent heavy AE radical complexes,[^30^, ^31^] thus an alternative approach may be needed to open up heavy AE(I) chemistry more rapidly. Lappert and Evans have demonstrated that *tris*‐cyclopentadienyl rare earth (RE) complexes [RE(Cp^R^)_3_] (RE=Y, La Cp^R^=C_5_H_4_SiMe_3_, Cp′; C_5_H_3_(SiMe_3_)_2_‐1,3, Cp′′) can be reduced with alkali metal reagents in the presence of a sequestering agent, usually 18‐crown‐6 (18c6) or [2.2.2]cryptand (crypt), to give the RE(II) complexes [M(L)][RE(Cp^R^)] (RE=Y, Cp^R^=Cp′, M=Li−Cs, L=crypt, 18c6;[^32^, ^33^, ^34^, ^35^] RE=La, Cp^R^=Cp′, Cp′′, C_5_HMe_4_, M=Li, K, L=18c6, crypt, THF) (Scheme 1).[^32^, ^36^, ^37^, ^38^] Evans and co‐workers have extended this approach to RE(II) *tris*‐amide complexes stabilized with the ubiquitous {N(SiMe_3_)_2_}^−^ ligand, leading to the isolation of [M(crown)][Sc{N(SiMe_3_)_2_}_3_] (M=K, Cs, crown=18c6, crypt) and [K(18c6)_2_][Y{N(SiMe_3_)_2_}_3_].[^39^, ^40^, ^41^] Frontier molecular orbital analysis of the RE^3+^ precursors revealed a LUMO with strong *d*_z2_ character, which is preserved in the SOMO of the RE^2+^ anions, thus giving a formal *nd* ^1^ configuration to all these species. This is particularly important for the La^2+^ derivative, where the equatorial ligand environment promotes the lowering in energy of the *d*‐manifold to disfavour a 4*f* ^1^3*d* ^0^ configuration.^[42]^

We reasoned that AE^2+^ (Ca−Ba) complexes with trigonal planar geometries could have a similar frontier orbital profile to the RE^3+^ congeners (Sc, Y, La), thus making them suitable for reductive chemistry. It is noteworthy that the role of *d*‐orbitals in heavy AE complexes has been intensely investigated in recent years and spectroscopic and theoretical evidence of *d*‐orbital involvement in AE(CO)_8_ (AE=Ca−Ba) complexes in low‐temperature neon matrices has been established.[^13^, ^43^, ^44^, ^45^, ^46^] Here we report the synthesis of a series of *tris*‐amide AE(II) complexes using the {N(SiMe_3_)_2_}^−^ and {N(Mes)(SiMe_3_)}^−^ (Mes=2,4,6‐trimethylphenyl) ligand sets, in order to analyse their frontier molecular orbitals and assess their suitability for reduction to heavy AE(I) complexes via *d*‐orbital occupation. Numerous trigonal planar M(II) *tris*‐amide AE(II) complexes have been reported previously,[^47^, ^48^, ^49^, ^50^, ^51^, ^52^, ^53^, ^54^, ^55^] however to the best of our knowledge the feasibility of their reductive chemistry has not yet been assessed computationally.

## Results

### Synthesis and structural characterization

**Scheme 2:**
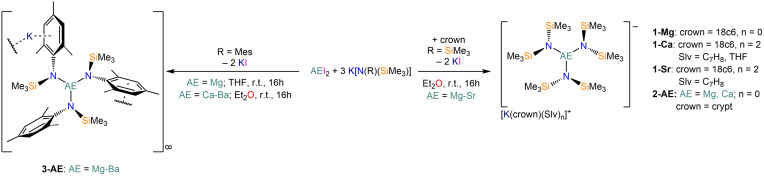
Synthesis of AE(II) *tris‐*amide complexes via salt metathesis, in the presence of crown ethers, 18‐crown‐6 (**1**‐**AE**) or [2.2.2]cryptand (**2**‐**AE**) and without sequestering agents (**3**‐**AE**).

The separated ion‐pair AE(II) complexes [K(18c6)(Slv)_n_][AE{N(SiMe_3_)_2_}_3_] (**1**‐**AE**: AE=Mg, n=0; AE=Ca, crown=18c6, n=2, Slv=THF, C_7_H_8_) and [K(crypt)][AE{N(SiMe_3_)_2_}_3_] (**2**‐**AE**: AE=Mg, Ca) were obtained via salt metathesis reactions between AEI_2_ and three equivalents of K[N(SiMe_3_)_2_], followed by reaction with either 18‐crown‐6 or [2.2.2]cryptand (Scheme 2). This methodology works very efficiently for Mg−Sr, but it could not be transferred to Ba. Salt metathesis reactions between K[N(Mes)(SiMe_3_)] and AEI_2_ afforded the AE(II) *tris*‐amide complexes [AE{N(Mes)(SiMe_3_)}_3_K]_∞_ (**3**‐**AE**: AE=Mg−Ba) in excellent yields (Scheme 2); a sequestering agent was not added to these complexes as the K^+^ cations were coordinated by the aryl rings of the silylamide ligand. Due to solubility issues, THF had to be used in the purification of **3**‐**Ba**, yielding the solvent adduct, **3**‐**Ba⋅THF**. Attempts to transfer these methods to the more sterically demanding silylaryl/amide ligand {N(Dipp)(SiMe_3_)}^−^ (Dipp=2,6‐diisopropylphenyl) yielded exclusively solvated AE(II) *bis*‐amide complexes in all cases.[^56^, ^57^]

**Figure 2:**
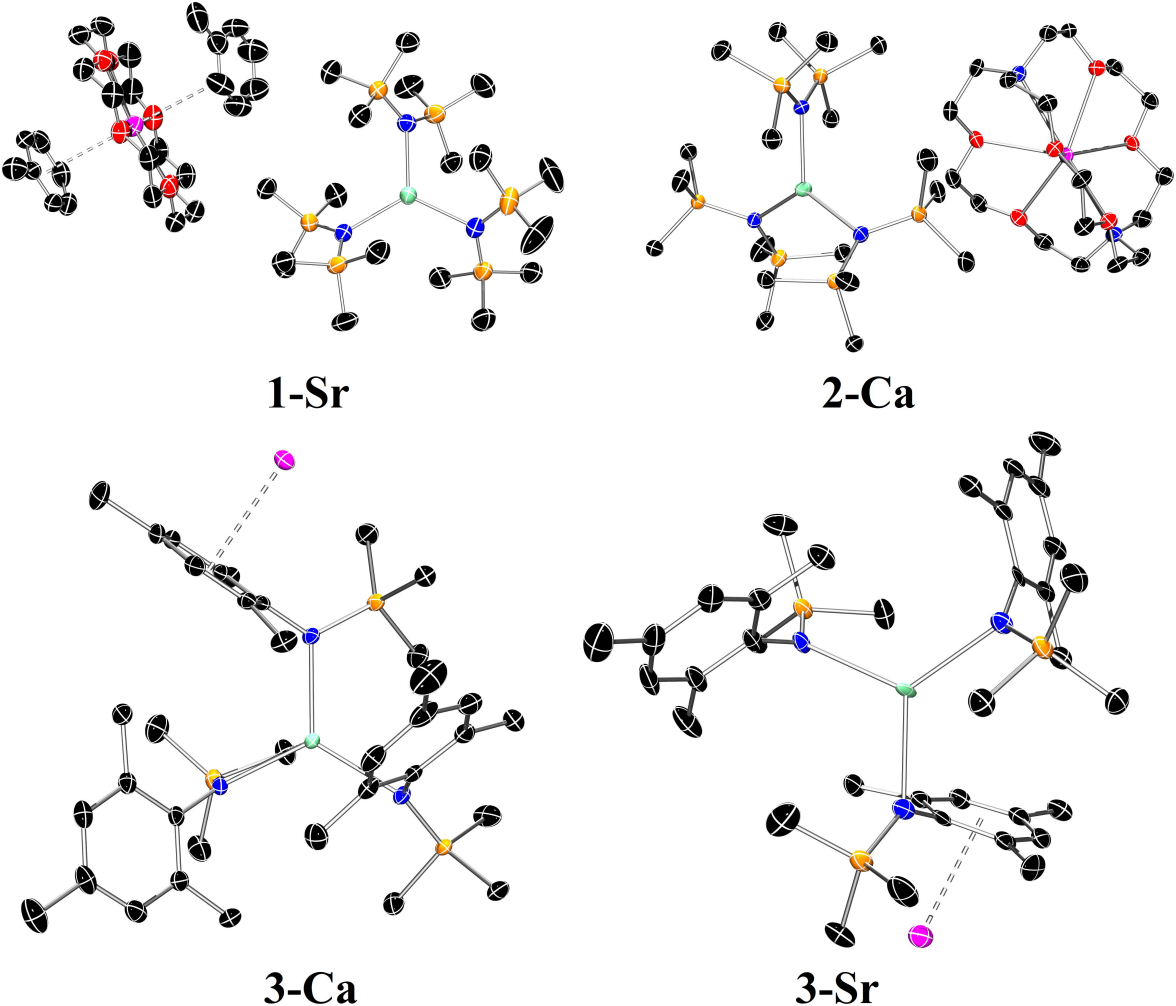
Crystal structures of **1**‐**Sr** (top left), **2**‐**Ca** (top right), **3**‐**Ca** and **3**‐**Sr** (bottom row). Ellipsoids are set at 50 % probability level. Hydrogen atoms have been omitted for clarity. Legend: carbon (black), nitrogen (blue), oxygen (red), silicon (orange), potassium (magenta), AE metal (aquamarine).

All compounds **1**–**3**‐**AE** were fully characterized via multinuclear NMR spectroscopy, IR spectroscopy, elemental analyses and single crystal X‐ray diffraction. Structural authentication of the AE(II) *tris*‐amide complexes reveals the desired 3‐coordinate arrangement about the AE centre (Figure 2 and Figure S38‐40, Tables S4 and S5), with the only exception a 4‐coordinate complex **3**‐**Ba⋅THF** (Figure S42). The [AE{N(SiMe_3_)_2_}_3_]^−^ anions in **1**‐**AE** and **2**‐**AE** display near‐perfect trigonal planar geometries, with the metal centres in close proximity to the N^N^N planes and N−AE−N bond angles close to the ideal value of 120° (Table S4). The [AE{N(Mes)(SiMe_3_)}_3_K] monomers of **3**‐**AE** are also close to ideal trigonal planar geometries, with the potassium cation encapsulated by mesityl substituents to give polymeric arrays (Table S5). The orientation of the mesityl substituents varies in each structure, largely influenced by the presence of the potassium cations intercalated within each coordination polymer. For **3**‐**Mg**, two of the mesityl substituents point in the same direction with respect to the N^N^N plane and encapsulate a potassium cation, whilst the third ring points below the plane and interacts with a potassium cation from a neighbouring unit. In the case of **3**‐**Ca** all three mesityl groups are positioned on the same face of the molecule. Finally, in **3**‐**Sr** the orientation of the aryl substituents is analogous to that observed for **3**‐**Mg**, though each potassium cation is encapsulated by three mesityl rings each belonging to different units. The Ca−N bond distances in [Ca{N(SiMe_3_)_2_}_3_]^−^ anions [**1**‐**Ca**: 2.3103(10)‐2.3121(10) Å;^[51]^ **2**‐**Ca**: 2.309(3)‐2.318(2) Å] are comparable to those observed in **3**‐**Ca**, with the exception of one elongated metal‐ligand interaction [2.390(11) Å]. This could be due to the potassium cation encapsulated between aryl ligands in **3**‐**Ca**, since steric properties of {N(Mes)(SiMe_3_)}^−^ (**3**‐**Ca** %*V*_Bur_=28.9‐29.9 %, Table S8, Figure S50) and {N(SiMe_3_)_2_}^−^ (**1**‐**Ca** and **2**‐**Ca** %*V*_Bur_=29.0‐29.7 %, Table S8, Figures S45 and S48) are comparable.^[58]^ Analogous conclusions can be drawn also when comparing **1**‐**Sr** with **3**‐**Sr** (Tables S5 and S8).

### Computational analysis

**Scheme 3:**
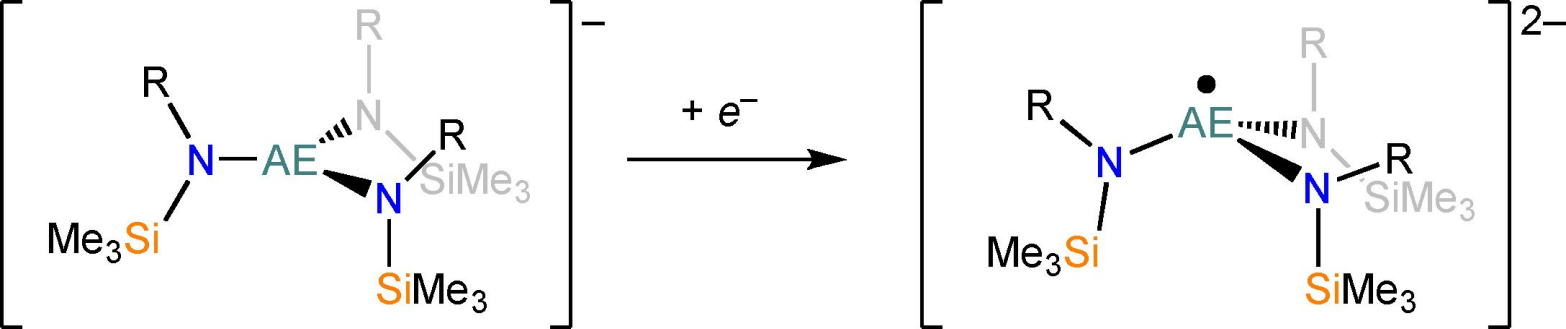
Proposed reduction of [AE{N(R)(SiMe_3_)}]^−^ anions (R=Mes, SiMe_3_) to [AE{N(R)(SiMe_3_)}]^2−^ radicals.

Density Functional Theory (DFT) studies were undertaken using Gaussian 16 with BP86 optimised structures, alongside electronic structure analyses completed with NBO7 (see Tables 1–2 and Supporting Information for full computational details). Relative free energies of the [AE{N(SiMe_3_)_2_}_3_]^−^ monoanion and the proposed reduced [AE{N(SiMe_3_)_2_}_3_]^2−^ dianion (Scheme 3) were calculated and confirm the reduction to be exergonic in nature (Ca: −20.5 kcal/mol; Sr: −20.2 kcal/mol; Ba: −22.1 kcal/mol – see Table S6 in Supporting Information).^[59]^ Molecular Orbital analysis of the [AE{N(SiMe_3_)_2_}_3_]^−^ monoanions and [AE{N(SiMe_3_)_2_}_3_]^2−^ dianions showed some *d*_z2_ character in a lowest‐unoccupied molecular orbital (specifically LUMO+6)^[60]^ of the monoanions (see Table 1). Natural Bond Orbital (NBO) calculations of the monoanion complexes in [AE{N(Mes)(SiMe_3_)}_3_]^−^ confirm that the LUMO has some *d*_z2_ character (NBO *d*%: 0.4–2.3; see Supporting Information), whilst in [AE{N(SiMe_3_)_2_}_3_]^−^ this is observed in higher MOs (LUMO+6; NBO *d*%: 1.3–2.1; see Supporting Information); the differing orbital profile could be tentatively ascribed to the structural differences between the two families of compounds, particularly with regards to C−N distances and involvement of aryl substituents (see above) in contrast to the silyl groups. Furthermore, the singly‐occupied molecular orbital, SOMO(α), of the dianionic reduced species shows electron density residing on the AE metal centre (as shown in Table 2), with a higher *d‐*character in [AE{N(Mes)(SiMe_3_)}_3_]^2−^ (NBO *d*%: 6.8–10.9 %; see Supporting Information) compared to [AE{N(SiMe_3_)_2_}_3_]^2−^ (NBO *d*%: 3.1–3.8 %; see Supporting Information). The optimised geometries of these reduced AE(I) dianionic species show a disruption of the trigonal planar geometry known for the [AE{N(SiMe_3_)_2_}_3_]^−^ and [AE{N(Mes)(SiMe_3_)}_3_]^−^ monoanions into a puckered, pyramidal geometry. In general, for Ca−Ba the extent of the pyramidalization increases together with the metal radius and is more pronounced in the [AE{N(Mes)(SiMe_3_)}_3_]^2−^ series, with [Ca{N(Mes)(SiMe_3_)}_3_]^2−^ showing the smallest deviation from a trigonal planar geometry (Ca⋅⋅⋅N^N^N=0.378 Å, Table S8). These structural differences could be due to mesityl substituent conformations in each amide ligand as observed in precursors **3**‐**AE** (see above), with this puckering affecting an increase or decrease in the *d‐*character of calculated SOMOs based on steric effects. A similar puckering was also observed in the solid state structure of [Y{N(SiMe_3_)_2_}_3_]^−^ ([Kr]4*d* ^1^) reported by Evans and co‐workers,^[41]^ which could be considered as isoelectronic to [Sr{N(SiMe_3_)_2_}_3_]^2−^. Conversely, [Sc{N(SiMe_3_)_2_}_3_]^−^ ([Ar]4*d* ^1^), isoelectronic to [Ca{N(SiMe_3_)_2_}_3_]^2−^, displays a regular trigonal planar geometry.^[39]^

**Table 1:** Free energies (BP86‐D3BJ(THF)/BS2//BP86/BS1 in kcal/mol) for reduction of [AE{N(SiMe_3_)_2_}]^−^ to [AE{N(SiMe_3_)_2_}]^2−^ and relevant MO diagrams for each species (MO energies in eV).

Metal	ΔG, AE^II/I^ (kcal/mol)	LUMO+6 AE(II) [AE{N(SiMe_3_)_2_}_3_]^−^	SOMO AE(I) [AE{N(SiMe_3_)_2_}_3_]^2−^
Mg^[a]^	−14.5	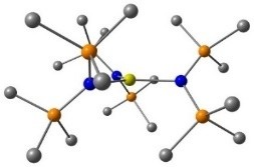	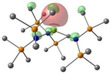
		1.47	2.75
Ca	−20.5	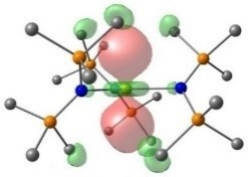	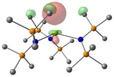
		2.23	2.83
Sr	−20.2	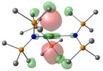	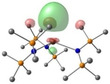
		2.18	2.86
Ba^[b]^	−22.1	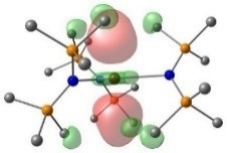	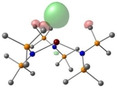
		2.07	2.78

[a] LUMO+6 of [Mg{N(SiMe_3_)_2_}_3_]^−^ (**1**‐**Mg**/**2**‐**Mg**) is not equivalent, so picture of LUMO is reported in this table. [b] structure for Ba built from **1**‐**Sr** geometry.

**Table 2:** Free energies (BP86‐D3BJ(THF)/BS2//BP86/BS1 in kcal/mol) for reduction of [AE{N(Mes)(SiMe_3_)}_3_]^−^ to [AE{N(Mes)(SiMe_3_)}_3_]^2−^ and relevant MO diagrams for each species (MO energies in eV).

Metal	ΔG, AE^II/I^ (kcal/mol)	LUMO AE(II) [AE{N(Mes)(SiMe_3_)}_3_]^−^	SOMO AE(I) [AE{N(Mes)(SiMe_3_)}_3_]^2−^
Mg^[a]^	−32.9	–	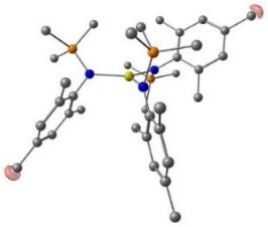
			2.50
Ca	−23.9	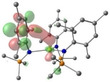	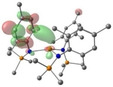
		1.39	2.80
Sr	−21.6	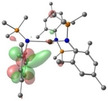	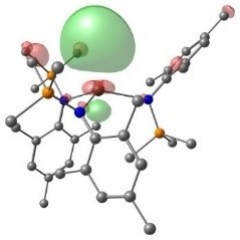
		1.36	2.67
Ba^[b]^	−6.6	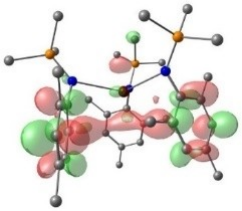	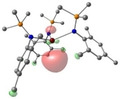
		1.28	2.56

[a] Computed Mg shown is based on **3**‐**Mg** with only two of the three Mesityl substituents of the amide ligands pointing in the same direction, with weak agostic Mg⋅⋅⋅Me_Si_ interactions. The LUMO has no notable interesting features and hence is not given. A conformation equivalent to **3**‐**Ca** (with all three Mesityl groups pointing in the same direction) is given in the Supporting Information and has a ΔG of −16.8 kcal/mol. [b] Computed Ba structure obtained by removing THF from **3‐Ba** ⋅ (**THF)** (see Supporting Information, ΔG obtained from THF‐free species to compare directly with Mg−Sr entries.

For completeness, the same computational studies were performed on Mg analogues, which also afforded an exergonic reaction profile for the reduction to Mg(I) (−14.5 kcal/mol), though to a lesser extent compared to the heavier analogues, and reassuringly no *d‐*orbital contributions observed in the MOs. Interestingly, in the case of [AE{N(Mes)(SiMe_3_)}_3_]^−^ monoanions the ΔG of the reduction is affected by the orientation of the mesityl substituents. For the Mg analogue, a large ΔG value (−32.9 kcal/mol) is obtained from the conformation with two mesityl substituents below the coordination plane and one situated above. Conversely, a smaller ΔG value (−16.8 kcal/mol) is obtained by adopting a conformation where all three mesityl substituents are positioned above the coordination plane as seen in **3**‐**Ca**. The latter is more in line with the energies calculated for the [AE{N(SiMe_3_)}_3_]^−/2−^ series, thus highlighting the role played by the ligand substituents and their conformations in the stability of reduced species.

**Scheme 4:**
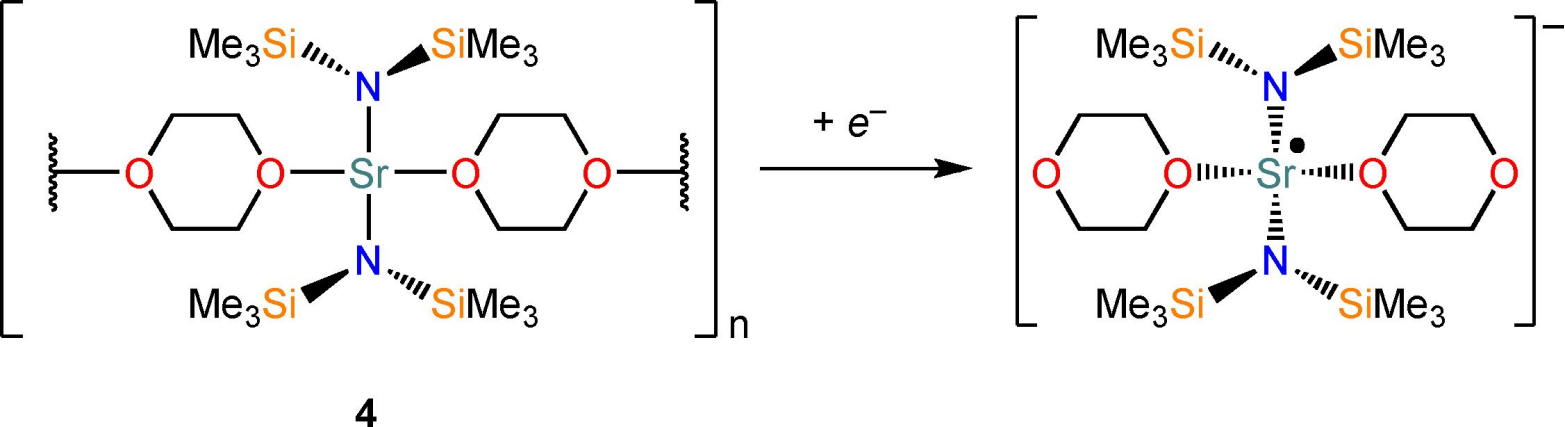
Proposed reduction of **4** to [Sr{N(SiMe_3_)_2_}_2_(dioxane)_2_]^−^ radical.

Owing to the results obtained with trigonal planar AE *tris‐*amide complexes, we postulated that a more generalised equatorial ligand field could induce an increase in *d*‐character for the LUMO of divalent Ca, Sr and Ba complexes, thus opening more possibilities in terms of ligand design. To verify this, we turned our attention towards complexes featuring a square planar coordination geometry. Square planar Mg(II) porphyrin complexes are relatively common, owing to the rigid conformation of the ligand dictating the geometry around the metal center;[^61^, ^62^, ^63^, ^64^, ^65^, ^66^, ^67^, ^68^, ^69^] however, these species are not suitable for our molecular design because of the redox activity of porphyrins and their incompatibility with large AE metals. Besides constrained square planar porphyrin complexes, a square planar geometry is a very rare coordination mode for the AE metals owing to the lack of crystal field stabilization. As a result there are a handful of near square planar magnesium complexes supported by nitrogen‐based ligands in the literature,[^70^, ^71^, ^72^, ^73^, ^74^, ^75^] and, to the best of our knowledge, only one example of a heavy AE complex in a square planar geometry, [Sr{N(SiMe_3_)_2_}_2_(dioxane)_2_]_n_ (**4**) reported by Lappert and co‐workers.^[76]^ Therefore, we decided to use **4** as a model for expanding our strategy through computational studies. Gratifyingly, MO analysis affords a LUMO with *d‐*character, which was also confirmed by NBO calculations (*d*%: 1.3). The computed reduction of **4** to monovalent [Sr{N(SiMe_3_)_2_}_2_(dioxane)_2_]^−^ (Scheme 4) is exergonic (−26.1 kcal/mol) and the resulting SOMO(α) also displays *d*‐character (*d*%: 7.8, Table 3 and Supporting Information). Analogously to the [AE{N(R)(SiMe_3_)}_3_]^−^ radicals, the optimised geometry of [Sr{N(SiMe_3_)_2_}_2_(dioxane)_2_]^−^ exhibits a slight pyramidalization (Sr⋅⋅⋅N^O^N^O=0.565 Å) compared to the structure of the divalent precursor **4** (Sr⋅⋅⋅N^O^N^O=0.056 Å, Table S8); and in this case, this structural puckering seems necessary to accommodate the presence of the additional charge.

**Table 3:** Free energies (BP86‐D3BJ (THF)/BS2//BP86/BS1 in kcal/mol) for reduction of [Sr{N(SiMe_3_)_2_}_2_(dioxane)_2_] to [Sr{N(SiMe_3_)_2_}_2_(dioxane)_2_]^−^ and relevant MO diagrams for each species (MO energies in eV).

ΔG, AE^II/I^ (kcal/mol)	LUMO Sr(II) [Sr{N(SiMe_3_)_2_}_2_(dioxane)_2_]	SOMO Sr(I) [Sr{N(SiMe_3_)_2_}_2_(dioxane)_2_]^−^
−26.1	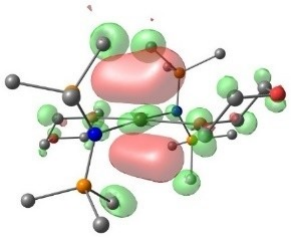	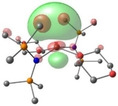
	−1.01	0.84

## Discussion

Our modelling shows that the constrained structural geometries imposed by sterically demanding ligands can have a significant effect on the energy of *d‐*orbitals, even in the case of the AE metals for which there are negligible crystal field effects. Though NBO analysis reveals a predominant *s*‐character for the LUMO and SOMO(α) in all precursors and reduced species respectively, the involvement of *d‐*orbitals in frontier orbitals is significant. It is noteworthy that *d‐*character in Sc^2+^, Y^2+^ and La^2+^ species (isoelectronic with Ca^+^, Sr^+^ and Ba^+^ respectively) has been evaluated via X‐ray absorption methods,^[42]^ but, to the best of our knowledge, details of NBO or Mulliken analysis of frontier orbitals have not been reported. In the case of the Lu^2+^ complex [K(crypt)][Lu(Cp′)_3_] ([Xe]5*f* ^14^4*d* ^1^) Mulliken population showed 42 % metal character and 48 % *d‐*character in the SOMO(α).^[34]^ In our studies, the NBO analysis of the SOMO(α) of [AE{N(R)(SiMe_3_)}_3_]^2−^ and [Sr{N(SiMe_3_)_2_}_2_(dioxane)_2_]^−^ shows varying degrees of *d‐*orbital participation, with additional *p‐*orbital contribution as high as 31 % in [Ba{N(SiMe_3_)_2_}_3_]^2−^ (see Supporting Information). This is in part reminiscent of *sp* ^3^ *d* and *sp* ^3^ *d* ^2^‐like hybridizations that have been previously invoked to describe deviation from linearity in the geometry of AE metallocenes and halides,[^77^, ^78^, ^79^, ^80^, ^81^] but have not been taken into consideration as a potential characteristic of monovalent AE species.

Following our computational analysis, we carried out several attempts to reduce **1**‐**AE** and **3**‐**AE**, however we have not been able to produce our target reduced species. In the case of **1**‐**Ca**, we performed attempted reductions with KC_8_ either with 18‐crown‐6 in THF, or in benzene without any sequestering agent (for the latter we used [Ca{N(SiMe_3_)_2_}_3_K] as starting material, rather than **1**‐**Ca**). In both cases we recovered unreacted starting material and K[N(SiMe_3_)_2_], together with trace amounts of other unknown species that we could not identify. These results were obtained even under strict temperature control in the attempt to minimize decomposition reactions (see Supporting Information Figures S22 and S25). Similarly, when **3**‐**Ca** was reacted under the same conditions and ^1^H NMR analysis reaction mixtures revealed the presence of unreacted starting material and K[N(Mes)(SiMe_3_)], together with other unknown species (see Supporting Information Figures S23 and S27). We also attempted the reductions of **1**‐**Sr** and **3**‐**Sr** obtaining similar results (Figures S24 and S26). Furthermore, we attempted the reduction of **4** in various conditions, but also in this case we either recovered unreacted starting material or obtained an intractable mixture of products (see Supporting Information Figures S28 and S29).

Reasons for the lack of success with these reductions could be ascribed to the reducing power of the reagents employed and also the incompatibility of the ligand systems with reductive chemistry, owing to the facile elimination of potassium amide by‐products and the propensity of silylamide ligands to undergo facile degradations via intra‐ and intermolecular C−H activation.[^40^, ^82^] Additionally, the ligands used in this work display various degrees of flexibility, which can affect the energy profile of the reductions and stability of the resultant reduced species. The stabilization of dianionic complexes as a result of these reductions (e.g. [AE{N(SiMe_3_)(R)}_3_]^2−^) could also be deemed problematic in terms of charge distribution. Therefore, the use of neutral precursors would be highly desirable, as demonstrated by the more favourable reactivity profile of the reduction of [Sr{N(SiMe_3_)_2_}_2_(dioxane)_2_]_n_. Despite the lack of success in isolating monovalent AE complexes, our theoretical modelling based on trigonal planar and square planar precursors provides a blueprint for further exploitation in synthetic chemistry. Nonetheless, more suitable ligand systems should be employed to implement this blueprint, which needs to possess a relatively innocent redox profile, paired with sufficient conformational rigidity and steric protection to enforce highly equatorial geometries.

## Conclusions

We have reported the synthesis of a series of trigonal planar, heavy AE(II) silylamide complexes; DFT calculations have been performed on these complexes and the theoretical AE(I) species that could form upon chemical reduction. We have demonstrated that Ca(II), Sr(II) and Ba(II) complexes with highly equatorial ligand environments (i. e. trigonal planar, square planar) have readily accessible *d_z2_* orbitals, and that these should be occupied for their respective AE(I) analogues, providing feasible synthetic targets. Together, this work has presented a combined synthetic strategy and theoretical modelling that provide a blueprint for targeting sub‐valent heavy AE species that do not form AE−AE bonds. This approach may provide heavy AE(I) complexes that should exhibit rich reductive chemistry analogous to that of divalent REs Sc, Y and La.[^39^, ^40^, ^41^] We are exploring ligand variations and reduction protocols, including different reducing agents and the use of solid‐state reactions, in order to overcome the synthetic challenges that have hampered the isolation of heavy AE(I) complexes to date.

## Experimental Section

**General methods**: THF and toluene were passed through columns containing molecular sieves, then stored over a potassium mirror (toluene), or over 4 Å molecular sieves (THF) and thoroughly degassed prior to use. Hexane and diethyl ether were purchased anhydrous from Tokyo Chemical Industry, dried over activated molecular sieves for 7 days, then stored over a potassium mirror. For NMR spectroscopy, C_6_D_6_ and C_4_D_8_O were dried by refluxing over potassium, and then vacuum transferred and degassed by three freeze‐pump‐thaw cycles before use. NMR spectra were recorded on either a Bruker Avance III HD 400 or Bruker Avance III 500 spectrometer operating at 400.07/500.13 (^1^H), 100.60/125.78 (^13^C{^1^H}) or 79.48 (^29^Si{^1^H}) MHz. To achieve a greater signal to noise ratio, the ^29^Si{^1^H} NMR spectra were acquired with a DEPT24 pulse sequence. NMR spectra were recorded at 298 K unless otherwise stated and were referenced to residual solvent signals in the case of ^1^H and ^13^C{^1^H} experiments, or externally referenced to SiMe_4_ (^29^Si{^1^H}). FTIR spectra were recorded on a Bruker Alpha II spectrometer with Platinum‐ATR module. Elemental microanalyses were carried out by the Elemental Analysis Service at London Metropolitan University. ^n^BuLi was used as received. 18‐crown‐6 was dissolved in anhydrous diethyl ether and dried over 4 Å molecular sieves for 7 days before removing the solvent *in vacuo* and drying under vacuum. [2.2.2]‐cryptand was dried *in vacuo* for 4 h prior to use. All AE iodides were baked at 200 °C for 4 h prior to use. HN(Mes)(SiMe_3_),^[83]^ K[N(Mes)(SiMe_3_)],^[84]^ K[N(SiMe_3_)_2_],^[85]^ **1‐Ca** ^[86]^ and **4** ^[87]^ were prepared according to literature procedures.

## Synthesis

**[K(18**‐**c**‐**6)_2_][Mg{N(SiMe_3_)_2_}_3_] and [K(18**‐**c**‐**6)][Mg{N(SiMe_3_)_2_}_3_] (1**‐**Mg)**: [Mg{N(SiMe_3_)_2_}_3_K] (0.272 g, 0.50 mmol, 1 equiv.) and 18‐crown‐6 (0.198 g, 0.75 mmol, 1.5 equiv.) were weighed in a Schlenk flask, before toluene (25 mL) was added and the resulting solution stirred for 16 h at room temperature. All volatiles were removed *in vacuo* yielding an oily, colourless solid which was washed thoroughly with hexane (3 x 10 mL) portions before being dried under reduced pressure, yielding [K(18‐c‐6)_2_][Mg{N(SiMe_3_)_2_}_3_] as a colourless powder (0.258 g, 0.240 mmol, 48 %). Crystals of **1**‐**Mg** suitable for X‐ray diffraction were obtained from layering a concentrated toluene solution (0.300 g in 3 mL) with hexane at room temperature.

^1^H NMR (500 MHz, 298 K, C_6_D_6_): δ/ppm=0.71 (s, 54H, Si(C*H*_3_)_3_), 3.16 (s, 48H, OC*H*_2_). ^13^C{^1^H} NMR (125 MHz, 298 K, C_6_D_6_) δ/ppm=7.36 (Si(*C*H_3_)_3_), 70.63 (O*C*H_2_). No signals could be obtained from ^29^Si{^1^H} NMR experiments.

FTIR: $\tilde{\nu }$ /cm^−1^=2941(w), 2893 (w), 1351 (w), 1234 (m), 1105 (s), 1000 (s), 963 (m), 876 (m), 822 (s), 660 (m), 609 (w), 442 (w).

Anal. calcd. for C_30_H_78_N_3_KMgO_6_Si_6_: C, 44.55 %; H, 9.72 %; N, 5.19 %. Found: C, 44.47 %; H, 10.03 %; N, 5.00 %.

**[K(18**‐**c**‐**6)(C_7_H_8_)(THF)][Sr{N(SiMe_3_)_2_}_3_] (1**‐**Sr)**: [Sr{N(SiMe_3_)_2_}_3_K] (0.20 g, 0.329 mmol, 1 equiv.) and 18‐crown‐6 (0.082 g, 0.313 mmol, 0.95 equiv.) were weighed in a Schlenk flask, before toluene (25 mL) was added and the resulting solution stirred for 72 h at room temperature. All volatiles were removed *in vacuo*, and the resulting colourless solid material was washed thoroughly with hexane (3 x 10 mL) and dried under reduced pressure, yielding **1**‐**Sr** as a colourless powder (0.125 g, 0.14 mmol, 46 %). Crystals of **1**‐**Sr** suitable for X‐ray diffraction were obtained from a concentrated solution (0.350 g in 5 mL of toluene) stored at −25 °C.

^1^H NMR (400 MHz, 298 K, C_6_D_6_): δ/ppm=0.60 (s, 54H, Si(C*H*_3_)_3_), 2.89 (s, 24H, OC*H*_2_). ^13^C{^1^H} NMR (100 MHz, 298 K, C_6_D_6_): δ/ppm=6.34 (Si(*C*H_3_)_3_), 69.76 (O*C*H_2_). ^29^Si{^1^H} NMR (80 MHz, 298 K, C_6_D_6_): δ/ppm=−18.2.

FTIR: $\tilde{\nu }$ /cm^−1^=2940 (w), 2891 (w), 1350 (w), 1235 (m), 1105 (s), 1002 (m), 961 (m), 879 (m), 815 (s), 753 (w), 656 (m), 426 (w).

Anal. calcd. for C_30_H_78_N_3_KO_6_Si_6_Sr: C, 41.31 %; H, 9.01 %; N, 4.82 %. Found: C, 41.11 %; H, 8.65 %; N, 4.33 %.

**[K(crypt)][Mg{N(SiMe_3_)_2_}_3_] (2**‐**Mg)**: [Mg{N(SiMe_3_)_2_}_3_K] (0.096 g, 0.177 mmol, 1 equiv.) and [2.2.2]‐cryptand (0.100 g, 0.265 mmol, 1.5 equiv.) were weighed in a Schlenk flask, then toluene (25 mL) was added and the resulting solution stirred overnight at room temperature. All volatiles were removed *in vacuo*, and the resulting colourless solid material was washed thoroughly with hexane (3 x 10 mL) and dried under reduced pressure, yielding **2**‐**Mg** as a colourless powder (0.094 g, 0.10 mmol, 58 %). Crystals of **2**‐**Mg** suitable for X‐ray diffraction were obtained from slow evaporation of a concentrated solution (0.094 g in 1 mL of toluene) stored at room temperature.

^1^H NMR (400 MHz, 298 K, C_6_D_6_): δ/ppm=0.76 (s, 54H, Si(C*H*_3_)_3_), 1.91 (m, 12H, C*H*_2_‐N), 2.91 (m, 12H, O‐C*H*_2_‐CH_2_‐N), 3.02 (s, 12H, O‐(C*H*_2_)_2_). ^13^C{^1^H} NMR (100 MHz, 298 K, C_6_D_6_): δ/ppm=7.4 (Si(*C*H_3_)_3_), 53.6 (*C*H_2_‐N), 67.4 (O‐*C*H_2_‐CH_2_‐N), 70.4 (O‐(*C*H_2_)_2_). ^29^Si{^1^H} NMR (80 MHz, 298 K, C_6_D_6_): δ/ppm=−10.0.

FTIR: $\tilde{\nu }$ /cm^−1^=2936 (w), 2884 (w) 1353 (w), 1235 (w), 1105 (s), 1000 (s), 876 (m), 817 (s), 749 (w), 660 (m), 422 (w).

Anal. calcd. for C_36_H_90_N_5_KMgO_6_Si_6_ ⋅ (C_7_H_8_)_0.5_: C, 49.06 %; H, 9.80 %; N, 7.24 %. Found: C, 49.63 %; H, 9.57 %; N, 7.33 %.

**[K(crypt)][Ca{N(SiMe_3_)_2_}_3_] (2**‐**Ca)**: [Ca{N(SiMe_3_)_2_}_3_K] (0.149 g, 0.266 mmol, 1 equiv.) and [2.2.2]‐cryptand (0.100 g, 0.265 mmol, 1 equiv.) were weighed in a Schlenk flask, then toluene (25 mL) was added and the resulting solution stirred for 6 h at room temperature. All volatiles were removed *in vacuo*, and the resulting colourless solid was washed thoroughly with hexane portions (2×10 mL) and dried under reduced pressure, yielding **2**‐**Ca** as a colourless powder (0.181 g, 0.19 mmol, 72 %). Crystals of **2**‐**Ca** suitable for X‐ray diffraction were obtained from layering a concentrated benzene solution (0.165 g in 2 mL) with hexane at room temperature.

^1^H NMR (400 MHz, 298 K, C_6_D_6_): δ/ppm=0.69 (s, 54H, Si(C*H*_3_)_3_), 1.92 (m, 12H, C*H*_2_‐N), 2.92 (m, 12H, O‐C*H*_2_‐CH_2_‐N), 3.03 (s, 12H, O‐(C*H*_2_)_2_). ^13^C{^1^H} NMR (100 MHz, 298 K, C_6_D_6_): δ/ppm=6.6 (Si(*C*H_3_)_3_), 53.6 (*C*H_2_‐N), 67.4 (O‐*C*H_2_‐CH_2_‐N), 70.4 (O‐(*C*H_2_)_2_). ^29^Si{^1^H} NMR (80 MHz, 298 K, C_6_D_6_): δ/ppm=−15.9.

FTIR: $\tilde{\nu }$ /cm^−1^=2938 (w), 2886 (w), 1354 (w), 1104 (s), 1054 (m), 951 (w), 876 (m), 814 (s), 748 (m), 658 (m), 589 (w), 425 (w).

Anal. calcd. for C_36_H_90_N_5_CaKO_6_Si_6_ ⋅ (C_6_H_14_)_0.1_: C, 46.16 %; H, 9.68 %; N, 7.48 %. Found: C, 46.50 %; H, 9.74 %; N, 7.41 %.

**[Mg{N(Mes)(SiMe_3_)}_3_K]_∞_ (3**‐**Mg)**: MgI_2_ (0.278 g, 1 mmol, 1 equiv.) and K[N(Mes)(SiMe_3_)] (0.735 g, 3 mmol, 3 equiv.) were weighed in a Schlenk flask, then THF (25 mL) was added at room temperature yielding a colourless suspension which was stirred for 72 h before being filtered. All volatiles were removed from the filtrate *in vacuo* without the application of heat, affording a colourless solid which was washed with toluene (3×5 mL) and dried under reduced pressure, yielding **3**‐**Mg** as a colourless powder (0.105 g, 0.0.162 mmol, 16 %). Crystals of **3**‐**Mg** suitable for X‐ray diffraction were obtained from layering a concentrated toluene solution (0.100 g in 2 mL) with hexane at room temperature.

^1^H NMR (400 MHz, 298 K, C_6_D_6_): δ/ppm=0.41 (s, 27H, Si(C*H*_3_)_3_), 2.01 (s, 9H, *para*‐C*H*_3_), 2.51 (s, 18H, *ortho*‐C*H*_3_), 6.60 (s, 6H, Ar‐*H*). ^13^C{^1^H} NMR (100 MHz, 298 K, C_6_D_6_) δ/ppm=5.1 (Si(*C*H_3_)_3_), 20.5 (*para*‐*C*H_3_), 22.8 (*ortho*‐*C*H_3_), 125.0 (Ar‐*C*), 130.0 (Ar‐*C*), 134.1 (Ar‐*C*), 155.1 (Ar‐*C*). ^29^Si{^1^H} NMR (80 MHz, 298 K, C_6_D_6_): δ/ppm=−8.9.

FTIR: $\tilde{\nu }$ /cm^−1^=2939 (w), 1467 (s), 1340 (m), 1227 (s), 1154 (w), 982 (m), 919 (m), 809 (s), 658 (m), 566 (w), 521 (w).

Anal. calcd. for C_36_H_60_N_3_KMgSi_3_: C, 63.35 %; H, 8.86 %; N, 6.16 %. Found: C, 59.36 %; H, 8.44 %; N, 5.38 %. Low carbon values were obtained consistently across different measurements and we ascribe this to carbide formation, which has been previously reported in analogous silicon‐rich Group 2 complexes.[^88^, ^89^]

**[Ca{N(Mes)(SiMe_3_)}_3_K]_∞_ (3**‐**Ca)**: CaI_2_ (0.885 g, 3 mmol, 1 equiv.) and K[N(Mes)(SiMe_3_)] (2.206 g, 9 mmol, 3 equiv.) were weighed in a Schlenk flask, then diethyl ether (30 mL) was added and the resulting suspension stirred at room temperature for 48 h. The reaction mixture was filtered and a further 15 mL of diethyl ether used to wash the insoluble material. All volatiles were removed from the combined filtrates *in vacuo*, yielding **3**‐**Ca** as a colourless solid (1.943 g, 2.78 mmol, 92.7 %). Crystals of **3**‐**Ca** suitable for X‐ray diffraction were obtained from a concentrated solution (0.800 g in 10 mL of toluene) stored at room temperature.

^1^H NMR (400 MHz, 298 K, C_6_D_6_): δ/ppm=0.33 (s, 27H, Si(C*H*_3_)_3_), 2.05 (s, 9H, *para*‐C*H*_3_), 2.41 (s, 18H, *ortho*‐C*H*_3_), 6.65 (s, 6H, Ar‐*H*). ^13^C{^1^H} NMR (100 MHz, 298 K, C_6_D_6_): δ/ppm=4.7 (Si(*C*H_3_)_3_), 20.5 (*para*‐*C*H_3_), 22.3 (*ortho*‐*C*H_3_), 124.1 (Ar‐*C*), 129.7 (Ar‐*C*), 132.1 (Ar‐*C*), 155.0 (Ar‐*C*). ^29^Si{^1^H} NMR (80 MHz, 298 K, C_6_D_6_): δ/ppm=−15.7.

FTIR: $\tilde{\nu }$ /cm^−1^=2937 (w), 1417 (m), 1295 (m), 1254 (s), 1158 (w), 960 (m), 923 (s), 817 (s), 773 (m), 736 (w), 660 (m), 485 (m).

Anal. calcd. for C_36_H_60_N_3_CaKSi_3_: C, 61.92 %; H, 8.66 %; N, 6.02 %. Found: C, 61.92 %; H, 8.83 %; N, 5.94 %.

**[Sr{N(Mes)(SiMe_3_)}_3_K]_∞_ (3**‐**Sr)**: SrI_2_ (1.023 g, 3 mmol, 1 equiv.) and K[N(Mes)(SiMe_3_)] (2.206 g, 9 mmol, 3 equiv.) were weighed in a Schlenk flask, then diethyl ether (30 mL) was added and the resulting suspension stirred at room temperature for 48 h. The reaction mixture was filtered and a further 15 mL of diethyl ether used to wash the insoluble material. All volatiles were removed from the combined filtrates *in vacuo*, yielding **3**‐**Sr** as a colourless solid (1.943 g, 2.78 mmol, 92.7 %). Crystals of **3**‐**Sr** suitable for X‐ray diffraction were obtained from a concentrated solution (0.460 g in 10 mL of toluene) stored at room temperature.

^1^H NMR (400 MHz, 298 K, C_6_D_6_): δ/ppm=0.26 (s, 27H, Si(C*H*_3_)_3_), 2.04 (s, 9H, *para*‐C*H*_3_), 2.42 (s, 18H, *ortho*‐C*H*_3_), 6.68 (s, 6H, Ar‐*H*). ^13^C{^1^H} NMR (100 MHz, 298 K, C_6_D_6_): δ/ppm=4.6 (Si(*C*H_3_)_3_), 20.9 (*para*‐*C*H_3_), 21.2 (*ortho*‐*C*H_3_), 25.5 (THF‐*C*H_2_), 67.9 (THF‐O*C*H_2_), 129.7 (Ar‐*C*), 132.6 (Ar‐*C*), 133.8 (Ar‐*C*). No signals could be obtained from ^29^Si{^1^H} NMR experiments.

FTIR: $\tilde{\nu }$ /cm^−1^=2938 (w), 1412 (w), 1294 (m), 1237 (s), 1159 (w), 1036 (w), 962 (m), 927 (s), 814 (s), 767 (m), 736 (w), 661 (w), 476 (m).

Satisfactory elemental analysis could not be obtained despite repeated attempts on freshly prepared samples.

**[Ba{N(Mes)(SiMe_3_)}_3_K]_∞_ (3**‐**Ba)**: BaI_2_ (0.391 g, 1 mmol, 1 equiv.) and K[N(Mes)(SiMe_3_)] (0.735 g, 3 mmol, 3 equiv.) were weighed in a Schlenk flask, then diethyl ether (30 mL) was added and the resulting suspension stirred at room temperature for 48 h. The reaction mixture was filtered and a further 15 mL of diethyl ether used to wash the insoluble material. All volatiles were removed from the combined filtrates *in vacuo*, and the resulting solid washed with hexane portions (2×10 mL) yielding **3**‐**Ba** as a colourless solid (0.492 g, 0.62 mmol, 62.0 %). Crystals of **3**‐**Ba** ⋅ **(THF)** suitable for X‐ray diffraction were obtained from a concentrated solution (0.460 g in 10 mL of toluene with <1 mL of THF) stored at room temperature.

^1^H NMR (400 MHz, 298 K, C_6_D_6_): δ/ppm=0.36 (s, 27H, Si(C*H*_3_)_3_), 2.33 (s, 9H, *para*‐C*H*_3_), 2.36 (s, 18H, *ortho*‐C*H*_3_), 6.98 (s, 6H, Ar‐*H*). ^13^C{^1^H} NMR (100 MHz, 298 K, C_6_D_6_): δ/ppm=5.0 (Si(*C*H_3_)_3_), 21.0 (*para*‐*C*H_3_), 21.2 (*ortho*‐*C*H_3_), 24.8 (THF‐*C*H_2_), 67.0 (THF‐O*C*H_2_), 128.6), 129.0 (Ar‐*C*), 129.3 (Ar‐*C*), 130.2 (Ar‐*C*). No signals could be obtained from ^29^Si{^1^H} NMR experiments.

FTIR: $\tilde{\nu }$ /cm^−1^=2940 (w), 1417 (m), 1301 (s), 1236 (m), 966 (m), 926 (s), 865 (w), 814 (s), 735 (m), 654 (m), 458 (w).

Anal. calcd. for C_36_H_60_N_3_BaKSi_3_: C, 54.35 %; H, 7.60 %; N, 5.28 %. Found: C, 53.45 %; H, 7.28 %; N, 4.87 %. Low carbon values were obtained consistently across different measurements and we ascribe this to carbide formation, which has been previously reported in analogous silicon‐rich Group 2 complexes.[^88^, ^89^]

**Computational method**: DFT calculations were run with Gaussian 16 (C.01).^[90]^ The Mg, Ca, Sr, Ba and Si centres were described with the Stuttgart RECPs and associated basis sets,^[91]^ and the 6‐31G** basis sets were used for all other atoms (BS1).^[92]^ A polarization function was also added to Si (ζ_d_=0.284). Initial BP86^[93]^ optimizations were performed using the ‘grid=ultrafine’ option, with all stationary points being fully characterized via analytical frequency calculations as minima (all positive eigenvalues). All energies were recomputed with a larger basis set featuring 6‐311++G** on all atoms. Corrections for the effect of THF (ϵ=7.4257) solvent were run using the polarizable continuum model and BS1.^[94]^ Single‐point dispersion corrections to the BP86 results employed Grimme's D3 parameter set with Becke‐Johnson damping as implemented in Gaussian.^[95]^ Natural Bonding Orbital (NBO7)^[96]^ analyses were performed on the BP86/BS1‐optmised geometries with a larger basis set featuring 6‐311++G** on all atoms, with the exceptions of Ca (cc‐pVTZ), Sr and Ba (both def2‐TVZP), within Gaussian 16 (C.01).

## Supporting Information

Additional experimental details, full characterization data, crystallographic details and computational analysis are reported in the Supporting Information. The authors have cited additional references within the Supporting Information.[^97^, ^98^, ^99^, ^100^, ^101^, ^102^, ^103^, ^104^] Deposition Numbers 2259496 (for [Sr{N(SiMe_3_)_3_}K]), 2259497 (for **1**‐**Mg**), 2259498 (for **2**‐**Ca**), 2259499 (for **3**‐**Sr**), 2259500 (for **3**‐**Ba**), 2259501 (for **3**‐**Mg**), 2259502 (for **1**‐**Sr**), 2259503 (for **2**‐**Mg**), 2259504 (for **3**‐**Ca**), 2259802 (for **3**‐**Ca ⋅ (THF)**) contain the supplementary crystallographic data for this paper. These data are provided free of charge by the joint Cambridge Crystallographic Data Centre and Fachinformationszentrum Karlsruhe Access Structures service.

## Conflict of interest

The authors declare no conflict of interest.

1

## Supporting information

As a service to our authors and readers, this journal provides supporting information supplied by the authors. Such materials are peer reviewed and may be re‐organized for online delivery, but are not copy‐edited or typeset. Technical support issues arising from supporting information (other than missing files) should be addressed to the authors.

Supporting Information

## Data Availability

The data that support the findings of this study are openly available in Figshare at https://doi.org/10.25392/leicester.data.22928210, reference number 22928210.
