# Supplementary material for: A Blueprint for the Stabilization of Sub‐Valent Alkaline Earth Complexes
Source: Chemistry. 2023 Aug 14;29(54):e202301850. doi: 10.1002/chem.202301850 (PMC10947258; doi:10.1002/chem.202301850)
Supplement: Supplementary file 1 — Supporting Information [file CHEM-29-0-s001.pdf]

Chemistry–A European Journal

Supporting Information

A Blueprint for the Stabilization of Sub-Valent Alkaline Earth Complexes

Alex W. J. Bowles, Yu Liu, Matthew P. Stevens, Iñigo J. Vitorica-Yrezabal, Claire L. McMullin,* and Fabrizio Ortu*

S1. Experimental……………………………………………………………………………………S2

S2. NMR data…………………………………………………………………………………….S4

S3. IR data……………………………………………………………………………………...S19

S4. Crystallography……………………………………………………………………...S23

S5. Computational details…………………………………………………………………S30

S6. Buried volume calculations……………………………………………………….....S60

S7. References…………………………………………………………………………………S65

S1. Experimental

General method for the preparation of [AE{N(SiMe$_3$)$_2$}$_3$K]:[1] AEI$_2$ (3.05 mmol, 1.02 eq.) and K[N(SiMe$_3$)$_2$] (6 mmol, 3 eq.) were weighed in a Schlenk flask, then diethyl ether (30 mL) was added and the resulting suspension stirred for 24 hours at room temperature. The reaction mixture was filtered and the solvent removed from the filtrate *in vacuo* yielding a colourless solid which was washed thoroughly with hexane (3 x 10 mL) before being dried fully under reduced pressure, yielding [AE{N(SiMe$_3$)$_2$}$_3$K] as a colourless solid.

[Mg{N(SiMe$_2$)$_2$}$_3$K]: from 0.848 g of MgI$_2$ (3.05 mmol), yield 1.453 g (2.67 mmol, 88.9%).

[Ca{N(SiMe$_2$)$_2$}$_3$K]: from 0.588 g of CaI$_2$ (2 mmol), yield 0.955 g (1.70 mmol, 85.2%).

[Sr{N(SiMe$_2$)$_2$}$_3$K]: from 1.041 g of SrI$_2$ (3.05 mmol), yield 1.323 g (2.18 mmol, 72.6%). Single crystals suitable for X-ray diffraction studies were obtained from a concentrated solution (0.185 g in 30 mL of hexane) stored at room temperature.

*Attempted reductions of **1-4**.*

Reduction of 1 and 3 - Method A: [AE{N(R)(SiMe$_3$)}$_3$K] (AE = Ca, Sr; R = Mes, SiMe$_3$) (0.5 mmol, 1 eq.) and KC$_8$ (1 mmol, 2 eq.) were weighed in a Schlenk flask charged with a glass-coated magnetic stirrer bar before benzene (30 mL) was added to the mixture at room temperature. The resulting suspension was stirred overnight at room temperature and then filtered, yielding a yellow solution and unreacted KC$_8$ in all cases. All volatile components were removed from the filtrate *in vacuo* yielding either an oily, off-white solid (AE = Ca, Sr; R = Mes) or a white powder (AE = Ca; R = SiMe$_3$). ^{1}H NMR spectra of crude mixtures are reported in Figures S22, S23, and S24.

Reduction of 1 and 3 - Method B: [AE{N(SiMe$_3$)$_2$}$_3$K] (AE = Ca-Sr) (0.5 mmol 1 eq.), 18-crown-6 (0.5 mmol, 1 eq.) and KC$_8$ or K/KI (1-2 mmol, 2-4 eq.) were combined in a Schlenk flask charged with a glass-coated magnetic stirrer bar; the flask was cooled to −40 °C and THF (30 mL) was added. The reduction of **3-Ca** was also attempted using the same conditions but with the absence of 18-crown-6. The solution was a deep-blue colour upon addition of THF for reactions containing crown-ether and colourless in the case of **3-Ca**. The reaction mixture was stirred for 2 hours at −35 °C before being filtered at low temperature (c.a. −78 °C). The filtrate

had lost all deep blue colour after 16 hours at −25 °C. All volatile components were removed from the resulting colourless solution yielding a white solid in all cases. ^{1}H NMR spectra of crude mixtures are reported in Figures S25, S26 and S27. In the case of the reaction of **3-Ca**, we obtained a crystalline crop of the correspondent THF adduct $[AE\{N(SiMe_3)_2\}_3(THF)\{K(THF)\}]_\infty$ (**3-Ca·(THF)$_2$**) from a saturated hexane solution (see Figure S41).

Reduction of 4 – Method C: To a Schlenk flask charged with a glass-coated magnetic stirrer bar, **4** (0.250 g, 0.4 mmol, 1 eq.) and KC_8 (0.135 g, 1 mmol, 2.3 mmol) were added before the addition of benzene (25 mL) at room temperature. The solution changed from colourless to yellow after approximately 15 minutes. The reaction mixture was stirred overnight at room temperature and then filtered. The filtrate was concentrated and stored at 8 °C, yielding colourless crystals overnight. The crystalline material was confirmed to be the starting material **4** *via* single crystal XRD studies. In another attempt, all volatiles were removed from the combined filtrates *in vacuo* yielding an off-white powder. The ^{1}H NMR spectrum of the crude product mixture is reported in Figure S28.

Reduction of 4 – Method D: To a Schlenk flask charged with a glass-coated magnetic stirrer bar, **4** (0.250 g, 0.4 mmol, 1 eq.), 18-crown-6 (0.113 g, 0.4 mmol, 1 eq.), K/KI (5% wt/wt, 0.837 g, 1.1 mmol, 2.5 eq.) were added before 1,4-dioxane (25 mL) was added at room temperature and stirred overnight. The reaction mixture was filtered and further portions of 1,4-dioxane were used to wash the remaining solids. All volatiles were removed from the combined filtrates *in vacuo,* yielding a colourless oily material. The ^{1}H NMR spectrum of the crude product mixture is reported in Figure S29.

S2. NMR data

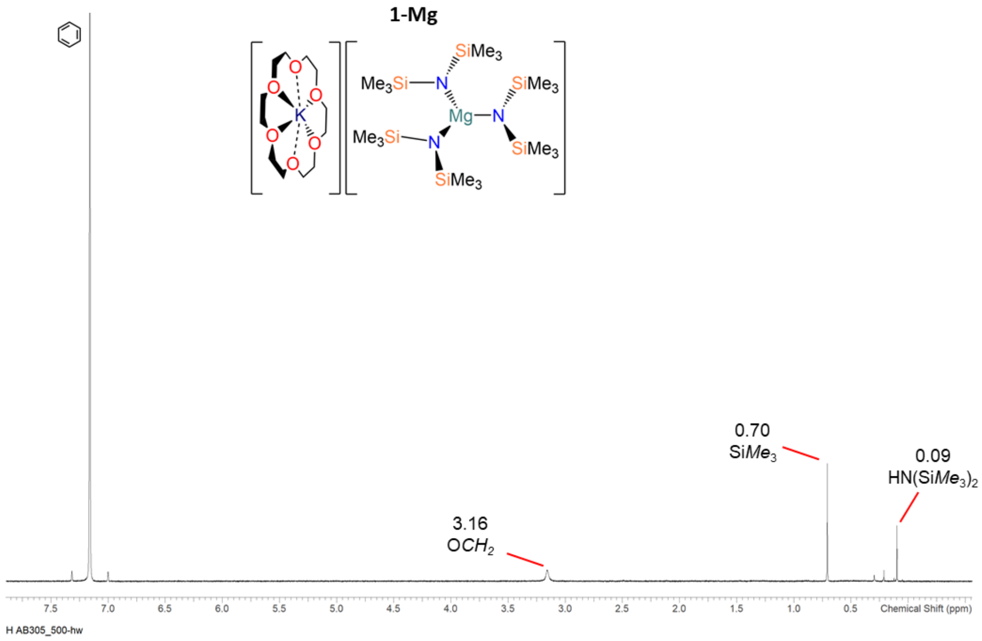

Figure S1: ^{1}H NMR (500 MHz, 298 K, C_6D_6) spectrum of **1-Mg**, with assignment.

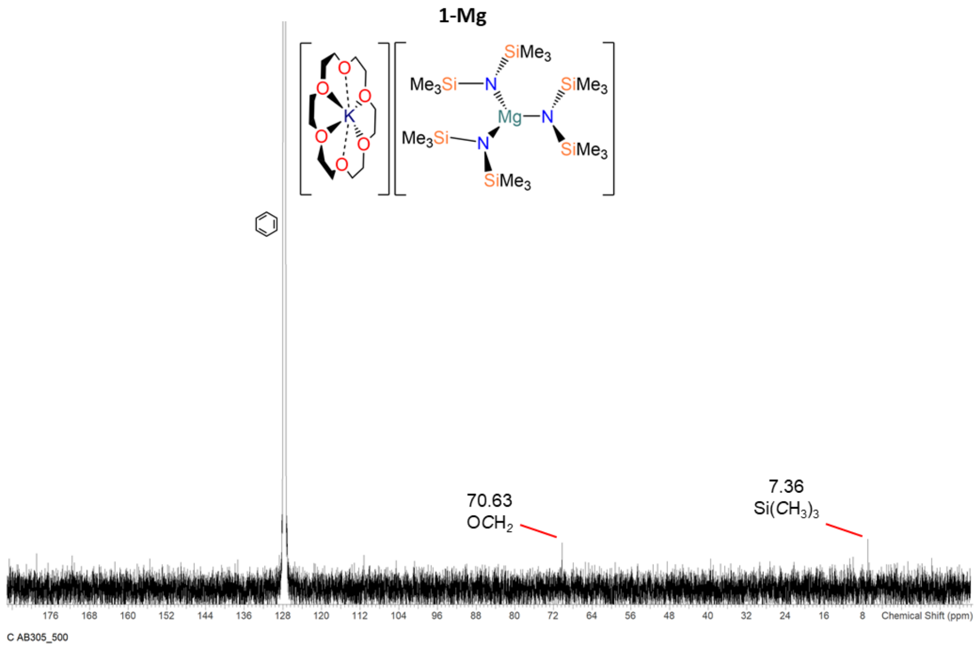

Figure S2: $^{13}C\{^{1}H\}$ NMR (125 MHz, 298 K, C_6D_6) spectrum of **1-Mg**, with assignment.

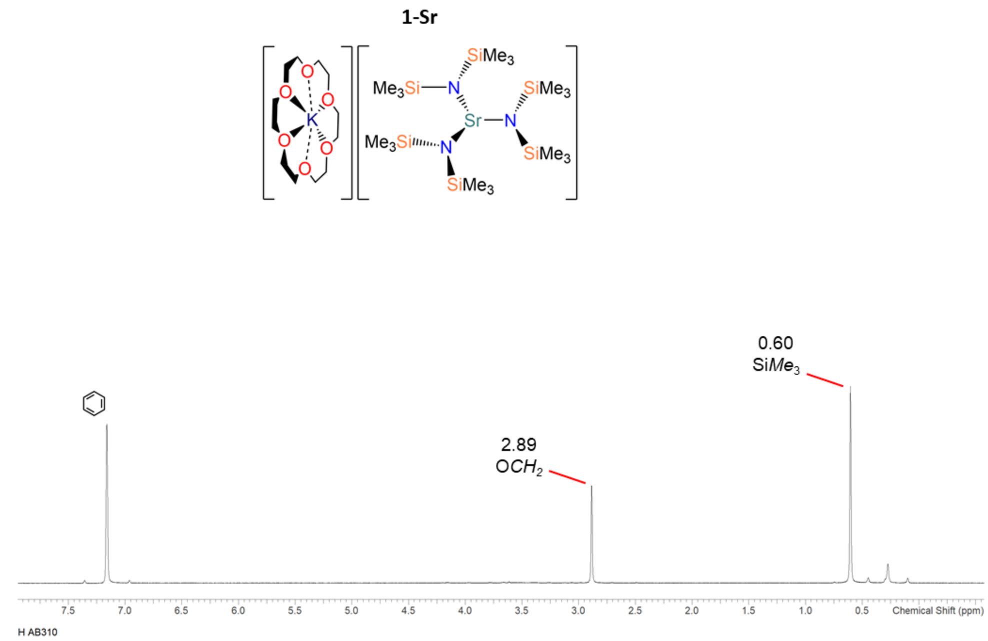

Figure S3: ^{1}H NMR (400 MHz, 298 K, C_6D_6) spectrum of **1-Sr**, with assignment.

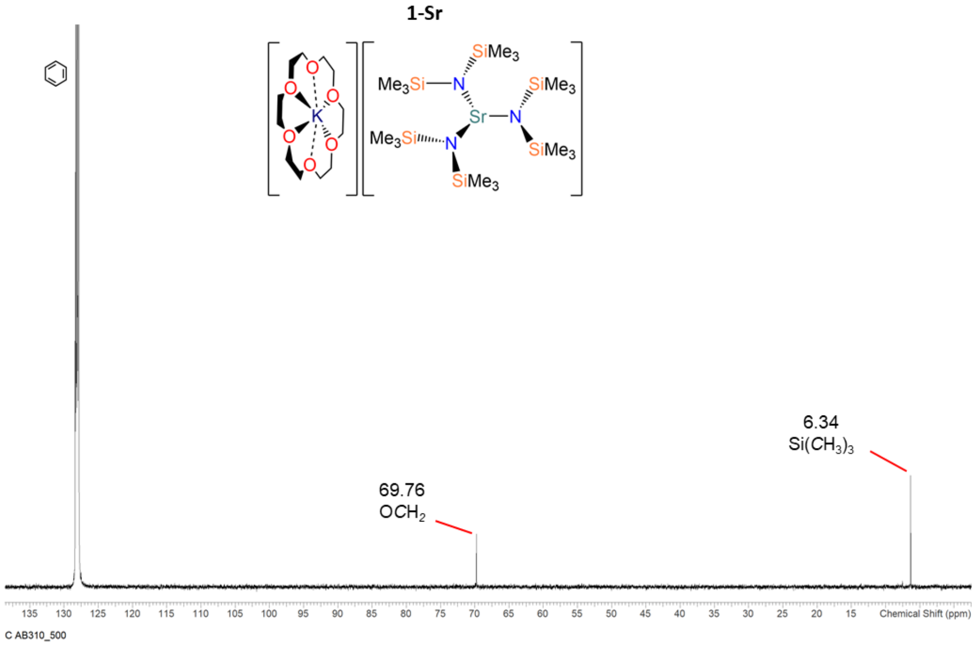

Figure S4: ^{13}C{^{1}H} NMR (125 MHz, 298 K, C_6D_6) spectrum of **1-Sr**, with assignment.

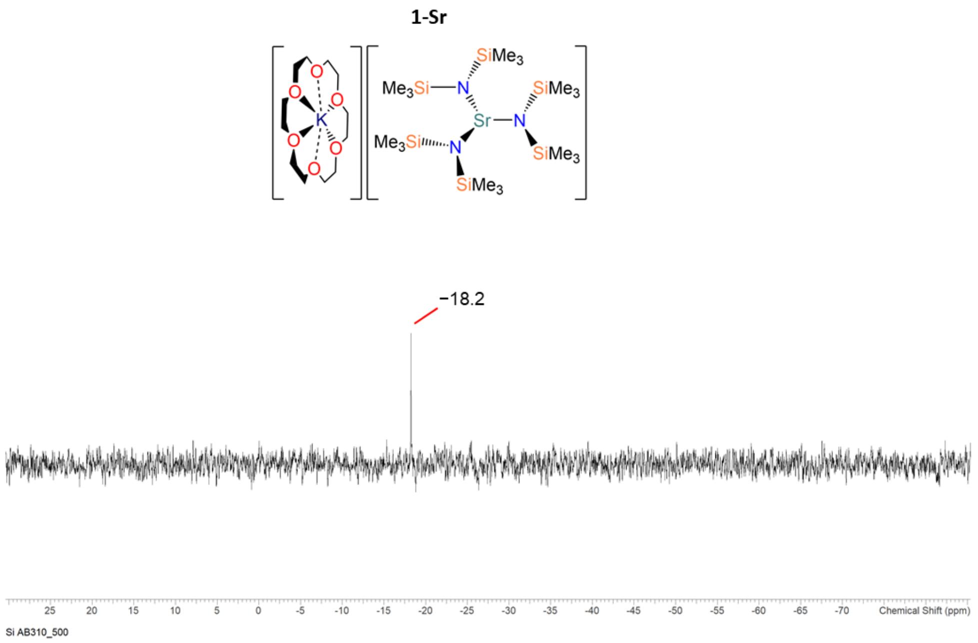

Figure S5: $^{29}Si\{^{1}H\}$ NMR (100 MHz, 298 K, C_6D_6) spectrum of **1-Sr**, with assignment.

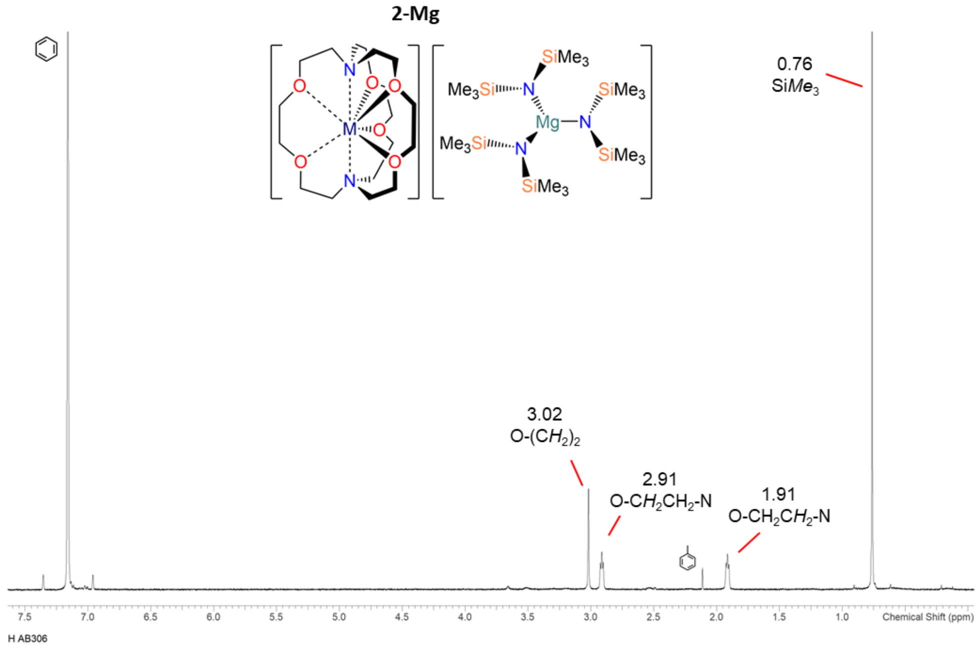

Figure S6: ^{1}H NMR (400 MHz, 298 K, C_6D_6) spectrum of **2-Mg**, with assignment.

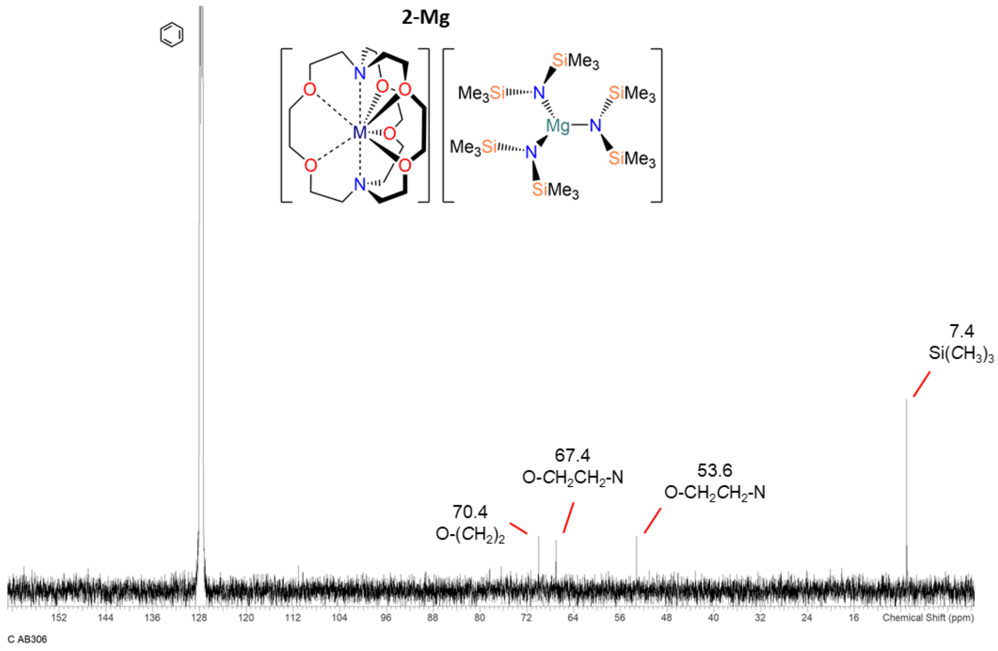

Figure S7: $^{13}C\{^{1}H\}$ NMR (100 MHz, 298 K, C_6D_6) spectrum of **2-Mg**, with assignment.

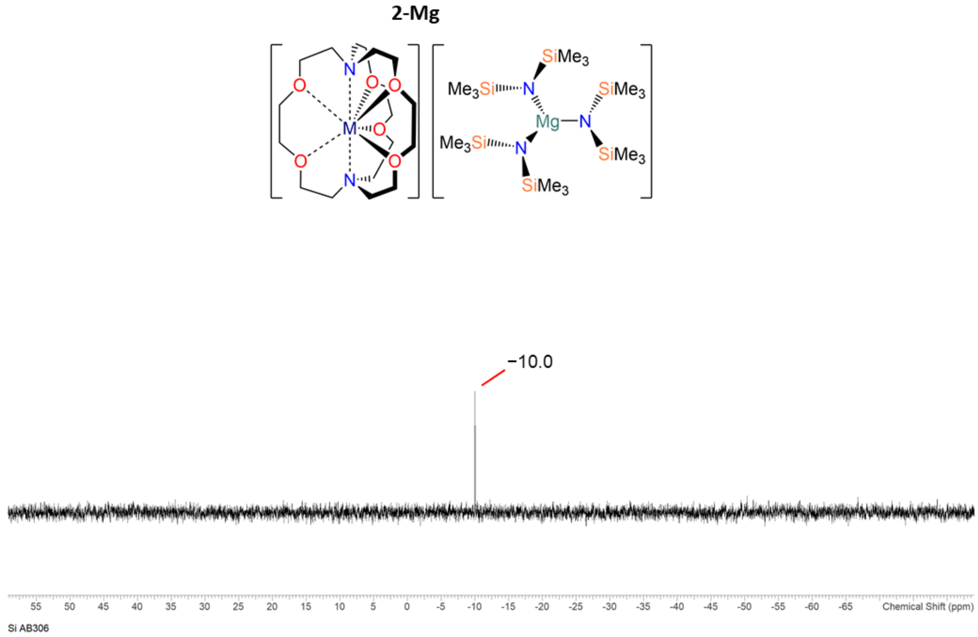

Figure S8: $^{29}Si\{^{1}H\}$ NMR (80 MHz, 298 K, C_6D_6) spectrum of **2-Mg**.

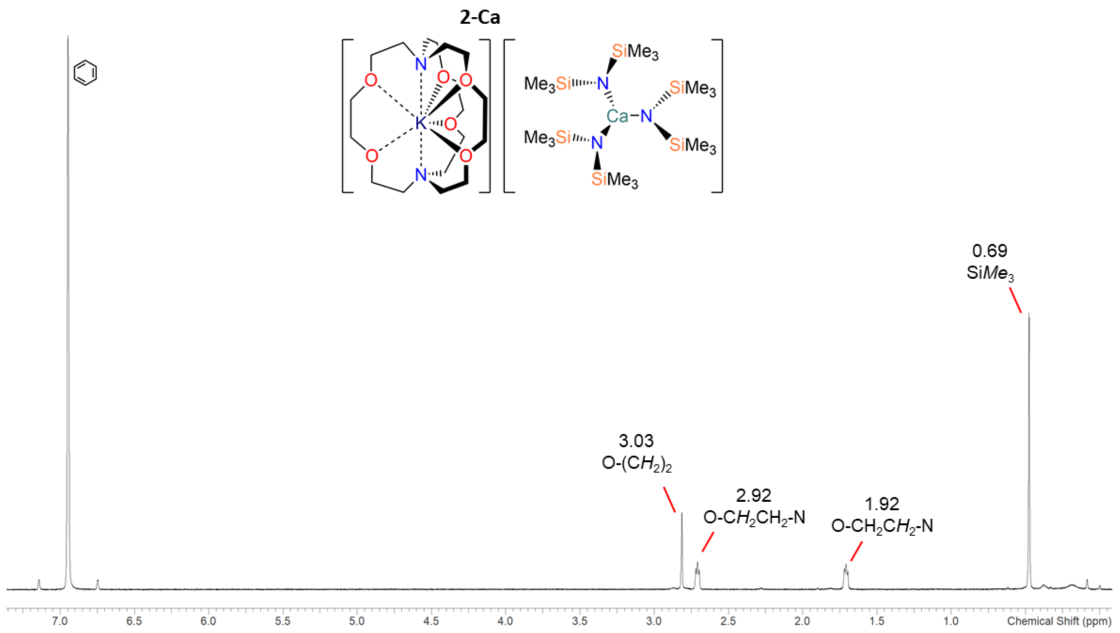

Figure S9: ^{1}H NMR (400 MHz, 298 K, C_6D_6) spectrum of **2-Ca**, with assignment.

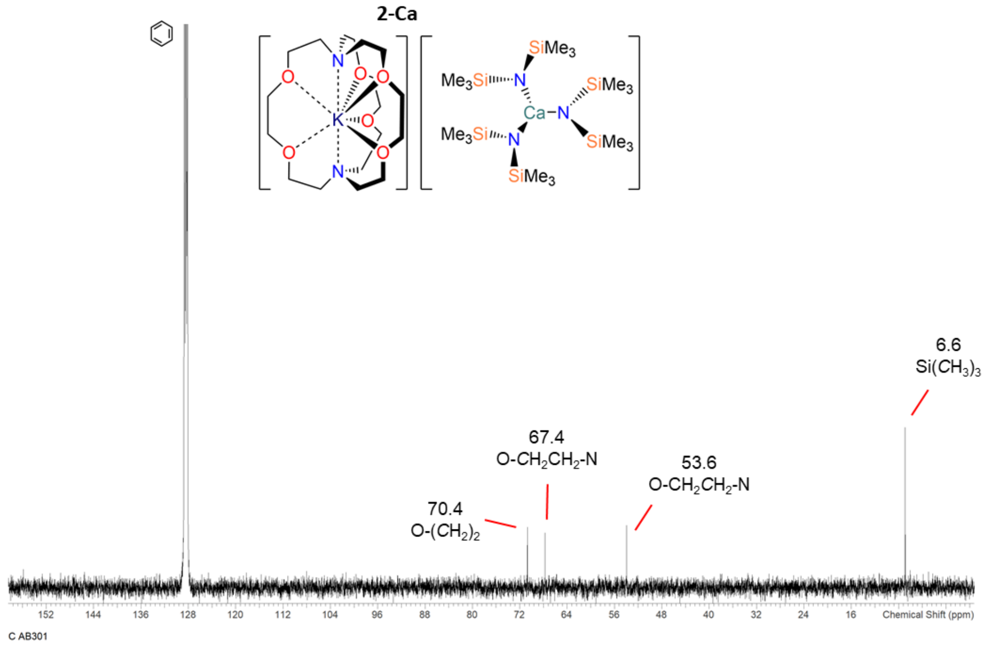

Figure S10: ^{13}C{^{1}H} NMR (100 MHz, 298 K, C_6D_6) spectrum of **2-Ca**, with assignment.

2-Ca

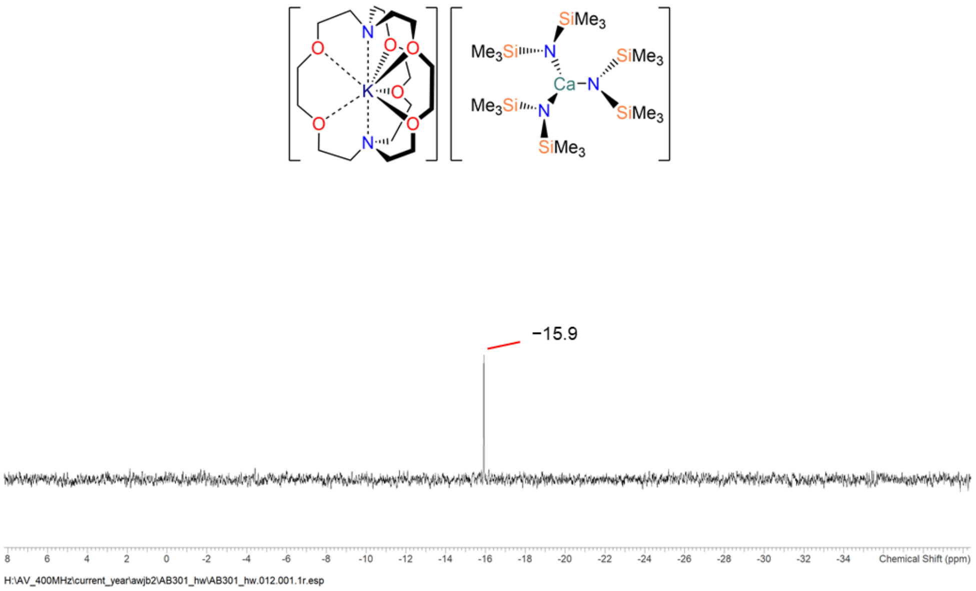

Figure S11: $^{29}Si\{^{1}H\}$ NMR (80 MHz, 298 K, C_6D_6) spectrum of **2-Ca**.

3-Mg

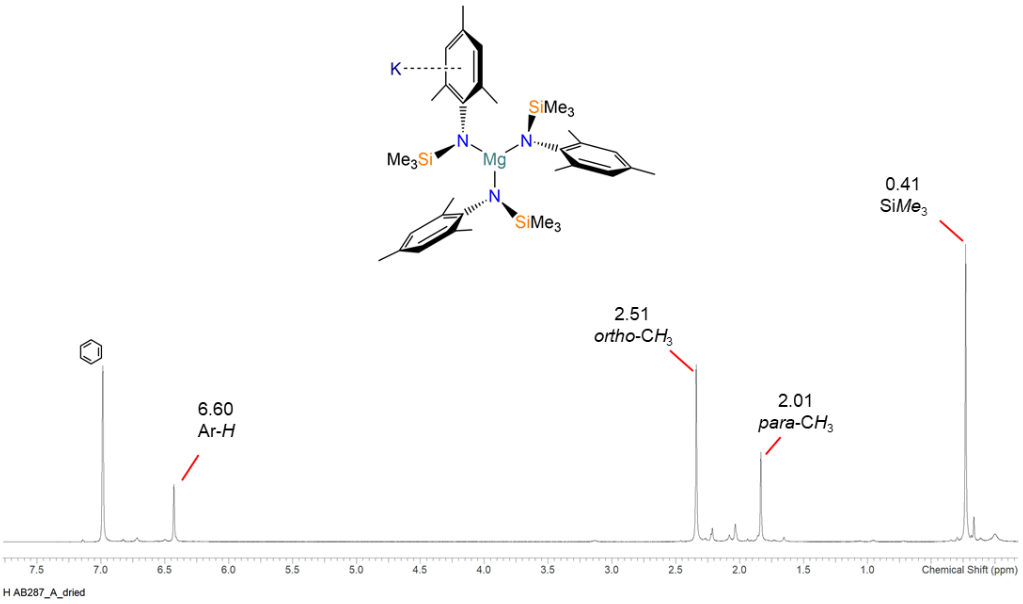

Figure S12: ^{1}H NMR (500 MHz, 298 K, C_6D_6) spectrum of **3-Mg**, with assignment.

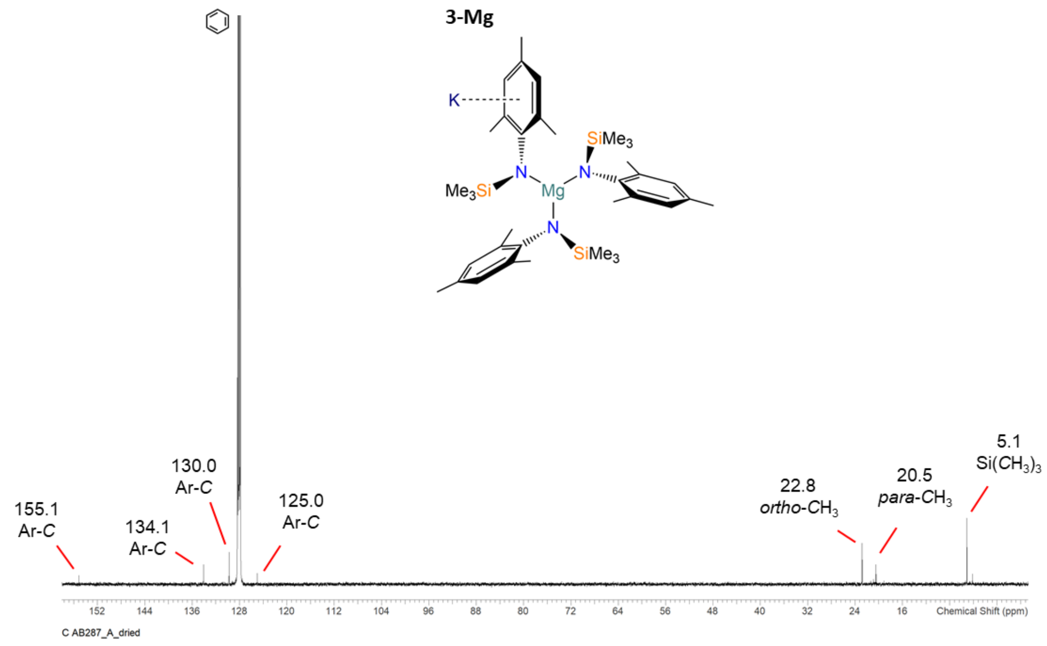

Figure S13: $^{13}C\{^1H\}$ NMR (125 MHz, 298 K, C_6D_6) spectrum of **3-Mg**, with assignment.

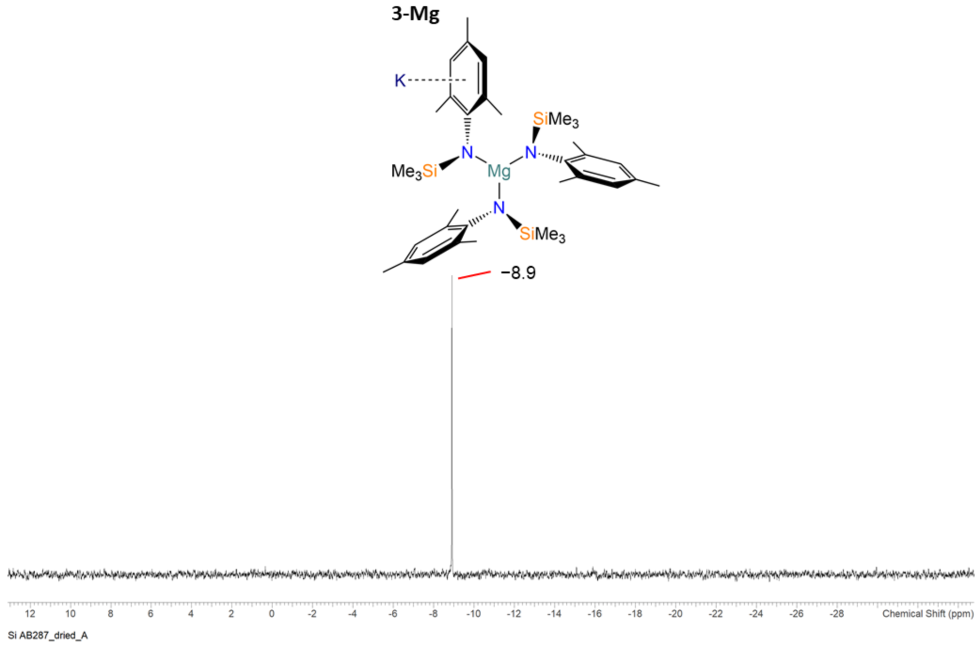

Figure S14: $^{29}Si\{^1H\}$ NMR (100 MHz, 298 K, C_6D_6) spectrum of **3-Mg**.

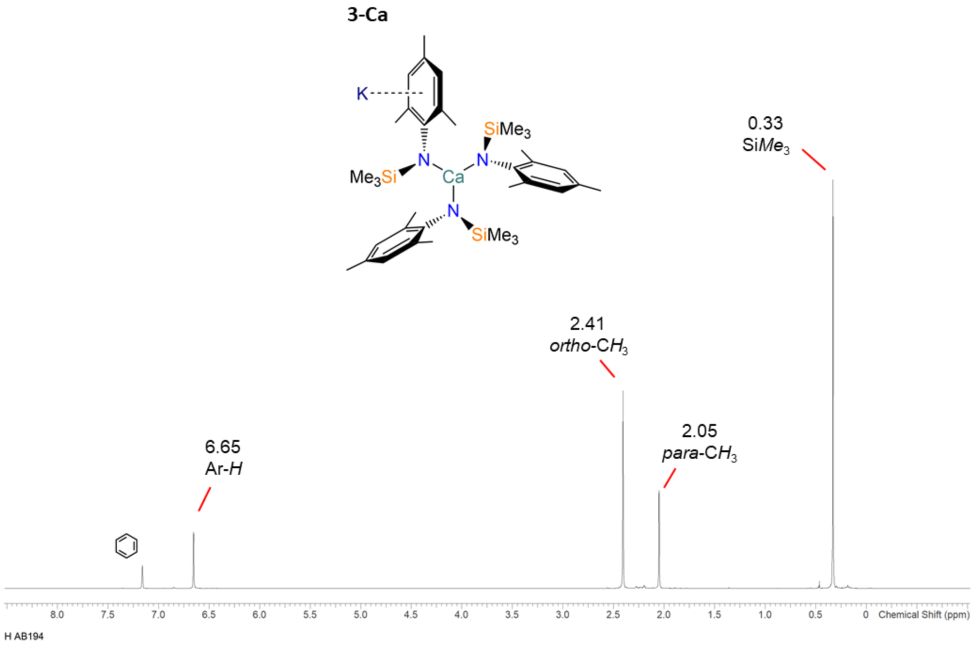

Figure S15: ^{1}H NMR (400 MHz, 298 K, C_6D_6) spectrum of **3-Ca**, with assignment.

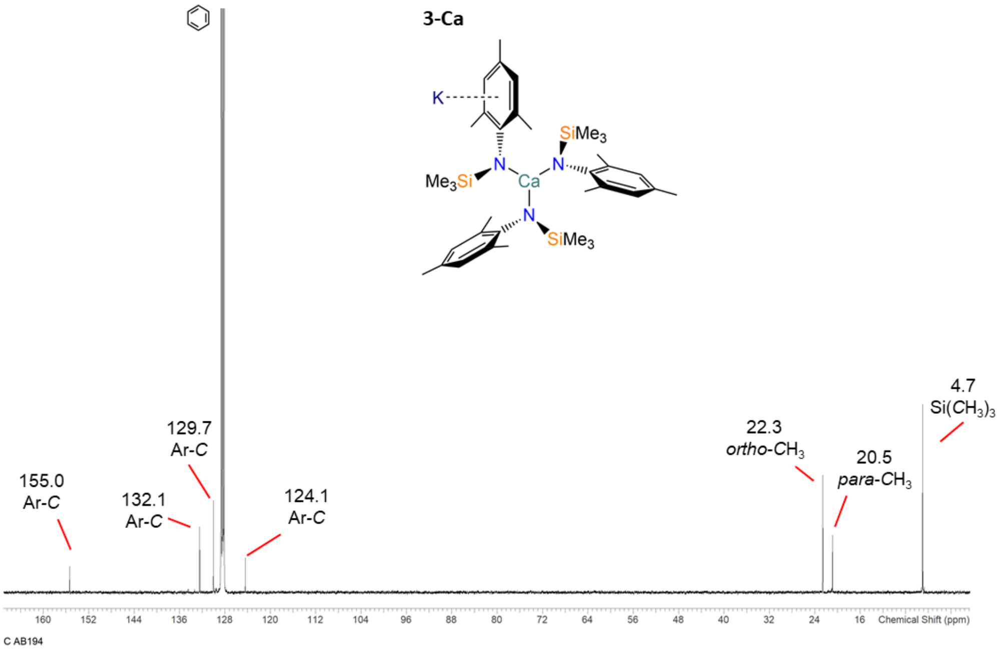

Figure S16: $^{13}C\{^{1}H\}$ NMR (100 MHz, 298 K, C_6D_6) spectrum of **3-Ca**, with assignment.

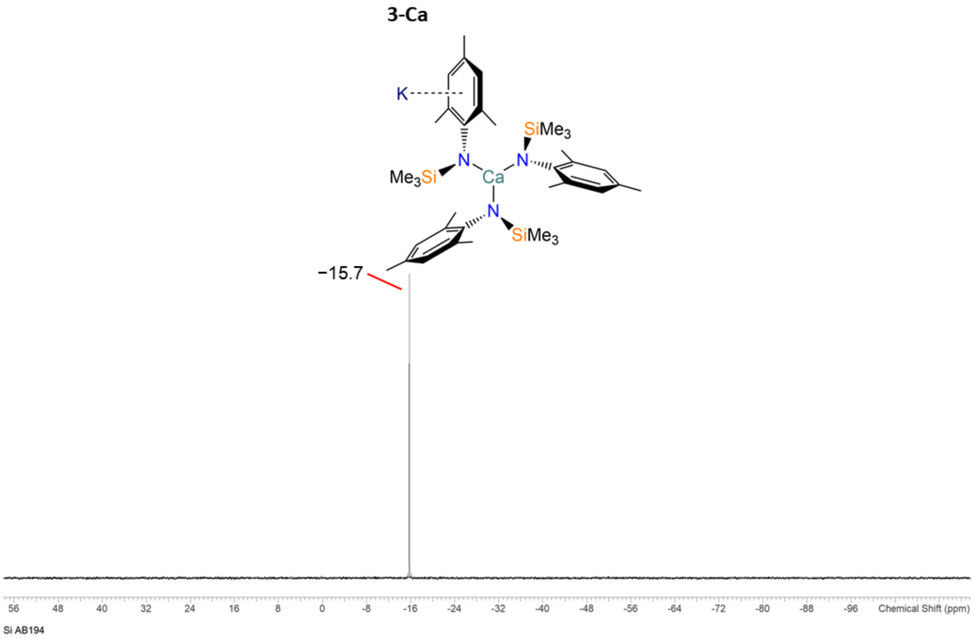

Figure S17: ^{29}Si{^{1}H} NMR (80 MHz, 298 K, C_6D_6) spectrum of **3-Ca**.

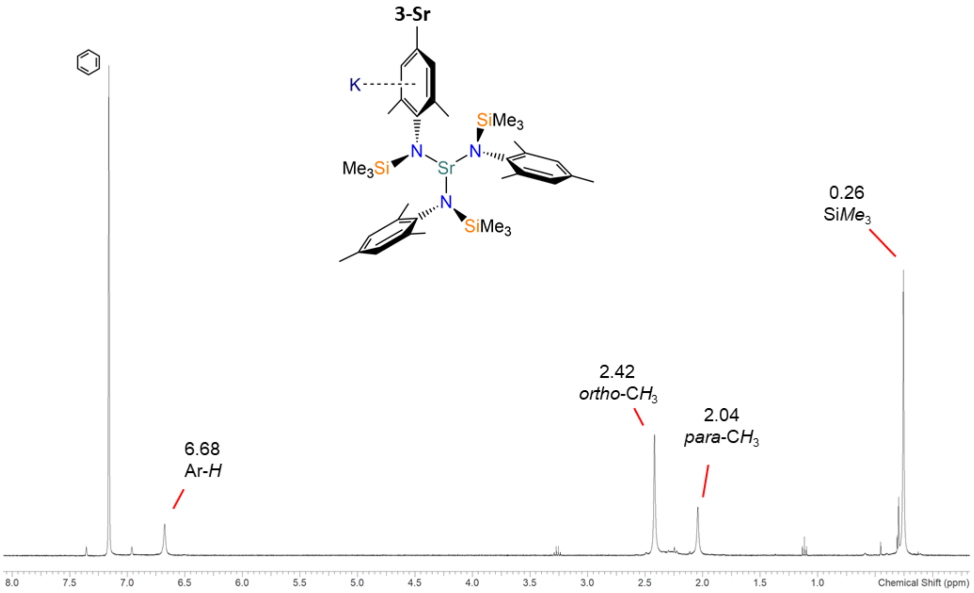

Figure S18: ^{1}H NMR (500 MHz, 298 K, C_6D_6) spectrum of **3-Sr**, with assignment.

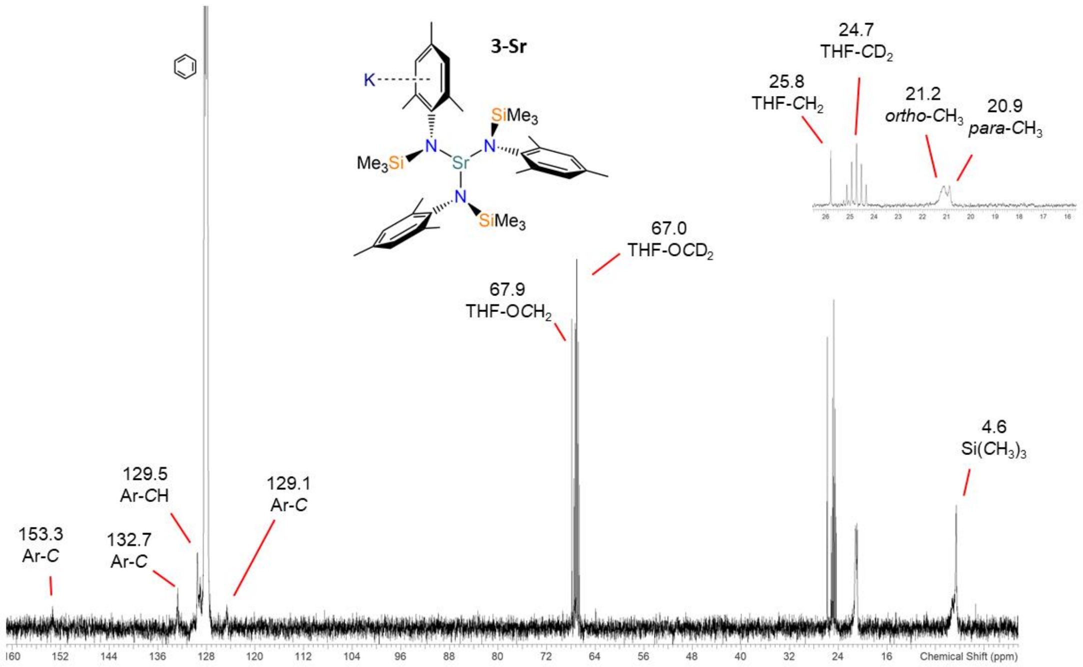

Figure S19: $^{13}C\{^{1}H\}$ NMR (100 MHz, 298 K, C_6D_6) spectrum of **3-Sr**, with assignment.

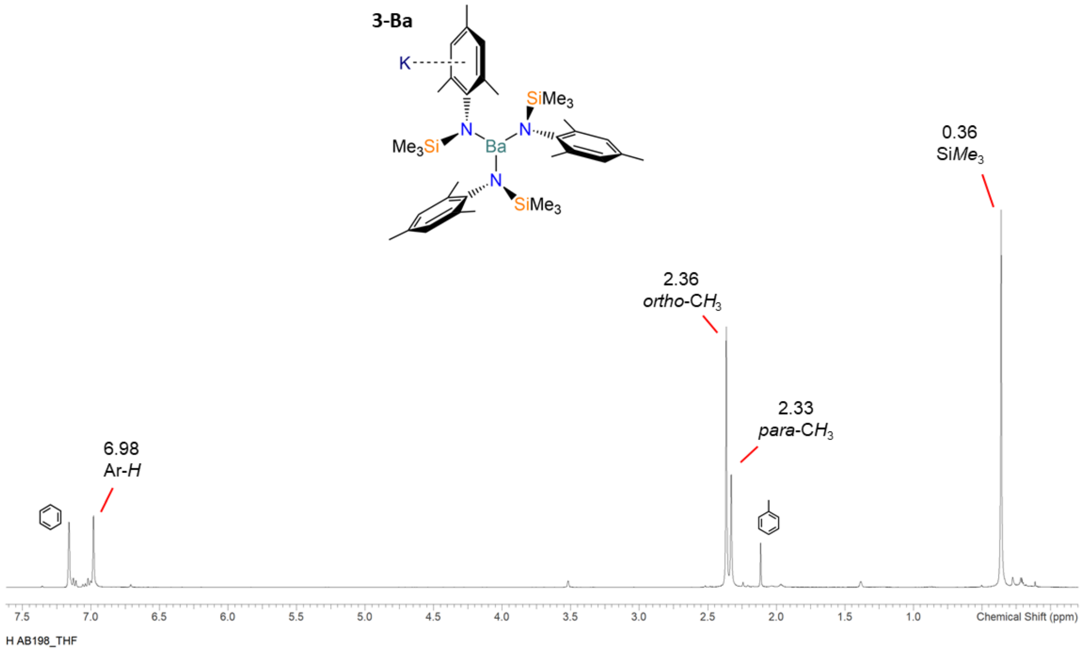

Figure S20: ^{1}H NMR (500 MHz, 298 K, C_6D_6) spectrum of **3-Ba**, with assignment.

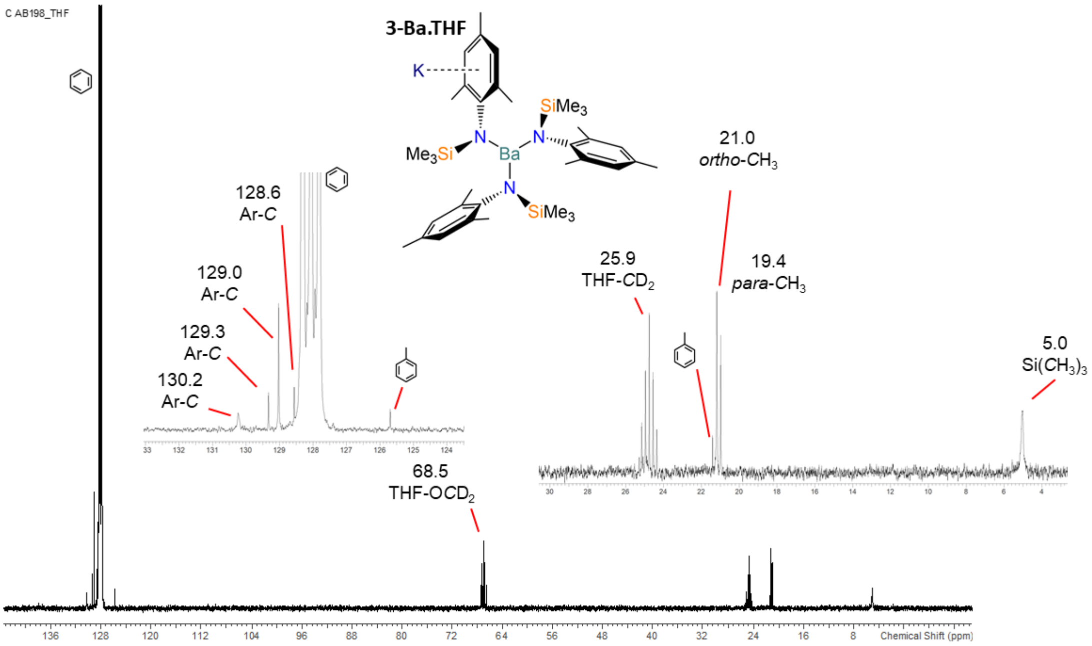

Figure S21: $^{13}C\{^{1}H\}$ NMR (125 MHz, 298 K, C_6D_6/C_4D_8O) spectrum of **3-Ba**, with assignment.

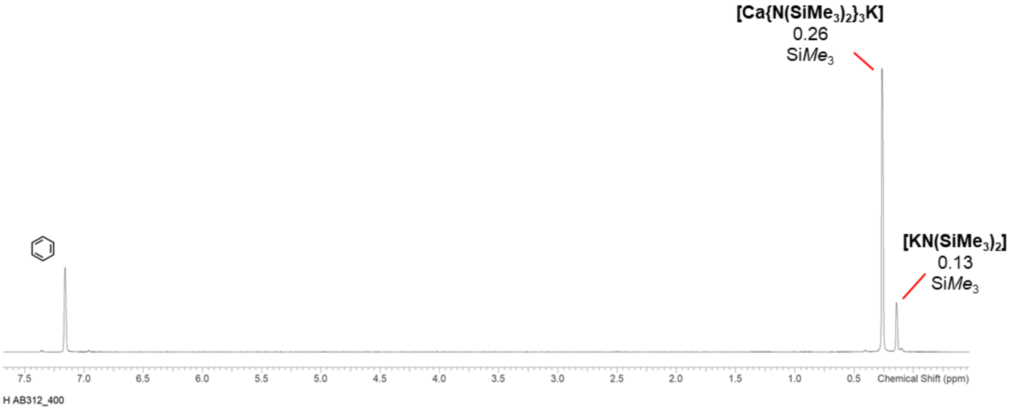

Figure S22: ^{1}H NMR (400 MHz, 298 K, C_6D_6) spectrum of the crude product mixture from the attempted reduction of $[Ca\{N(SiMe_3)_2\}_3K]$ *via* Method A.

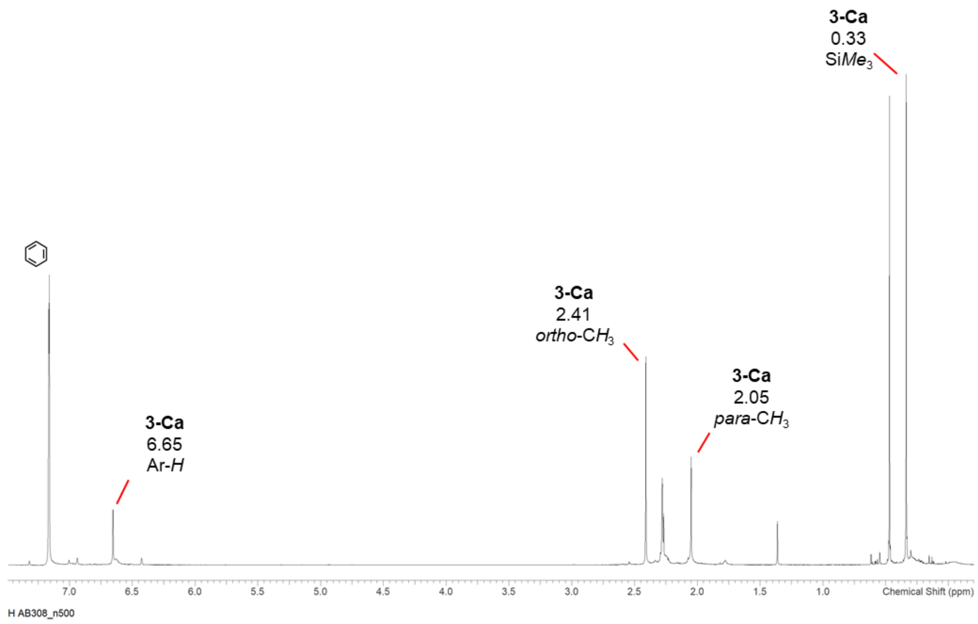

Figure S23: ^{1}H NMR (500 MHz, 298 K, C_6D_6) spectrum of the crude product mixture from the attempted reduction of **3-Ca** *via* Method A.

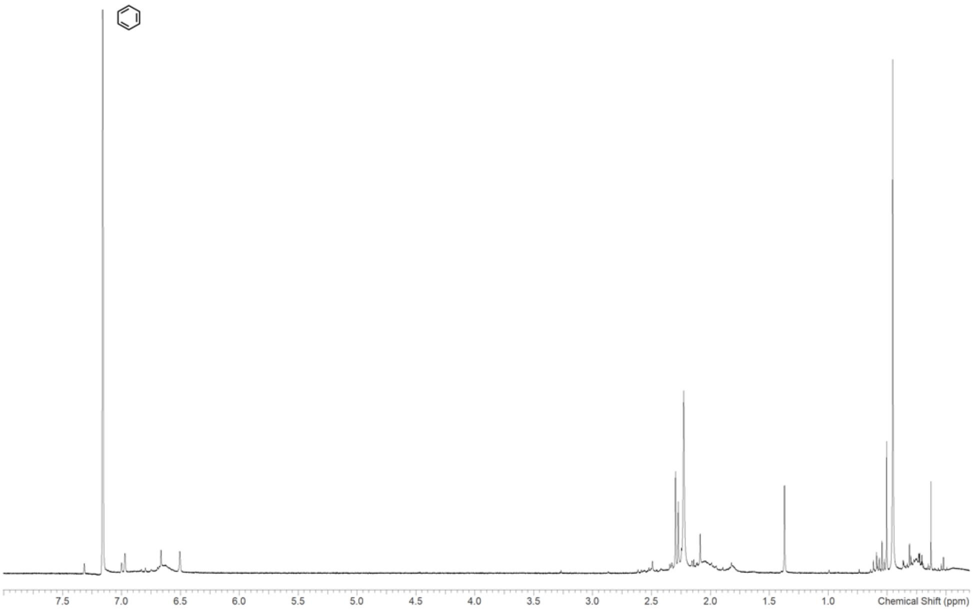

Figure S24: ^{1}H NMR (500 MHz, 298 K, C_6D_6) spectrum of the crude product mixture from the attempted reduction of **3-Sr** *via* Method A.

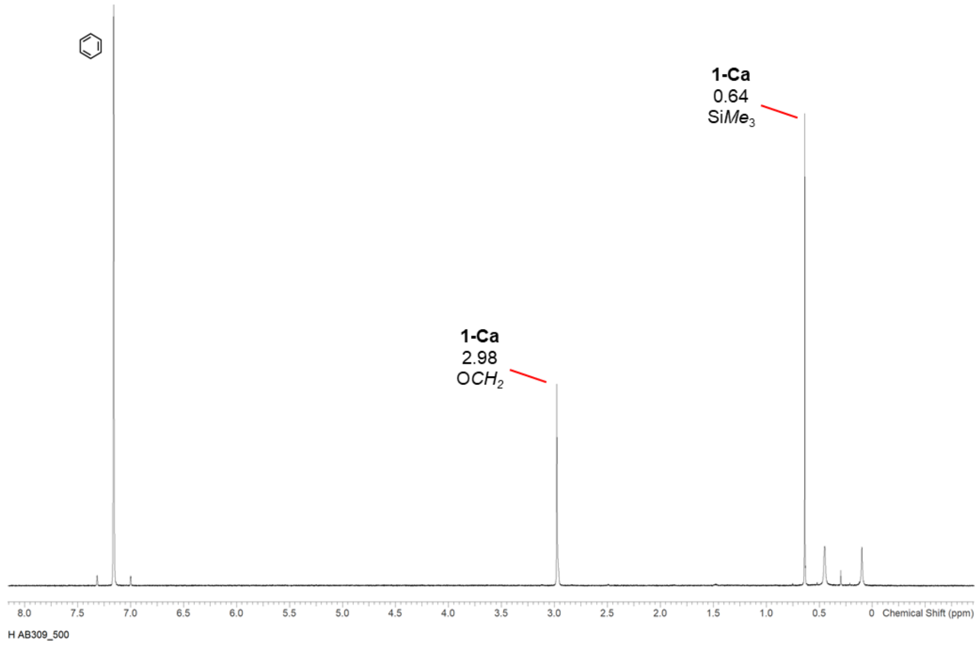

Figure S25: ^{1}H NMR (500 MHz, 298 K, C_6D_6) spectrum of the crude product mixture from the attempted reduction of [Ca{N(SiMe$_3$)$_2$}$_3$K] *via* Method B.

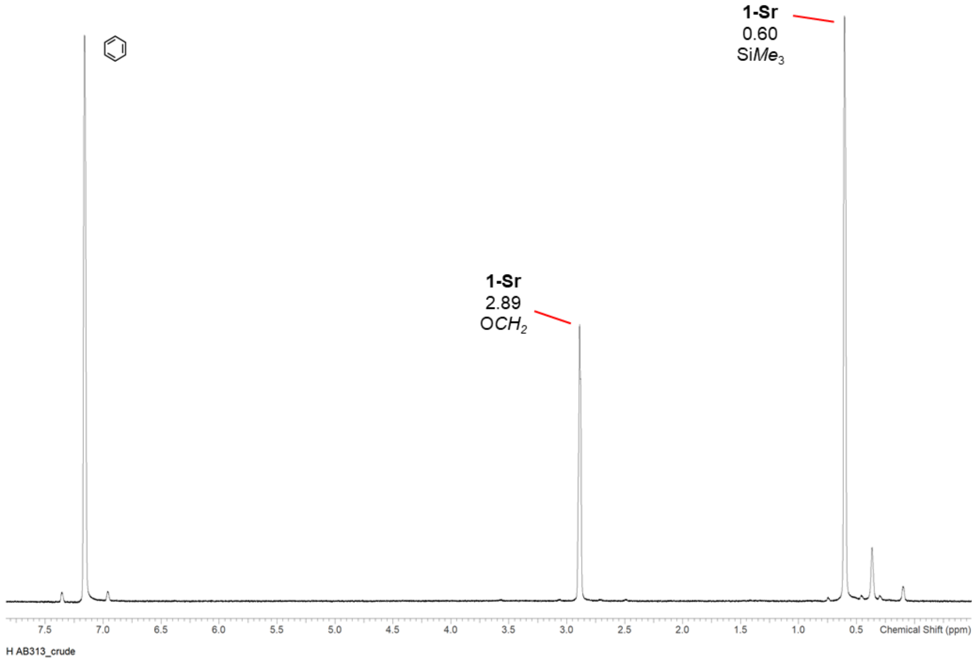

Figure S26: ^{1}H NMR (500 MHz, 298 K, C_6D_6) spectrum of the crude product mixture from the attempted reduction of [Sr{N(SiMe$_3$)$_2$}$_3$K] *via* Method B.

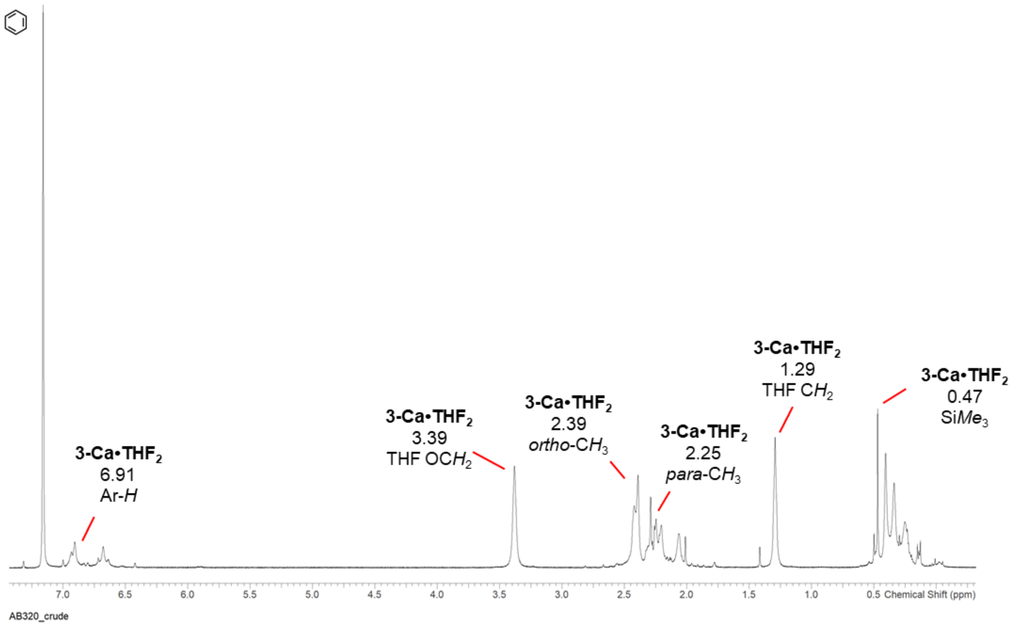

Figure S27: ^{1}H NMR (500 MHz, 298 K, C_6D_6) spectrum of the crude product mixture from the attempted reduction of **3-Ca** *via* Method B.

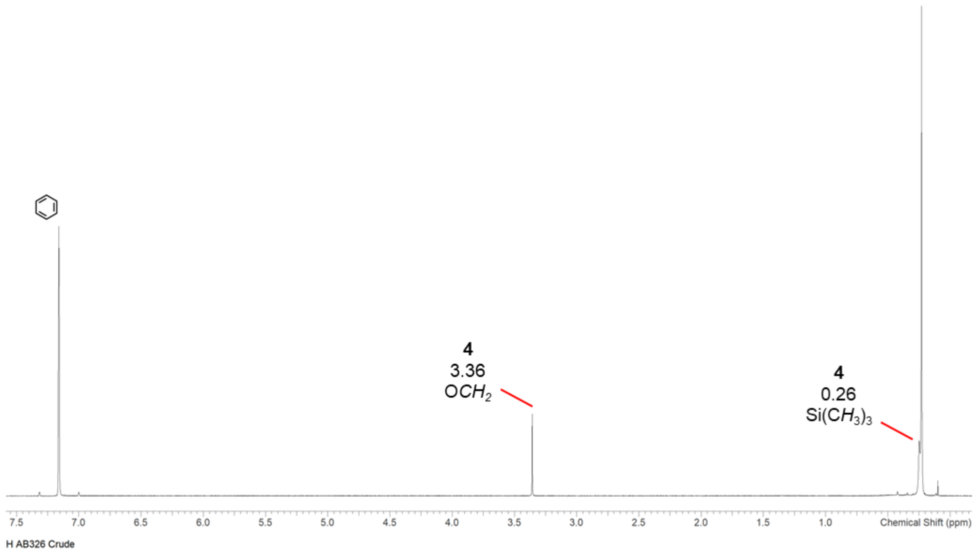

Figure S28: ^{1}H NMR (500 MHz, 298 K, C_6D_6) spectrum of the crude product mixture from the attempted reduction of **4** *via* Method C.

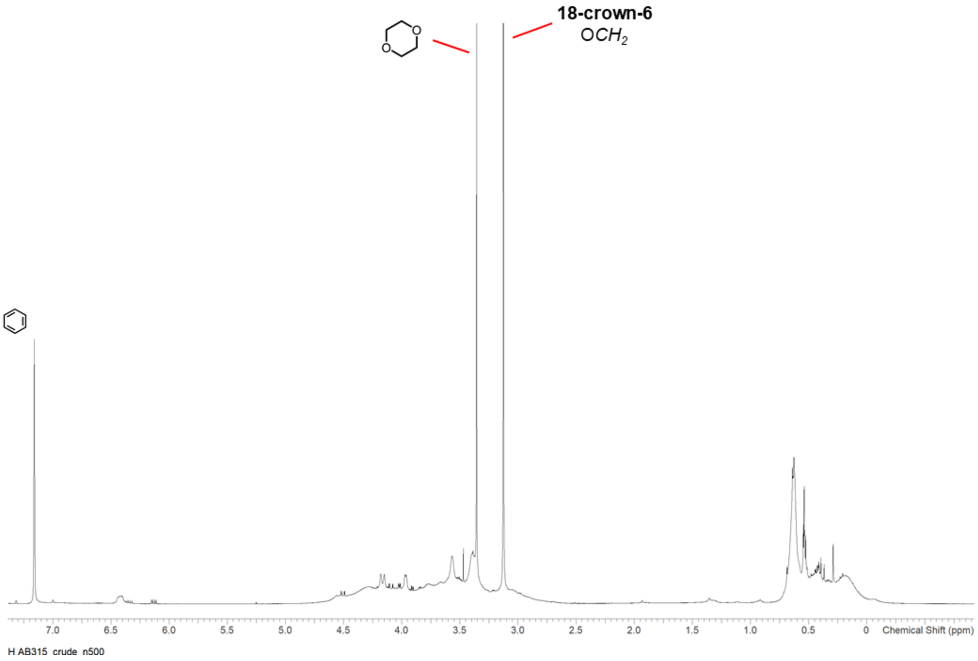

Figure S29: ^{1}H NMR (500 MHz, 298 K, C_6D_6) spectrum of the crude product mixture from the attempted reduction of **4** *via* Method D.

S3. IR data

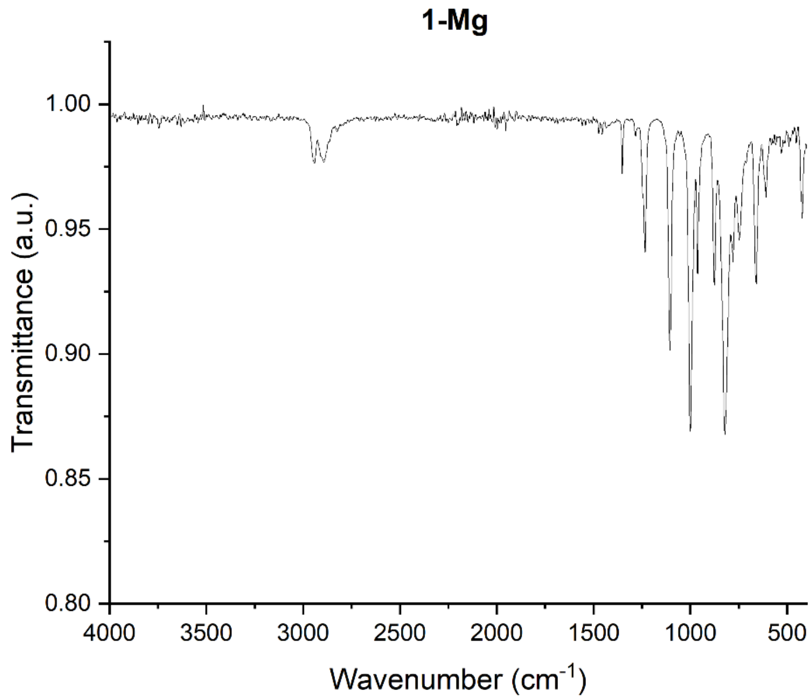

Figure S30: FTIR spectrum of **1-Mg**.

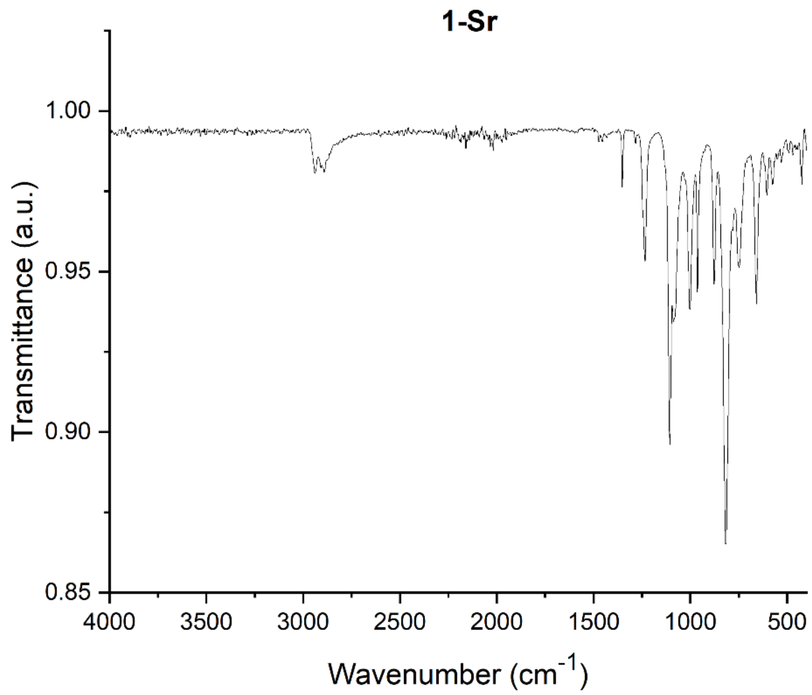

Figure S31: FTIR spectrum of **1-Sr**.

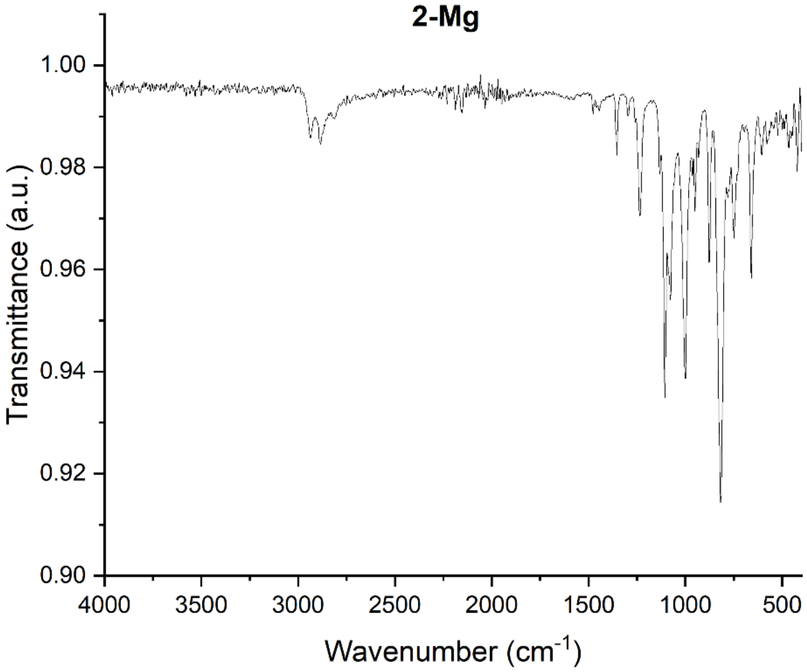

Figure S32: FTIR spectrum of **2-Mg**.

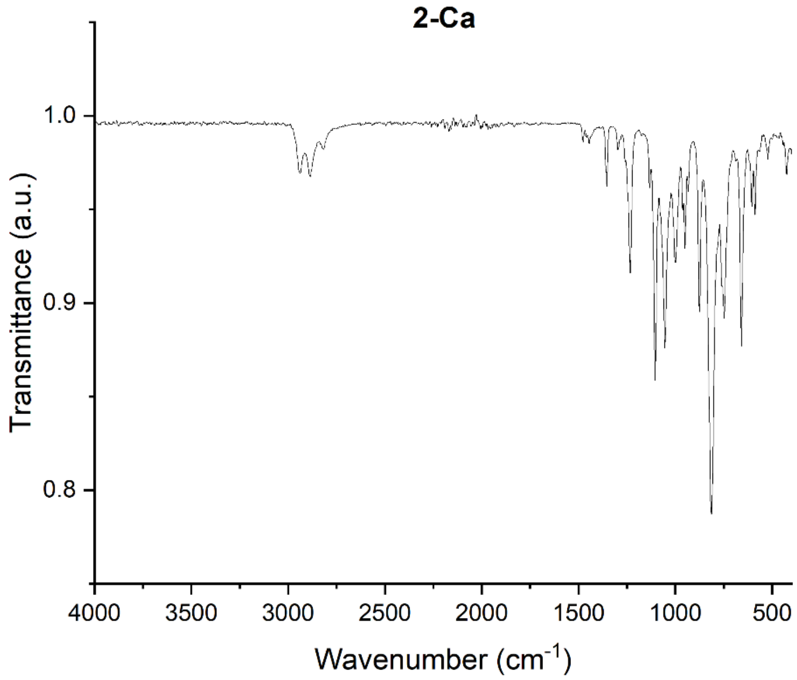

Figure S33: FTIR spectrum of **2-Ca**.

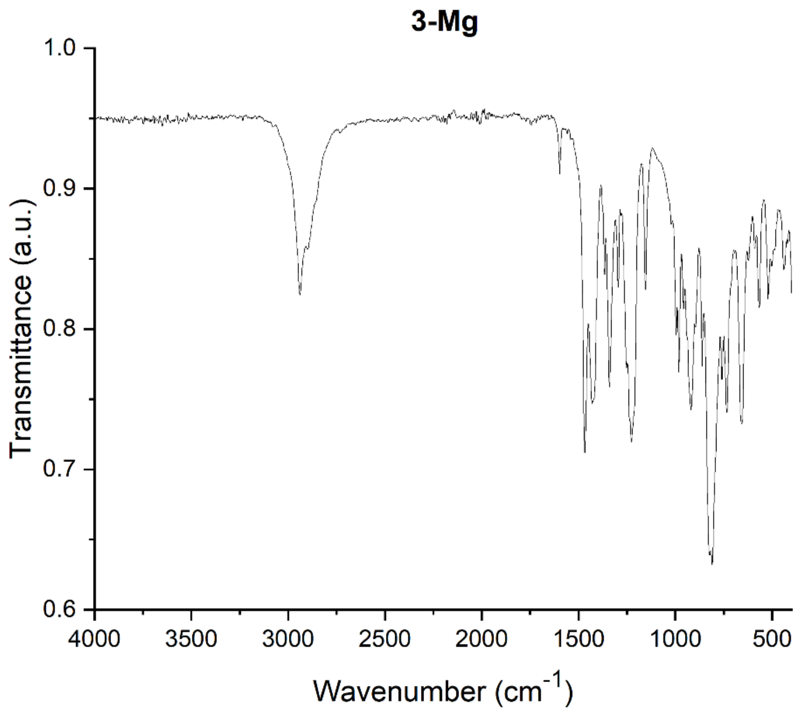

Figure S34: FTIR spectrum of **3-Mg**.

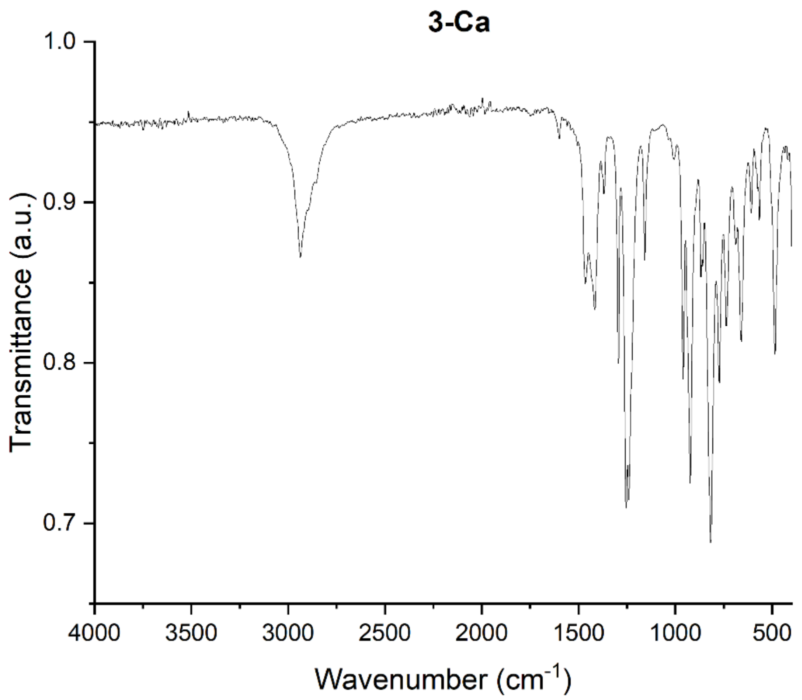

Figure S35: FTIR spectrum of **3-Ca**.

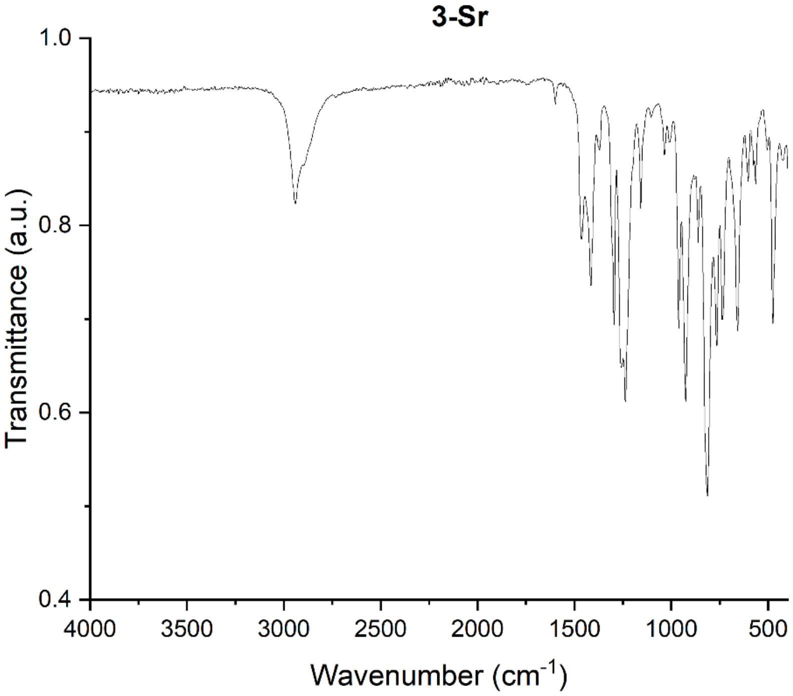

Figure S36: FTIR spectrum of **3-Sr**.

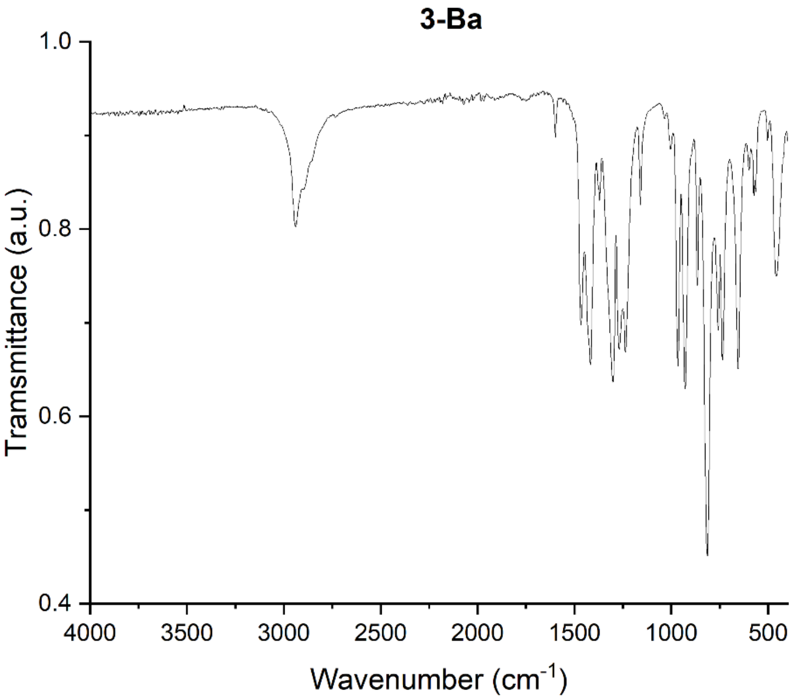

Figure S37: FTIR spectrum of **3-Ba**.

S4. Crystallography

Crystallographic method

The crystal data for all compounds are compiled in Tables S1–S3. Crystals of **1-Mg**, **2-Ca**, **3-AE** (AE = Mg-Sr), **3-Ca·(THF)$_2$** and **3-Ba·(THF)** were examined using a Bruker D8 Quest diffractometer with a Photon III detector and a microfocus source with Cu-Kα radiation (λ = 1.54178). Crystals of **1-Sr** and **2-Mg** were examined using a Rigaku FR-X diffractometer with a Hypix6000HE detector and a graphite-monochromated Cu-Kα (λ = 1.54178 Å). Intensities were integrated from data recorded on 1° frames by ω or φ rotation. A multi-scan method (**1-Mg**, **2-Ca**, **3-Mg**, **3-Ca**, **3-Sr**, **3-Ca·(THF)$_2$**, **3-Ba·(THF)**)[9] or a Gaussian grid face-indexed (**1-Sr**, **2-Mg**) absorption correction with a beam profile was applied.[10] The structures were solved using SHELXS[11] or SHELXT;[12] the datasets were refined by full-matrix least-squares on reflections with $F^2 \geq 2\sigma(F^2)$ values, with anisotropic displacement parameters for all non-hydrogen atoms, and with constrained riding hydrogen geometries;[11] U_{iso}(H) was set at 1.2 (1.5 for methyl groups) times U_{eq} of the parent atom. The largest features in final difference syntheses were close to heavy atoms and were of no chemical significance. SHELX[11,12] was employed through OLEX2 for structure solution and refinement.[13] ORTEP-3[14] and POV-Ray[15] were employed for molecular graphics. The structures have been deposited with the Cambridge Crystallographic Data Centre (CCDC 2259496-2259504 and 2263802). This information can be obtained free of charge from www.ccdc.cam.ac.uk/data_request/cif.

Table S1: Crystallographic data for **1-AE**

	**1-Mg**	**1-Sr**	**[Sr{N(SiMe$_3$)$_2$}$_3$K]**
Formula	$C_{30}H_{78}KMgN_3O_8Si_6$	$C_{44}H_{94}KN_3O_6Si_6Sr$	$C_{18}H_{54}KN_3Si_6Sr$
Formula Weight	808.90	1056.48	607.90
Crystal Size, mm	0.01 x 0.01 x 0.05	0.17 x 0.22 x 0.29	0.24 x 0.25 x 0.32
Crystal System	Triclinic	Monoclinic	Monoclinic
Space group	$P2_1$	$P2_1/c$	$C2/c$
a, Å	11.0264(2)	12.56613(13)	8.5992(3)
b, Å	18.5096(3)	31.2722(3)	21.2587(6)
c, Å	12.1616(3)	15.74180(15)	19.1835(5)
α, °	90	90	90
β, °	102.937(2)	93.7098(9)	90.549(1)
γ, °	90	90	90
V, Å^3	2419(9)	6173.10(11)	3506.7(2)
Z	2	4	4
Temperature, K	120(2)	150(2)	120(2)
ρ_{calc}, g cm^{-3}	1.110	1.137	1.151
μ, mm^{-1}	2.804	3.245	5.235
F(*000*)	884	2272	1296
No. of reflections (unique)	31421 (9956)	30772 (12274)	20127 (3477)
S^a	1.05	1.04	1.12
$R_1(wR_2)$ ($F^2 > 2\sigma(F^2)$)	0.0511 (0.1252)	0.0367 (0.0959)	0.0338 (0.0951)
R_{int}	0.134	0.035	0.047
Min./max. diff map, Å^{-3}	-0.43, 0.33	-0.56, 0.41	-0.71, 0.98

[a]Conventional $R = \Sigma||Fo| - |Fc||/\Sigma|Fo|$; $Rw = [\Sigma w(Fo^2 - Fc^2)^2/\Sigma w(Fo^2)^2]^{1/2}$; $S = [\Sigma w(Fo^2 - Fc^2)^2/\text{no. data} - \text{no. params})]^{1/2}$ for all data.

Table S2: Crystallographic data for **2-AE**

	**2-Mg**	**2-Ca**
Formula	$C_{39.5}H_{92.5}KMgN_5O_6Si_6$	$C_{39}H_{93}CaKN_5O_6Si_6$
Formula Weight	965.63	975.90
Crystal Size, mm	0.328 x 0.223 x 0.177	0.01 x 0.01 x 0.02
Crystal System	Triclinic	Triclinic
Space group	*P*-1	*P*-1
a, Å	11.6299(3)	11.7276(7)
b, Å	15.3653(4)	15.5177(9)
c, Å	16.6889(5)	16.8542(10)
α, °	94.734(2)	95.499(4)
β, °	102.963(3)	104.976(3)
γ, °	90.489(2)	90.915(3)
V, Å^3	2895.21(15)	2946.8(3)
Z	2	2
Temperature, K	150(2)	120(2)
ρ_{calc}, g cm^{-3}	1.108	1.110
μ, mm^{-1}	2.427	3.039
F(*000*)	1055.0	1066
No. of reflections (unique)	25430 (11345)	61573 (11576)
S[a]	1.05	1.00
$R_1(wR_2)$ ($F^2 > 2\sigma(F^2)$)	0.0346 (0.0972)	0.0514 (0.1481)
R_{int}	0.023	0.107
Min./max. diff map, Å^{-3}	-0.46, 0.64	-0.38, 0.75

[a]Conventional $R = \Sigma||Fo| - |Fc||/\Sigma|Fo|$; $Rw = [\Sigma w(Fo^2 - Fc^2)^2/\Sigma w(Fo^2)^2]^{1/2}$; $S = [\Sigma w(Fo^2 - Fc^2)^2$/no. data – no. params)]$^{1/2}$ for all data.

Table S3: Crystallographic data for **3-AE**

	**3-Mg**	**3-Ca**	**3-Ca·(THF)$_2$**	**3-Sr**	**3-Ba·(THF)**
Formula	$C_{158}H_{256}K_4Mg_4N_{12}Si_{12}$	$C_{36}H_{60}CaKN_3Si_3$	$C_{44}H_{76}CaKN_3O_2Si_3$	$C_{36}H_{60}KN_3Si_3Sr$	$C_{40}H_{68}BaKN_3OSi_3$, 0.667[C_7H_8]
Formula Weight	2914.45	698.32	842.54	745.869	929.14
Crystal Size, mm	0.16 x 0.22 x 0.23	0.907 x 0.383 x 0.264	0.052 x 0.105 x 0.277	0.165 x 0.104 x 0.085	0.018 x 0.090 x 0.533
Crystal System	Monoclinic	Monoclinic	Rhombohedral	Monoclinic	Monoclinic
Space group	$P2_1/n$	$P2_1/c$	R-3	$P2_1/n$	Cc
a, Å	12.0254(3)	12.5861(4)	51.8155(10)	11.4492(5)	24.5535(8)
b, Å	19.8480(5)	17.8902(7)	51.8155(10)	16.8687(9)	34.7586(10)
c, Å	18.0130(4)	18.2553(7)	11.6427(3)	20.8549(10)	21.2795(6)
α, °	90	90	90	90	90
β, °	93.709(1)	94.2848(12)	90	95.784(3)	125.395(1)
γ, °	90	90	120	90	90
V, Å^3	4290.3(2)	4099.0(3)	27071.0(3)	4007.3(3)	14804.4(8)
Z	1	4	18	4	12
Temperature, K	120(2)	120(2)	120(2)	120(2)	120(2)
ρ_{calc}, g cm^{-3}	1.128	1.132	1.073	1.236	1.168
μ, mm^{-1}	2.241	3.260	2.365	3.853	7.960
F(*000*)	1580	1512.0	9594	1590.8	5448
No. of reflections (unique)	72342 (8483)	56164 (8100)	134088(11806)	26450 (7405)	85091 (24458)
S^a	1.08	1.06	1.02	1.04	1.03
$R_1(wR_2)$ ($F^2 > 2\sigma(F^2)$)	0.0662 (0.1860)	0.0301 (0.0826)	0.0500(0.1272)	0.0759 (0.2195)	0.0481 (0.1251)
R_{int}	0.062	0.030	0.142	0.099	0.061
Min./max. diff map, Å^{-3}	-0.80, 0.71	-0.30, 0.37	-0.28, 0.30	-1.32, 1.64	-0.74, 1.09

^{a}Conventional $R = \Sigma||Fo| - |Fc||/\Sigma|Fo|$; $Rw = [\Sigma w(Fo^2 - Fc^2)^2/\Sigma w(Fo^2)^2]^{1/2}$; $S = [\Sigma w(Fo^2 - Fc^2)^2/\text{no. data} - \text{no. params})]^{1/2}$ for all data.

Table S4: Relevant bond lengths (Å) and angles (°) of **1-AE** and **2-AE** and **3-AE**.

	**1-Mg**	**1-Ca**	**1-Sr**	**2-Mg**	**2-Ca**
AE–N	2.023(3)-<br>2.033(3)	2.3103(10)-<br>2.3121(10)	2.457(2)-<br>2.465(2)	2.0189(13)-<br>2.0323(14)	2.305(2)-<br>2.315(3)
N–AE–N	119.54(15)-<br>120.52(14)	119.00(4)-<br>120.51(4)	118.81(6)-<br>121.12(7)	119.01(6)-<br>121.74(6)	116.99(9)-<br>123.61(9)
AE···N^N^N	0.008(3)	0.1189(6)	0.090(2)	0.0045(8)	0.0095(13)

Table S5: Relevant bond lengths (Å) and angles (°) of **3-AE**.

	**3-Mg**	**3-Ca**	**3-Ca·(THF)$_2$**	**3-Sr**	**3-Ba·(THF)**
AE–N	2.049(3)-<br>2.090(3)	2.3192(10)-<br>2.3465(11)	2.3733(18)-<br>2.4295(18)	2.468(5)-<br>2.496(4)	2.617(1)-<br>2.717(9)
N–AE–N	116.62(10)-<br>125.83(10)	112.92(4)-<br>122.59(4)	114.45(6)-<br>125.93(6)	114.22(15)-<br>123.49(15)	109.1(3)-<br>117.7(2)
AE···N^N^N	0.052(2)	0.1400(7)	0.3210(11)	0.138(3)	0.692(5)-<br>0.696(4)

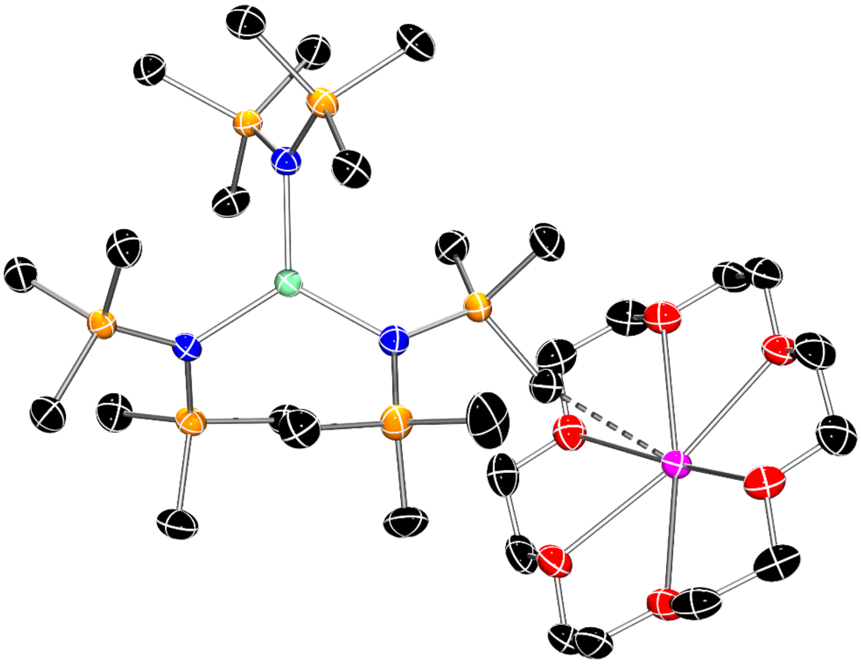

Figure S38: Crystal structure of **1-Mg**. Ellipsoids are set at 50% probability level. Hydrogen atoms have been omitted for clarity.

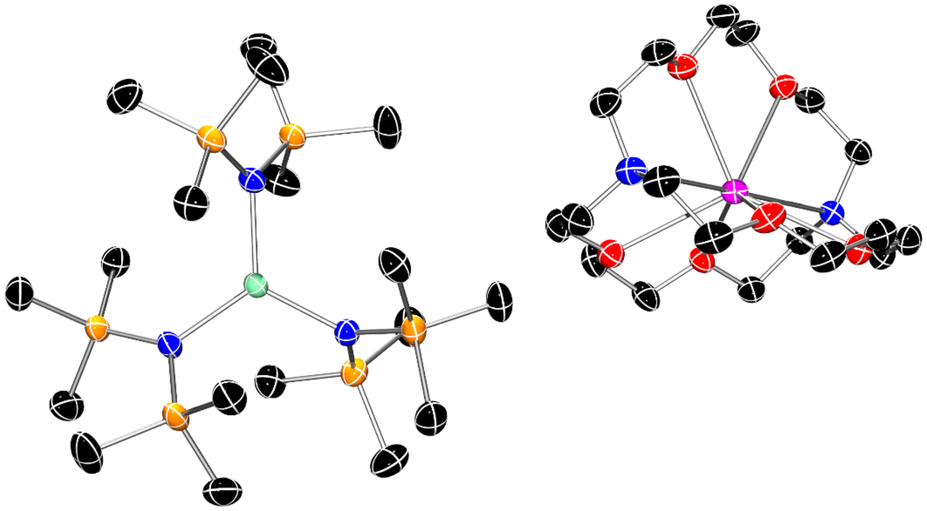

Figure S39: Crystal structure of **2-Mg**. Ellipsoids are set at 50% probability level. Hydrogen atoms have been omitted for clarity.

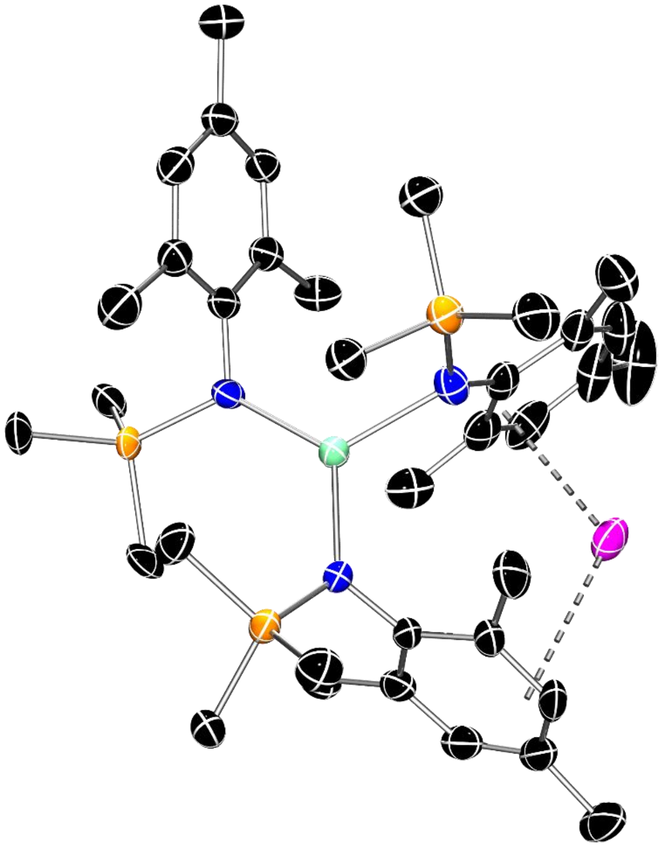

Figure S40: Crystal structure of **3-Mg**. Ellipsoids are set at 50% probability level. Hydrogen atoms have been omitted for clarity.

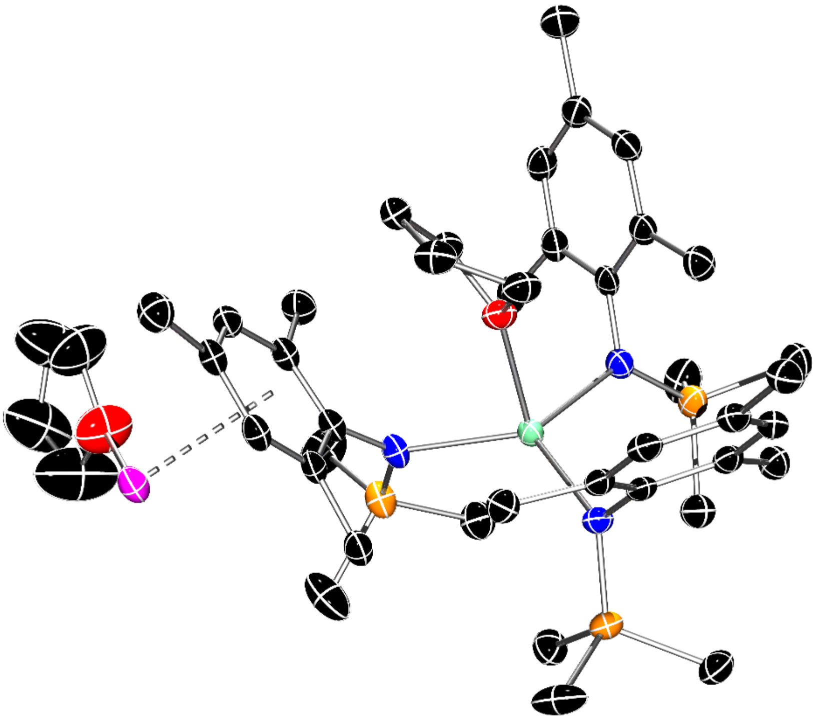

Figure S41: Crystal structure of **3-Ca·(THF)$_2$**. Ellipsoids are set at 50% probability level. Hydrogen atoms have been omitted for clarity.

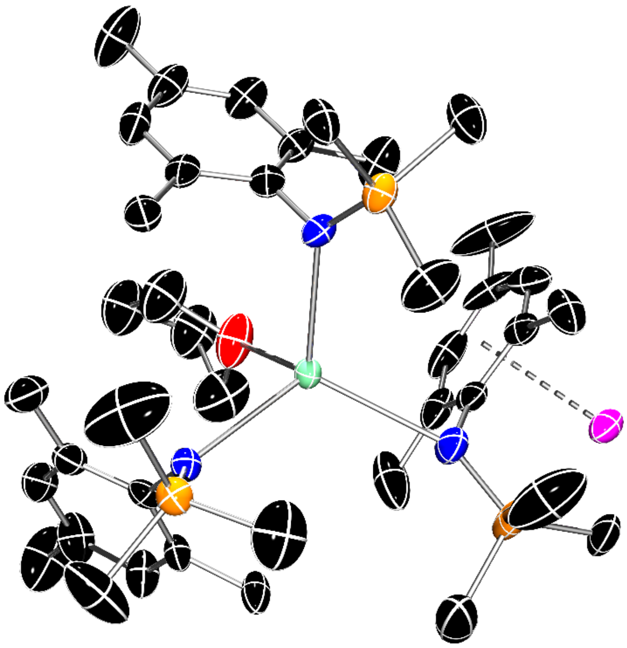

Figure S42: Crystal structure of **3-Ba·(THF)**. Ellipsoids are set at 50% probability level. Hydrogen atoms have been omitted for clarity.

S5. Computational details

Table S6 – Number of basis functions for each AE atom in the basis sets used for DFT calculations. Number of primitive functions given in ellipses.

**Atom**	**BS1**	**BS2**
Mg	8 (16)	30 (50)
Ca	26 (54)	43 (197)
Sr	26 (54)	33 (58)
Ba	33 (64)	40 (98)

Breakdown of Energy Contributions

The following list details the evolution of the relative energies as the successive corrections to the initial SCF energy are included. Terms used are:

$\mathbf{\Delta E_{BSI}}$	SCF energy computed with the BP86 functional with BS1
$\mathbf{\Delta H_{BSI}}$	Enthalpy at 0 K with BS1
$\mathbf{\Delta G_{BSI}}$	Free energy at 298.15 K and 1 atm with BS1
$\mathbf{\Delta G_{BSI/\mathit{THF}}}$	Free energy corrected for THF solvent with BS1
$\mathbf{\Delta G_{BSI/\mathit{THF}+D3BJ}}$	Free energy corrected for THF and dispersion effects with BS1
$\mathbf{\Delta E_{BS2}}$	SCF energy computed with the BP86 functional with BS2
$\mathbf{\Delta G_{THF}}$	Free energy corrected for basis set (BS2), dispersion effects and THF solvent

Table S7 – Relative energies for computed structures in kcal/mol. Data in bold are those used in the main text. Free energies of neutral potassium ion pairs (**IP**) and dianionic doublets ($^{2-}$) are quoted relative to the anionic singlet species.

Complex	AE	Species	ΔE_{BSI}	ΔH_{BSI}	ΔG_{BSI}	$\Delta G_{BSI/THF}$	$\Delta G_{BSI/THF+D3BJ}$	ΔE_{BS2}	ΔG_{THF}
$^{2}[AE\{N(SiMe_3)_2\}_3]^{2-}$	**Mg**		86.5	83.9	83.5	4.7	7.8	64.2	**−14.5**
	**Ca**		75.2	73.7	74.3	−4.0	−3.1	57.8	**−20.5**
	**Sr**		68.9	68.0	69.6	−5.9	−6.5	55.2	**−20.2**
	**Ba**		61.9	61.3	64.1	−7.9	−10.5	50.3	**−22.1**
$[AE\{N(SiMe_3)(Mes)\}_3]^{-}$	**Mg**	**IP**	−96.6	−94.9	−85.4	0.1	−6.4	−96.0	**−5.8**
		$^{2-}$	78.3	71.9	71.4	−1.8	−1.1	46.5	**−32.9**
		$^{2-}$*	77.4	72.9	72.9	−0.9	0.7	59.9	**−16.8**
	**Ca**	**IP**	−79.4	−78.6	−69.8	4.5	−2.7	−79.6	**−3.0**
		$^{2-}$	70.3	66.0	65.5	−6.9	−5.2	51.6	**−23.9**
	**Sr**	**IP**	−77.2	−76.1	−67.3	4.5	−1.9	−77.8	**−2.6**
		$^{2-}$	62.3	60.3	60.0	−12.1	−11.3	52.0	**−21.6**
	**Ba**	**IP**	−78.8	−77.6	−69.3	3.7	−3.0	−79.1	**−3.4**
		$^{2-}$	60.3	59.6	60.7	−9.1	−7.0	49.4	**−17.9**
	**Ba**	**With THF** **IP**	−89.7	−87.4	−64.8	11.5	−11.8	−87.2	**−9.4**
		**With THF** $^{1-}$	−9.9	−9.0	3.7	7.5	−8.7	−7.0	**−5.7**
		**With THF** $^{2-}$	55.7	55.1	68.7	1.3	−14.1	45.3	**−24.5**
$[Sr\{N(SiMe_3)_2\}_2(dioxane)_2]^{-}$			4.6	3.6	4.2	−20.7	−19.1	−2.4	**−26.1**

* conformation of ligand based on **3-Ca** crystal structure not **3-Mg** crystal structure

Deviation of AE from the Ligand Plane

Using Mercury, the deviation of the AE atom out of the ligand plane (i.e. NNN or NONO) was measured by first selecting the donor ligand atoms at AE to generate a plane (shown as blue in the figure below). The distance between the AE centre and the plane was then calculated by Mercury and are reported in the table below.

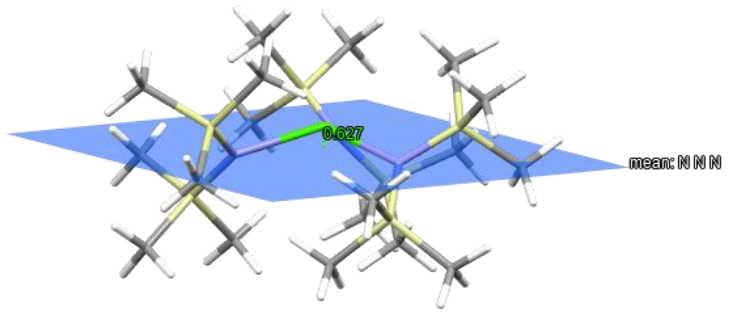

Figure S43: **2-Ca**; NNN plane (blue) with the distance between the NNN plane and Ca shown as 0.627 Å

Table S8 – Distance between the AE atom and the ligand donor atom plane [in Å]

		**n = 1**	**n = 2**
**[AE{N(SiMe$_3$)$_2$)$_3$}]$^{n-}$**		AE–{NNN}	
**1-Mg**	Mg	0.000	0.594
**1-Ca**	Ca	0.001	0.627
**1-Sr**	Sr	0.000	0.773
**1-Ba**	Ba	0.003	0.874
**[AE{N(SiMe$_3$)(Mes)}$_3$]$^{n-}$**		AE–{NNN}	
**3-Mg**	Mg	0.008	0.013
	Mg*	0.009	0.398
**3-Ca**	Ca	0.040	0.378
**3-Sr**	Sr	0.154	0.646
**3-Ba·(THF)**	Ba (no THF)	0.558	0.736
**3-Ba**	Ba	0.561	0.251
**[Sr{N(SiMe$_3$)$_2$}$_2$(dioxane)$_2$]**		AE–{NONO}	
**4-Sr**	Sr	0.056	0.565

* conformation of ligand based on **3-Ca** crystal structure not **3-Mg** crystal structure

NBO Contributions % (*s* | *p* | *d*) to specific Molecular Orbitals for Monoanion (LUMO+*n*) and Dianion (SOMO)

2-AE

[AE{N($SiMe_3$)$_2$}$_3$]

	Monoanion [AE{N($SiMe_3$)$_2$}$_3$]$^-$			Dianion [AE{N($SiMe_3$)$_2$}$_3$]$^{2-}$		
**1-Mg**	LUMO = 141<br>0.298*[141]: LV (1)Mg73(lv)<br>0.247*[862]: RY (13)Mg73(ry)<br>-0.242*[864]: RY (15)Mg73(ry)<br>-0.230*[865]: RY (16)Mg73(ry)			SOMO = 141<br>0.802*[57]: LP (1)Mg73(lp)		
	**NBO 141 = LV of Mg**			**NBO 57 = LP of Mg**		
	**99.76 %**	**0.00 %**	**0.24 %**	**84.24 %**	**15.69 %**	**0.07 %**
**1-Ca**	LUMO+6 = 151<br>0.384*[856]: RY (3)Ca73(ry)<br>0.334*[145]: LV (1)Ca73(lv)			SOMO = 145<br>0.679*[61]: LP (1)Ca73(lp)		
	**NBO 145 = LV of Ca**			**NBO 61 = LP of Ca**		
	**97.68 %**	**0.00 %**	**2.08 %**	**94.36 %**	**2.47 %**	**3.10 %**
**1-Sr**	LUMO+6 = 146<br>0.365*[140]: LV (1)Sr82(lv)<br>0.309*[***]: RY (3)Sr82(ry)			SOMO = 140<br>-0.747*[62]: LP (1)Sr82(lp)		
	**NBO 140 = LV of Sr**			**NBO 62 = LP of Sr**		
	**98.73 %**	**0.00 %**	**1.27 %**	**90.49 %**	**5.29 %**	**4.22 %**
**1-Ba**	LUMO+6 = 146<br>0.353*[***]: RY (3)Ba82(ry)<br>-0.320*[140]: LV (1)Ba82(lv)<br>-0.314*[***]: RY (10)Ba82(ry)			SOMO = 140<br>-0.874*[62]: LP (1)Ba82(lp)		
	**NBO 140 = LV of Ba**			**NBO 62 = LP of Ba**		
	**98.02 %**	**0.00 %**	**1.43 %**	**65.25 %**	**30.98 %**	**3.76 %**

3-AE

$[AE\{N(Mes)(SiMe_3)\}_3]$

	Monoanion $[AE\{N(Mes)(SiMe_3)\}_3]^{-}$	Dianion $[AE\{N(Mes)(SiMe_3)\}_3]^{2-}$
**3-Mg**	LUMO = 177<br>-0.240*[343]: RY (15)Mg 3(ry)<br><br>NBO 343 is a very high NBO, hence the large contributions from the *p* and *d* orbital types, which we caution putting any weight or meaning to.	SOMO = 177<br>-0.404*[329]: RY (1)Mg 3(ry)<br>0.394*[894]: RY (1) H54(ry)<br>0.382*[***]: RY (1) H86(ry)<br>0.263*[882]: RY (1) H52(ry)<br>0.242*[***]: RY (1) H85(ry)
	**NBO 343 = RY of Mg**<br>**41.20 %** | **45.39 %** | **13.41 %**	**NBO 329 = RY of Mg**<br>**65.06 %** | **34.85 %** | **0.10 %**

	Monoanion	Dianion
**3-Ca**	LUMO = 181<br>0.422*[181]: LV (1)Ca 1(lv)<br>-0.348*[258]: BD*(2) C62- C91*<br>0.249*[281]: BD*(2) C89- C90*	SOMO = 181<br>-0.416*[543]: RY (1) H19(ry)<br>-0.326*[182]: LV (1)Ca 1(lv)<br>-0.307*[259]: BD*(2) C62- C91*<br>-0.294*[531]: RY (1) H17(ry)<br>-0.275*[70]: LP (1) C89(lp)<br>-0.265*[537]: RY (1) H18(ry)
	**NBO 181 = LV of Ca**<br>**97.45 %** | **0.14 %** | **2.25 %**	**NBO 182 = LV of Ca**<br>**92.43 %** | **0.62 %** | **6.82 %**

	Monoanion	Dianion
**3-Sr**	LUMO = 176<br>-0.487*[176]: LV (1)Sr 1(lv)<br>0.392*[205]: BD*(2) C21- C22*	SOMO = 176<br>-0.747*[59]: LP (1)Sr 1(lp)<br>-0.285*[***]: RY (1) H66(ry)<br>-0.255*[***]: RY (1) H67(ry)
	**NBO 176 = LV of Sr**<br>**99.22 %** | **0.01 %** | **0.77 %**	**NBO 59 = LP of Sr**<br>**83.76 %** | **8.94 %** | **7.30 %**

**3-Ba**·THF	LUMO = 196			SOMO = 196		
	-0.400*[196]: LV (1)Ba 1(lv)			-0.673*[64]: LP (1)Ba 1(lp)		
				-0.296*[***]: RY (1) H87(ry)		
				-0.251*[***]: RY (1) H86(ry)		
				-0.229*[***]: RY (1) H88(ry)		
	**NBO 186 = LV of Ba**			**NBO 64 = LP of Ba**		
	**99.53 %**	**0.02 %**	**0.38 %**	**76.43 %**	**20.19 %**	**3.37 %**

**3-Ba**	LUMO = 176			SOMO = 176		
	-0.441*[176]: LV (1)Ba 1(lv)			-0.701*[59]: LP (1)Ba 1(lp)		
	0.336*[236]: BD*(2) C45- C47*			-0.263*[***]: RY (1) H86(ry)		
	0.312*[204]: BD*(2) C13- C15*			0.260*[923]: RY (1) H54(ry)		
	-0.279*[225]: BD*(2) C40- C41*			0.244*[***]: RY (1) H85(ry)		
	-0.259*[193]: BD*(2) C 8- C 9*			-0.231*[929]: RY (1) H55(ry)		
	**NBO 176 = LV of Ba**			**NBO 59 = LP of Ba**		
	**99.19 %**	**0.04 %**	**0.65 %**	**77.80 %**	**11.30 %**	**10.89 %**

We note that in the absence of THF for **3-Ba**, the contribution from *d* orbitals in the SOMO for the dianion is notably higher than when THF is coordinated by 7.5 %.

4-Sr

	Complex	Monoanion
	$[Sr\{N(SiMe_3)_2\}_2(dioxane)_2]$	$[Sr\{N(SiMe_3)_2\}_2(dioxane)_2]^{-}$

**4-Sr**	LUMO = 143			SOMO = 143		
	-0.746*[143]: LV (1)Sr42(lv)			0.864*[57]: LP (1)Sr42(lp)		
	**NBO 143 = LV of Sr**			**NBO 143 = LP of Sr**		
	**98.72 %**	**0.01 %**	**1.27 %**	**86.54 %**	**5.61 %**	**7.84 %**

Cartesian Coordinates and Computed Energies [in Hartrees] for Calculated Structures

1-Mg

SCF (BP86) Energy = -907.433375903
Enthalpy 0K = -906.765223
Enthalpy 298K = -906.710534
Free Energy 298K = -906.850646
Lowest Frequency = 20.1397 cm^{-1}
Second Frequency = 22.6322 cm^{-1}
SCF (BP86-D3BJ) Energy = -907.646897280
SCF (THF) Energy = -907.478801167
SCF (BS2) Energy = -2820.70000594

```
C   1.70455  1.64262  2.48403
H   0.84278  2.13936  2.00394
H   2.19419  2.36986  3.15659
H   1.32287  0.81356  3.10645
C   3.77597  2.54123  0.39073
H   4.59505  2.25056 -0.28927
H   4.20443  3.19128  1.17603
H   3.05949  3.14183 -0.19427
C   4.32057  0.08058  2.10826
H   3.93671 -0.83170  2.59638
H   4.74872  0.73193  2.89273
H   5.14744 -0.21852  1.44015
C   1.70833 -1.64061 -2.48201
H   0.84897 -2.14064 -2.00106
H   2.20032 -2.36601 -3.15484
H   1.32277 -0.81330 -3.10438
C   4.31700 -0.06645 -2.11100
H   3.92686  0.84276 -2.59994
H   4.74790 -0.71637 -2.89516
H   5.14303  0.23871 -1.44460
C   3.78659 -2.52889 -0.39062
H   4.60590 -2.23376  0.28715
H   4.21603 -3.17822 -1.17596
H   3.07391 -3.13162  0.19685
C   0.56663 -2.27817  2.48517
H   1.42682 -1.78478  1.99870
H   0.95267 -3.06076  3.16302
H   0.04196 -1.52762  3.10317
C   0.30070 -4.54223  0.41614
H  -0.36245 -5.11235 -0.25686
H   0.64738 -5.23023  1.20947
H   1.18007 -4.23191 -0.17270
C  -2.09951 -3.75685  2.12558
H  -2.69453 -2.96112  2.60580
H  -1.75102 -4.44592  2.91724
H  -2.77437 -4.32834  1.46418
C  -2.20451 -3.70605 -2.11919
H  -1.22003 -3.82190 -2.60426
H  -2.97955 -3.75441 -2.90657
H  -2.35535 -4.57443 -1.45390
C  -4.08106 -2.01596 -0.40788
H  -4.24062 -2.87503  0.26612
H  -4.85434 -2.05882 -1.19720
H  -4.24734 -1.09897  0.18183
C  -2.26331 -0.66117 -2.49162
H  -2.27010  0.33405 -2.01285
H  -3.13410 -0.72651 -3.16871
H  -1.35108 -0.74081 -3.10962
C  -2.26538  0.65528  2.49214
H  -2.26898 -0.34002  2.01351
H  -3.13664  0.71814  3.16886
H  -1.35362  0.73781  3.11047
C  -4.08620  2.00421  0.40706
H  -4.24833  2.86348 -0.26608
H  -4.86012  2.04354  1.19593
H  -4.24897  1.08735 -0.18383
C  -2.21626  3.70017  2.11946
H  -1.23261  3.81934  2.60542
H  -2.99212  3.74584  2.90620
H  -2.36950  4.56811  1.45414
C   0.56313  2.27974 -2.48269
H   1.42413  1.78951 -1.99445
H   0.94764  3.06327 -3.16031
H   0.04210  1.52707 -3.10120
C  -2.10955  3.74742 -2.12868
H  -2.70083  2.94852 -2.60825
H  -1.76245  4.43624 -2.92117
H  -2.78759  4.31771 -1.46949
C   0.28468  4.54538 -0.41697
H  -0.38117  5.11256  0.25582
H   0.62764  5.23463 -1.21083
H   1.16584  4.24000  0.17178
Mg  0.00007 -0.00003  0.00037
N   2.06252  0.00240  0.00046
N  -1.02946 -1.78672 -0.00213
N  -1.03374  1.78430  0.00309
Si  2.92280  0.99889  1.15649
Si  2.92543 -0.99102 -1.15625
Si -0.60313 -3.02072  1.16551
Si -2.31491 -2.03895 -1.16462
Si -2.32058  2.03265  1.16487
Si -0.61147  3.01887 -1.16543
```

1-Mg (dianion)

SCF (BP86) Energy = -907.295487600
Enthalpy 0K = -906.631455
Enthalpy 298K = -906.576421
Free Energy 298K = -906.717528
Lowest Frequency = 20.3353 cm^{-1}
Second Frequency = 23.1159 cm^{-1}
SCF (BP86-D3BJ) Energy = -907.504049999
SCF (THF) Energy = -907.466492863
SCF (BS2) Energy = -2820.59771620

```
C   0.30563 -2.23619  2.33765
H   1.14892 -1.76461  1.80227
H   0.68961 -3.06281  2.96461
H  -0.14606 -1.47775  3.00033
C  -0.19277 -4.44985  0.27577
H  -0.90273 -4.94155 -0.41249
H   0.09146 -5.18430  1.05466
H   0.71171 -4.20174 -0.30415
C  -2.43110 -3.56689  2.10839
H  -2.97501 -2.75916  2.62843
H  -2.04419 -4.26519  2.87659
H  -3.16142 -4.12302  1.49324
C  -3.01079 -0.48791 -2.43894
H  -2.86795  0.53853 -2.06072
H  -4.01752 -0.56029 -2.90360
H  -2.23754 -0.64260 -3.21165
C  -3.02515 -3.48748 -1.94371
H  -2.15201 -3.66437 -2.59671
H  -3.93477 -3.50524 -2.57562
H  -3.09348 -4.33229 -1.23428
C  -4.47728 -1.62024 -0.02494
H  -4.60693 -2.44794  0.69360
H  -5.35632 -1.61409 -0.69916
```

```
H  -4.48543 -0.67779  0.54951
C  -2.07750  0.84386  2.34497
H  -2.09010 -0.12020  1.80579
H  -2.98536  0.92160  2.97234
H  -1.19508  0.85350  3.00789
C  -3.75614  2.40559  0.31041
H  -3.82793  3.26840 -0.37510
H  -4.52233  2.53314  1.10008
H  -4.01098  1.50199 -0.26793
C  -1.84326  3.87911  2.12920
H  -0.86391  3.94047  2.63490
H  -2.62976  3.89074  2.90940
H  -1.96664  4.79356  1.52123
C  -1.52384  4.33866 -1.96225
H  -2.11376  3.66215 -2.60596
H  -1.08925  5.13000 -2.60407
H  -2.22061  4.82540 -1.25556
C   0.81830  4.69154 -0.04783
H   0.16428  5.22486  0.66352
H   1.26269  5.44275 -0.73008
H   1.63883  4.23613  0.53322
C   1.07873  2.83703 -2.44292
H   1.89884  2.20872 -2.05584
H   1.51572  3.74420 -2.91279
H   0.56566  2.23530 -3.21338
C   1.75683  1.35833  2.35745
H   0.93205  1.85853  1.81942
H   2.27532  2.09763  2.99652
H   1.31934  0.58186  3.00851
C   3.94955  2.05671  0.33478
H   4.73529  1.69829 -0.35337
H   4.43891  2.65203  1.13042
H   3.29092  2.73094 -0.23767
C   4.27330 -0.35238  2.13610
H   3.83465 -1.23349  2.63603
H   4.67543  0.31841  2.92077
H   5.12773 -0.70040  1.52796
C   1.96394 -2.31883 -2.47840
H   1.00808 -2.73588 -2.11852
H   2.54903 -3.13231 -2.95885
H   1.70291 -1.55808 -3.23470
C   4.55624 -0.83091 -1.92023
H   4.28219  0.02690 -2.55982
H   5.03630 -1.59643 -2.56094
H   5.31128 -0.48178 -1.19246
C   3.64572 -3.05903 -0.05558
H   4.41779 -2.76945  0.67805
H   4.08987 -3.80966 -0.73861
H   2.82786 -3.54985  0.49971
Mg -0.00263 -0.00233 -0.68821
N  -1.38317 -1.61322 -0.09375
N  -0.70926  1.99866 -0.09431
N   2.08703 -0.38418 -0.09578
Si -0.96446 -2.85954  1.05382
Si -2.83901 -1.78486 -1.04293
Si -1.98483  2.26155  1.06754
Si -0.13678  3.34096 -1.05367
Si  2.94447  0.58365  1.07722
Si  2.97784 -1.54114 -1.05405
```

1-Ca

SCF (BP86) Energy = -943.456933537
Enthalpy 0K = -942.792085
Enthalpy 298K = -942.735806
Free Energy 298K = -942.883366
Lowest Frequency = 17.6145 cm^{-1}
Second Frequency = 18.9110 cm^{-1}
SCF (BP86-D3BJ) Energy = -943.664008517
SCF (THF) Energy = -943.502267486
SCF (BS2) Energy = -3298.30081356

```
C  -1.11966 -2.13805 -2.46017
H  -1.82003 -1.44433 -1.95696
H  -1.70664 -2.72872 -3.18663
H  -0.38916 -1.54067 -3.03660
C  -1.57483 -4.44906 -0.47903
H  -1.13508 -5.17823  0.22360
H  -2.08621 -5.01579 -1.27899
H  -2.33979 -3.87775  0.07477
C   0.96169 -4.39047 -2.20038
H   1.76087 -3.79073 -2.66930
H   0.42427 -4.92906 -3.00277
H   1.44627 -5.14799 -1.55887
C   1.95982 -1.40436  2.45950
H   2.27379 -0.46988  1.95632
H   2.74915 -1.66785  3.18663
H   1.03829 -1.19910  3.03514
C   1.11517 -4.35261  2.20054
H   0.13097 -4.17704  2.66829
H   1.83673 -4.59081  3.00386
H   1.02458 -5.24764  1.55951
C   3.40748 -3.26402  0.48003
H   3.34372 -4.11433 -0.22117
H   4.11911 -3.53820  1.28067
H   3.83313 -2.41025 -0.07509
C   2.41221  0.09935 -2.46041
H   2.15910 -0.85391 -1.95813
H   3.21810 -0.11482 -3.18557
H   1.53120  0.43519 -3.03812
C   4.63937  0.85437 -0.47543
H   5.05240  1.59875  0.22745
H   5.38634  0.69235 -1.27444
H   4.52347 -0.09324  0.07852
C   3.32834  3.02626 -2.19717
H   2.41115  3.42097 -2.66753
H   4.06477  2.82952 -2.99820
H   3.74234  3.82311 -1.55386
C   3.21244  3.14532  2.19782
H   3.55190  2.20600  2.66760
H   3.05882  3.89127  2.99950
H   4.03292  3.51225  1.55567
C   1.12317  4.58408  0.47591
H   1.89146  4.95323 -0.22570
H   1.00460  5.33838  1.27565
H   0.17099  4.52496 -0.07919
C   0.23655  2.40316  2.45818
H  -0.72949  2.20651  1.95506
H   0.06941  3.21964  3.18386
H   0.51984  1.50354  3.03538
C  -1.29464  2.03946 -2.46029
H  -0.34300  2.29932 -1.95845
H  -1.51383  2.84307 -3.18648
H  -1.14326  1.10811 -3.03684
C  -3.06423  3.58925 -0.47582
H  -3.91530  3.57360  0.22717
H  -3.29867  4.31639 -1.27514
H  -2.18616  3.96430  0.07787
C  -4.28627  1.36493 -2.19626
H  -4.16795  0.37306 -2.66576
H  -4.48499  2.10007 -2.99800
H  -5.18348  1.32431 -1.55317
C  -2.19699 -0.99530  2.46025
H  -1.54317 -1.73329  1.95732
H  -2.81989 -1.54851  3.18628
H  -1.56008 -0.29934  3.03704
C  -4.33057  1.20806  2.19893
```

```
H  -3.68800  1.97319  2.66791
H  -4.89869  0.70169  3.00113
H  -5.05964  1.73342  1.55655
C  -4.52845 -1.32191  0.47827
H  -5.23214 -0.84265 -0.22450
H  -5.12261 -1.80151  1.27800
H  -3.99974 -2.11695 -0.07548
Ca -0.00012  0.00046 -0.00175
N   0.53777 -2.26415 -0.00063
N   1.69211  1.59855 -0.00114
N  -2.23029  0.66646  0.00009
Si -0.23292 -3.27167 -1.18938
Si  1.67883 -2.81622  1.18912
Si  2.95131  1.43234 -1.18809
Si  1.59978  2.86407  1.18722
Si -2.71806  1.83854 -1.18756
Si -3.27901 -0.04702  1.18897
```

1-Ca (dianion)

SCF (BP86) Energy = -943.337078886
Enthalpy 0K = -942.674665
Enthalpy 298K = -942.618643
Free Energy 298K = -942.765012
Lowest Frequency = 14.7374 cm^{-1}
Second Frequency = 18.0468 cm^{-1}
SCF (BP86-D3BJ) Energy = -943.542755968
SCF (THF) Energy = -943.507067508
SCF (BS2) Energy = -3298.20867055

```
C  -2.85992  1.31519 -2.38059
H  -2.18393  2.08922 -1.97295
H  -3.68631  1.83244 -2.90959
H  -2.26917  0.74553 -3.12310
C  -4.72320  1.28960  0.03471
H  -5.28974  0.71313  0.78688
H  -5.45230  1.81105 -0.61546
H  -4.14120  2.05817  0.57270
C  -4.72727 -1.06647 -1.91431
H  -4.14159 -1.72517 -2.57989
H  -5.47559 -0.53241 -2.53173
H  -5.27631 -1.70839 -1.20061
C  -1.15206 -2.02899  2.29612
H  -0.20324 -2.16447  1.74508
H  -1.30208 -2.90199  2.95963
H  -1.05055 -1.12925  2.92848
C  -4.16357 -1.53134  2.17763
H  -4.10553 -0.55693  2.69341
H  -4.24162 -2.32111  2.95001
H  -5.10046 -1.54532  1.59175
C  -2.90669 -3.57346  0.30892
H  -3.77009 -3.56579 -0.37978
H  -3.10095 -4.33046  1.09366
H  -2.02656 -3.90242 -0.26988
C   0.29565 -3.11332 -2.39129
H  -0.71338 -2.92432 -1.98094
H   0.26548 -4.08244 -2.92995
H   0.48895 -2.30880 -3.12646
C   1.25604 -4.73164  0.00993
H   2.04248 -4.94055  0.75618
H   1.16682 -5.61736 -0.64859
H   0.30205 -4.61830  0.55390
C   3.29315 -3.53483 -1.93040
H   3.56869 -2.69115 -2.58778
H   3.20594 -4.44384 -2.55689
H   4.12447 -3.69507 -1.21905
C   3.36779 -2.84363  2.20317
H   2.48263 -3.27739  2.70030
H   4.07559 -2.51891  2.99055
H   3.85766 -3.64960  1.62736
C   4.55196 -0.74678  0.34778
H   4.97738 -1.49917 -0.33975
H   5.29678 -0.54968  1.14338
H   4.41503  0.18508 -0.22733
C   2.31605  0.02186  2.30224
H   1.96628  0.91117  1.74621
H   3.14331  0.32713  2.97112
H   1.48014 -0.33482  2.92971
C   2.58323  1.77544 -2.35682
H   2.90360  0.80857 -1.92694
H   3.45400  2.21681 -2.88317
H   1.80230  1.54127 -3.10556
C   3.47842  3.44541  0.03391
H   3.25821  4.23664  0.77184
H   4.29881  3.80501 -0.61710
H   3.84929  2.56597  0.58885
C   1.45513  4.59493 -1.95010
H   0.59786  4.40457 -2.62000
H   2.29701  4.96595 -2.56656
H   1.16680  5.40321 -1.25264
C  -1.19230  2.02395  2.28698
H  -1.78366  1.27049  1.73479
H  -1.87506  2.59428  2.94540
H  -0.46969  1.48494  2.92507
C   0.75972  4.36951  2.17828
H   1.57082  3.82581  2.69347
H   0.11518  4.83150  2.95128
H   1.22129  5.18837  1.59717
C  -1.62940  4.32012  0.29861
H  -1.18347  5.05884 -0.39080
H  -2.18419  4.87315  1.08152
H  -2.35862  3.72762 -0.28020
Ca -0.00217  0.00323 -0.72901
N  -2.26478 -0.58142 -0.10117
N   1.63676 -1.66388 -0.10159
N   0.62829  2.25237 -0.10249
Si -3.54643  0.16854 -1.01235
Si -2.60427 -1.82486  1.06652
Si  1.63277 -3.14199 -1.02384
Si  2.87043 -1.34436  1.08211
Si  1.92585  2.97076 -1.01607
Si -0.27725  3.17417  1.06086
```

1-Sr

SCF (BP86) Energy = -937.354628064
Enthalpy 0K = -936.690977
Enthalpy 298K = -936.634087
Free Energy 298K = -936.785624
Lowest Frequency = 16.4110 cm^{-1}
Second Frequency = 16.8834 cm^{-1}
SCF (BP86-D3BJ) Energy = -937.556805576
SCF (THF) Energy = -937.400821775
SCF (BS2) Energy = -2651.38062510

```
C  -2.44885 -0.35317  2.44449
H  -3.16289 -0.71750  3.20515
H  -1.62610  0.14844  2.98846
H  -2.04109 -1.24736  1.93378
C  -4.83559 -0.07663  0.52528
H  -5.40630  0.55883 -0.17443
H  -5.52033 -0.38828  1.33560
H  -4.52887 -0.98194 -0.02721
C  -3.97793  2.31203  2.25432
H  -3.15313  2.87945  2.71928
H  -4.64538  1.95680  3.06115
H  -4.55577  3.01726  1.63055
C  -3.89416  2.44562 -2.25340
```

```
H  -4.01697  1.44897 -2.71171
H  -3.88708  3.19625 -3.06528
H  -4.78643  2.63704 -1.63086
C  -2.17434  4.31676 -0.52853
H  -3.00209  4.52953  0.17048
H  -2.21483  5.06649 -1.34022
H  -1.23008  4.46491  0.02402
C  -0.82427  2.32591 -2.44415
H  -0.83319  3.12575 -3.20664
H   0.15761  2.38043 -1.93460
H  -0.88789  1.36317 -2.98589
C   0.92132  2.28994  2.44846
H  -0.05782  2.38552  1.93972
H   0.96391  3.08796  3.21171
H   0.94540  1.32484  2.98924
C   2.34970  4.22454  0.53228
H   3.18536  4.40429 -0.16658
H   2.41988  4.97190  1.34413
H   1.41217  4.41025 -0.02043
C   3.99358  2.28376  2.25482
H   4.07593  1.28248  2.71206
H   4.01789  3.03334  3.06733
H   4.89240  2.43904  1.63159
C   2.42675 -0.44809 -2.44528
H   3.12379 -0.84046 -3.20776
H   1.98338 -1.32545 -1.93505
H   1.62446  0.08782 -2.98706
C   4.06304  2.15225 -2.25543
H   3.26063  2.75301 -2.71769
H   4.71371  1.77166 -3.06442
H   4.67037  2.83285 -1.63229
C   4.82763 -0.27248 -0.53244
H   5.42701  0.33840  0.16514
H   5.49567 -0.61222 -1.34539
H   4.48530 -1.16432  0.02103
C   2.48223 -4.15220  0.53217
H   2.21898 -4.96400 -0.16838
H   3.09156 -4.58973  1.34451
H   3.11507 -3.43416 -0.01816
C  -0.02231 -4.60159  2.25233
H  -0.93021 -4.17102  2.70925
H   0.61336 -4.99930  3.06497
H  -0.33831 -5.45646  1.62806
C   1.52456 -1.94644  2.44830
H   2.09870 -1.14767  1.93940
H   2.19333 -2.38372  3.21168
H   0.67775 -1.48275  2.98896
C  -0.16806 -4.59059 -2.25915
H   0.75739 -4.19827 -2.71523
H  -0.82095 -4.95730 -3.07274
H   0.11073 -5.46070 -1.63821
C  -2.65058 -4.03854 -0.53690
H  -2.42364 -4.86439  0.15990
H  -3.27886 -4.44493 -1.35098
H  -3.25083 -3.29560  0.01686
C  -1.59725 -1.87063 -2.44568
H  -2.28657 -2.27589 -3.20843
H  -2.13402 -1.04749 -1.93470
H  -0.73129 -1.44498 -2.98732
N  -2.13044  1.28833  0.00164
N   2.18174  1.20087  0.00113
N  -0.04900 -2.48954  0.00043
Si -3.29602  0.85075  1.20529
Si -2.28316  2.52138 -1.20471
Si  2.38603  2.42601  1.20783
Si  3.32542  0.71826 -1.20639
Si  0.90783 -3.28139  1.20707
Si -1.03942 -3.23507 -1.20858
Sr  0.00054 -0.00001  0.00146
```

1-Sr (dianion)

SCF (BP86) Energy = -937.244838234
Enthalpy 0K = -936.582668
Enthalpy 298K = -936.526281
Free Energy 298K = -936.674671
Lowest Frequency = 15.9666 cm^{-1}
Second Frequency = 21.2378 cm^{-1}
SCF (BP86-D3BJ) Energy = -937.448017734
SCF (THF) Energy = -937.411335013
SCF (BS2) Energy = -2651.29264063

```
C   1.59722 -2.83067 -2.32582
H   1.95286 -3.72346 -2.87729
H   1.45291 -2.01521 -3.06161
H   0.59808 -3.07414 -1.91775
C   3.06815 -3.94544  0.10275
H   3.85365 -3.82926  0.86978
H   3.34140 -4.80667 -0.53707
H   2.12815 -4.19720  0.62434
C   4.53377 -2.09268 -1.84519
H   4.47482 -1.22254 -2.52275
H   4.81287 -2.97845 -2.44801
H   5.35156 -1.90428 -1.12518
C   4.24377 -1.33964  2.31009
H   3.57952 -2.07446  2.79762
H   4.74805 -0.75672  3.10493
H   5.02322 -1.90269  1.76538
C   4.57359  1.02832  0.42454
H   5.27843  0.49296 -0.23616
H   5.15966  1.51502  1.22816
H   4.09799  1.82302 -0.17616
C   2.15093  0.88914  2.30264
H   2.76784  1.49287  2.99508
H   1.50763  1.57667  1.72198
H   1.49116  0.24204  2.90761
C   1.73427  2.76061 -2.25968
H   2.42277  2.02175 -1.80839
H   2.35526  3.50266 -2.79921
H   1.12809  2.21931 -3.01300
C   1.86762  4.65236  0.13032
H   1.34440  5.29314  0.86152
H   2.49839  5.30450 -0.50416
H   2.53765  3.98094  0.69550
C  -0.40127  4.93163 -1.90919
H  -1.09344  4.42082 -2.60188
H   0.24228  5.60832 -2.50409
H  -1.00681  5.55624 -1.22669
C  -1.87000  1.43868  2.28886
H  -2.71024  1.67416  2.96940
H  -2.13388  0.53158  1.71329
H  -0.98593  1.19934  2.90638
C  -0.99766  4.36838  2.28376
H  -0.03386  4.17028  2.78447
H  -1.76507  4.51510  3.06824
H  -0.89896  5.32119  1.73269
C  -3.18084  3.44865  0.37235
H  -3.06000  4.32185 -0.29328
H  -3.90910  3.71760  1.16193
H  -3.61954  2.63419 -0.22985
C  -4.95596 -0.68210  0.15705
H  -5.23720 -1.43669  0.91234
H  -5.84709 -0.47566 -0.46666
H  -4.69743  0.24693  0.69469
C  -4.11120 -2.83295 -1.85204
H  -3.33772 -3.19981 -2.54985
H  -5.02590 -2.61668 -2.43732
```

```
H  -4.34776 -3.65321 -1.14925
C  -3.28267  0.08606 -2.27701
H  -2.97833  1.06134 -1.85290
H  -4.24159  0.24362 -2.80922
H  -2.51920 -0.18847 -3.03120
C  -3.27356 -3.07842  2.27063
H  -3.58047 -2.15558  2.79331
H  -3.01589 -3.83408  3.03772
H  -4.15055 -3.45522  1.71373
C  -1.37292 -4.47332  0.34189
H  -2.18713 -4.80552 -0.32652
H  -1.23181 -5.24686  1.12155
H  -0.44967 -4.42826 -0.26164
C  -0.30588 -2.34457  2.28069
H  -0.08350 -3.19267  2.95580
H   0.60926 -2.11095  1.70482
H  -0.54530 -1.46435  2.90365
N   2.26920 -0.96120 -0.06542
N  -0.29744  2.45001 -0.06503
N  -1.97616 -1.48035 -0.06463
Si  2.83886 -2.34192 -0.95177
Si  3.23928 -0.17059  1.13709
Si  0.63728  3.61927 -0.94510
Si -1.48569  2.90248  1.11560
Si -3.46634 -1.26238 -0.92962
Si -1.76316 -2.74440  1.10497
Sr -0.00372  0.00168 -0.83762
```

1-Ba

SCF (BP86) Energy = -932.133453327
Enthalpy 0K = -931.470585
Enthalpy 298K = -931.413311
Free Energy 298K = -931.568561
Lowest Frequency = 10.6702 cm^{-1}
Second Frequency = 12.7827 cm^{-1}
SCF (BP86-D3BJ) Energy = -932.331860090
SCF (THF) Energy = -932.181805228
SCF (BS2) Energy = -2646.17959826

```
C  -0.85200  2.41761  2.44589
H  -1.32522  3.02488  3.23880
H  -0.19293  1.68870  2.95561
H  -1.66939  1.86554  1.94254
C  -1.10877  4.84264  0.58104
H  -0.61850  5.55566 -0.10479
H  -1.56048  5.42245  1.40709
H  -1.92604  4.35524  0.02097
C   1.41799  4.48537  2.29589
H   2.14953  3.79004  2.74306
H   0.92917  5.04221  3.11663
H   1.98277  5.21433  1.68774
C   1.49862  4.45960 -2.30315
H   0.49549  4.33690 -2.74731
H   2.22047  4.61328 -3.12658
H   1.48206  5.38332 -1.69754
C   3.74455  3.25020 -0.58908
H   3.77253  4.11438  0.09764
H   4.45022  3.45150 -1.41620
H   4.11653  2.37320 -0.03080
C   2.09746  1.44861 -2.45240
H   2.83579  1.65773 -3.24770
H   2.43054  0.51934 -1.95058
H   1.13268  1.25243 -2.95853
C   2.50174 -0.46834  2.45402
H   2.42799  0.51528  1.95054
H   3.25826 -0.35649  3.25185
H   1.53930 -0.68249  2.95745
C   4.74718 -1.44239  0.59976
H   5.12870 -2.22112 -0.08375
H   5.47062 -1.33502  1.42904
H   4.72915 -0.49141  0.03895
C   3.18320 -3.46232  2.30989
H   2.21547 -3.75723  2.75166
H   3.90324 -3.30904  3.13496
H   3.54462 -4.31337  1.70544
C   0.22068 -2.54945 -2.45037
H   0.03837 -3.29308 -3.24732
H  -0.75249 -2.37992 -1.94990
H   0.52858 -1.61320 -2.95440
C   3.13421 -3.51664 -2.29591
H   3.52253 -2.58271 -2.73821
H   2.91473 -4.21997 -3.12045
H   3.94462 -3.95739 -1.68824
C   0.96725 -4.87547 -0.59080
H   1.70312 -5.32974  0.09564
H   0.79347 -5.58603 -1.41997
H   0.02048 -4.76527 -0.03345
C  -3.66751 -3.36830  0.63689
H  -4.53119 -3.30646 -0.04814
H  -3.94783 -4.03019  1.47708
H  -2.84225 -3.85089  0.08448
C  -4.59512 -0.96294  2.31239
H  -4.35113  0.02752  2.73438
H  -4.82885 -1.64420  3.15140
H  -5.51351 -0.84886  1.70928
C  -1.67594 -1.91249  2.46466
H  -0.79403 -2.35873  1.96531
H  -1.96534 -2.60922  3.27222
H  -1.36668 -0.96968  2.95565
C  -4.59750 -0.97282 -2.31791
H  -3.98189 -1.78402 -2.74407
H  -5.08322 -0.43690 -3.15434
H  -5.39502 -1.44349 -1.71568
C  -4.69929  1.59839 -0.63630
H  -5.46984  1.19685  0.04501
H  -5.21675  2.09912 -1.47541
H  -4.13389  2.36578 -0.07898
C  -2.29594  1.05713 -2.46963
H  -2.83979  1.57962 -3.27744
H  -1.66516  1.81898 -1.97160
H  -1.63657  0.31511 -2.95977
N   0.81534  2.51564 -0.00293
N   1.77597 -1.96784  0.00369
N  -2.59589 -0.55641 -0.00355
Si  0.13985  3.52978  1.22214
Si  1.96016  2.93208 -1.22845
Si  2.98634 -1.88026  1.23340
Si  1.57530 -3.16600 -1.22466
Si -3.13907 -1.62260  1.24242
Si -3.52101  0.20791 -1.24640
Ba -0.00336 -0.00404  0.00166
```

1-Ba (dianion)

SCF (BP86) Energy = -932.034827974
Enthalpy 0K = -931.372842
Enthalpy 298K = -931.316192
Free Energy 298K = -931.466435
Lowest Frequency = 16.3943 cm^{-1}
Second Frequency = 19.8745 cm^{-1}
SCF (BP86-D3BJ) Energy = -932.237351158
SCF (THF) Energy = -932.197878596
SCF (BS2) Energy = -2646.09948586

```
C   2.96497  1.50877 -2.20325
H   3.88284  1.80166 -2.74828
H   2.16814  1.39631 -2.96561
```

H 3.15756 0.50832 -1.77053
C 4.11273 2.97402 0.22028
H 4.01500 3.77596 0.97288
H 5.00465 3.19327 -0.39742
H 4.29929 2.03012 0.76182
C 2.39251 4.49564 -1.81142
H 1.54032 4.46333 -2.51309
H 3.30787 4.71331 -2.39463
H 2.22612 5.34022 -1.11736
C 1.48354 4.29280 2.37777
H 2.17882 3.60199 2.88607
H 0.90138 4.82331 3.15559
H 2.09114 5.04655 1.84482
C -0.82762 4.69646 0.42834
H -0.25042 5.38903 -0.20986
H -1.32999 5.29223 1.21467
H -1.61001 4.24179 -0.20438
C -0.80588 2.26420 2.30691
H -1.39090 2.89297 3.00456
H -1.51823 1.65654 1.71745
H -0.19098 1.57025 2.90783
C -2.82258 1.78444 -2.22717
H -2.05204 2.46143 -1.81092
H -3.54497 2.42211 -2.77214
H -2.32994 1.14461 -2.98614
C -4.64257 2.06561 0.20966
H -5.27630 1.58722 0.97684
H -5.28970 2.71486 -0.41073
H -3.91672 2.71201 0.73344
C -5.10399 -0.21716 -1.78617
H -4.65097 -0.94962 -2.47751
H -5.75847 0.45210 -2.37698
H -5.74359 -0.77419 -1.07678
C -1.53936 -1.81352 2.31301
H -1.78673 -2.63021 3.01739
H -0.66042 -2.13001 1.72020
H -1.24292 -0.93003 2.90650
C -4.44241 -0.85151 2.39440
H -4.19159 0.10063 2.89391
H -4.60286 -1.61566 3.17907
H -5.40308 -0.70934 1.86695
C -3.64030 -3.06592 0.45472
H -4.53049 -2.92014 -0.18281
H -3.90140 -3.79291 1.24780
H -2.85545 -3.51967 -0.17571
C 0.51427 -5.04141 0.19204
H 1.24229 -5.36030 0.95849
H 0.27025 -5.92138 -0.43349
H -0.40708 -4.73246 0.71622
C 2.72873 -4.30635 -1.79781
H 3.14285 -3.54834 -2.48593
H 2.46972 -5.20345 -2.39231
H 3.52826 -4.59088 -1.08896
C -0.13816 -3.30465 -2.23496
H -1.10925 -2.97846 -1.81530
H -0.33169 -4.24149 -2.79191
H 0.17252 -2.55021 -2.98531
C 2.93783 -3.43533 2.39265
H 1.98309 -3.68847 2.88596
H 3.67806 -3.20320 3.18229
H 3.28958 -4.33844 1.86169
C 4.48003 -1.64040 0.46803
H 4.79324 -2.48571 -0.17030
H 5.23784 -1.51312 1.26501
H 4.49277 -0.73215 -0.15974
C 2.34671 -0.43409 2.32163
H 3.18170 -0.24365 3.02226
H 2.18008 0.48733 1.73232
H 1.43629 -0.61877 2.91970
N 1.09770 2.33960 -0.01050
N -2.57386 -0.21848 -0.00887
N 1.47901 -2.11256 -0.01180
Si 2.52580 2.81635 -0.86879
Si 0.31246 3.33067 1.17365
Si -3.71443 0.76132 -0.87074
Si -3.02924 -1.38836 1.18542
Si 1.19151 -3.58477 -0.87993
Si 2.71538 -1.93573 1.18943
Ba -0.00048 0.00394 -0.88461

3-Mg

SCF (BP86) Energy = -1613.39577174
Enthalpy 0K = -1612.545758
Enthalpy 298K = -1612.483765
Free Energy 298K = -1612.639682
Lowest Frequency = 13.5219 cm^{-1}
Second Frequency = 27.9894 cm^{-1}
SCF (BP86-D3BJ) Energy = -1613.68035107
SCF (THF) Energy = -1613.41675203
SCF (BS2) Energy = -3241.56044455

K -3.76371 1.46033 0.50692
Si 0.70834 1.74967 -2.28294
Si -0.80355 -3.54738 -1.09184
Mg 0.47195 -0.50944 -0.18959
N -0.18985 1.45211 -0.76243
N -1.07752 -1.89226 -0.49079
N 2.29557 -0.80335 0.77195
C -0.88472 2.33247 0.05210
C -1.05277 2.02595 1.46500
C -1.75441 2.88234 2.33514
H -1.80962 2.60205 3.39629
C -2.37404 4.07234 1.90692
C -2.27248 4.35058 0.53014
H -2.75325 5.25801 0.13855
C -1.56911 3.53902 -0.38348
C -0.54575 0.72542 2.03241
H -0.70673 0.67377 3.12102
H 0.53990 0.57057 1.87696
H -1.08610 -0.14945 1.61044
C -3.05429 5.02034 2.87145
H -3.47980 4.48801 3.74011
H -3.86938 5.58485 2.38570
H -2.34385 5.76756 3.27280
C -1.60673 3.95776 -1.83436
H -1.97099 3.14960 -2.49122
H -0.61318 4.24896 -2.21563
H -2.26869 4.82988 -1.96782
C -0.18296 2.05582 -3.96619
H 0.49689 1.69095 -4.75737
H -0.38696 3.11654 -4.17713
H -1.12676 1.49567 -4.06979
C 2.04174 3.09431 -2.09213
H 2.79382 2.78676 -1.34668
H 1.61449 4.05790 -1.76587
H 2.55916 3.26356 -3.05293
C 1.55602 0.06224 -2.60763
H 2.31717 -0.22021 -1.85764
H 2.10922 0.13842 -3.55980
H 0.82552 -0.75797 -2.75031
C -2.44354 -1.59394 -0.29453
C -3.12446 -1.84907 0.95071
C -4.51387 -1.61519 1.07278
H -4.99939 -1.85828 2.02866
C -5.30673 -1.15624 0.00318
C -4.63384 -0.85090 -1.20110

```
H  -5.21354 -0.48274 -2.05990
C  -3.24338 -1.03024 -1.35744
C  -2.38874 -2.42296  2.14389
H  -2.67408 -3.47555  2.31746
H  -2.62194 -1.86962  3.07105
H  -1.30245 -2.40700  1.97297
C  -6.81459 -1.04697  0.12022
H  -7.30603 -1.98971 -0.18403
H  -7.22494 -0.25370 -0.52932
H  -7.13388 -0.84249  1.15692
C  -2.58571 -0.61480 -2.65046
H  -3.31523 -0.15432 -3.33899
H  -2.11625 -1.46698 -3.16496
H  -1.77923  0.10971 -2.44194
C   1.02092 -3.69993 -1.59388
H   1.21630 -3.18875 -2.55243
H   1.29595 -4.76145 -1.72007
H   1.68795 -3.27032 -0.82986
C  -1.86241 -4.01474 -2.62181
H  -2.93142 -3.77914 -2.48306
H  -1.78573 -5.10372 -2.79220
H  -1.51590 -3.51482 -3.54241
C  -1.22354 -4.91968  0.17822
H  -0.67152 -4.81638  1.12638
H  -0.97140 -5.90591 -0.25159
H  -2.30273 -4.92987  0.40996
C   3.45392 -0.07133  0.34537
C   4.44076 -0.65595 -0.51534
C   5.56172  0.08924 -0.93104
H   6.29435 -0.39533 -1.59056
C   5.78231  1.41349 -0.52270
C   4.83162  1.97687  0.34477
H   4.98395  3.00128  0.71110
C   3.69179  1.27370  0.78051
C   4.32134 -2.08798 -0.98689
H   3.40684 -2.25598 -1.58009
H   4.27449 -2.79825 -0.14349
H   5.18611 -2.36500 -1.61284
C   6.98123  2.20352 -1.00382
H   7.32954  2.91812 -0.23816
H   6.74700  2.79249 -1.91113
H   7.82641  1.54126 -1.25779
C   2.74291  1.97159  1.72880
H   1.78429  2.21866  1.23793
H   3.18006  2.91651  2.09323
H   2.50597  1.34325  2.60454
Si  2.58787 -1.84093  2.18684
C   1.43329 -1.45314  3.67402
H   1.55306 -0.41826  4.03733
H   1.68507 -2.13160  4.50942
H   0.36657 -1.61244  3.44006
C   2.29734 -3.71101  1.88388
H   1.26713 -3.90879  1.54346
H   2.44574 -4.26783  2.82706
H   2.98302 -4.13572  1.13211
C   4.37079 -1.63372  2.83900
H   5.12468 -1.91646  2.08625
H   4.51840 -2.27276  3.72758
H   4.57641 -0.59029  3.13085
```

3-Mg (anion)

SCF (BP86) Energy = -1585.08346892
Enthalpy 0K = -1584.236094
Enthalpy 298K = -1584.175476
Free Energy 298K = -1584.330088
Lowest Frequency = 18.6647 cm^{-1}
Second Frequency = 19.6084 cm^{-1}
SCF (BP86-D3BJ) Energy = -1585.35768210
SCF (THF) Energy = -1585.13115874
SCF (BS2) Energy = -2641.60825223

```
Si -0.90712  1.61838  2.33263
Si  1.70028 -3.11195  1.47593
Mg -0.05842 -0.40776  0.17254
N   0.00194  1.62672  0.81324
N   1.60688 -1.60197  0.56981
N  -1.76276 -1.11366 -0.84347
C   0.45495  2.69633  0.03078
C   0.86241  2.44621 -1.33302
C   1.24964  3.48599 -2.19240
H   1.53308  3.22478 -3.22163
C   1.29698  4.82892 -1.78728
C   0.98706  5.07261 -0.44108
H   1.06624  6.10021 -0.05738
C   0.60027  4.06673  0.46330
C   0.91700  1.03919 -1.87342
H   1.26188  1.03100 -2.92047
H  -0.07618  0.54328 -1.88947
H   1.65186  0.41521 -1.32149
C   1.66124  5.94447 -2.74282
H   2.39432  5.60942 -3.49876
H   2.10199  6.80593 -2.20957
H   0.78235  6.32781 -3.29931
C   0.43743  4.48756  1.90595
H   1.13274  3.94004  2.56574
H  -0.57854  4.32958  2.30634
H   0.65883  5.56353  2.01382
C  -0.04755  2.08135  3.99952
H  -0.52492  1.49472  4.80594
H  -0.13238  3.14545  4.26840
H   1.02250  1.81454  3.98749
C  -2.60436  2.50040  2.27567
H  -3.24476  2.02463  1.51390
H  -2.52507  3.57108  2.02382
H  -3.11291  2.41929  3.25381
C  -1.27983 -0.24189  2.62308
H  -1.92655 -0.70368  1.85515
H  -1.83371 -0.33898  3.57339
H  -0.35085 -0.83041  2.74280
C   2.89474 -1.11681  0.17433
C   3.51735 -1.52829 -1.05035
C   4.79533 -1.04624 -1.40025
H   5.23838 -1.38147 -2.34915
C   5.51151 -0.15412 -0.59085
C   4.88527  0.28049  0.59191
H   5.40223  1.00541  1.23605
C   3.61033 -0.16501  0.97985
C   2.81200 -2.44967 -2.02230
H   3.25455 -3.46265 -2.03374
H   2.87610 -2.05911 -3.05418
H   1.75372 -2.55920 -1.74400
C   6.89911  0.32299 -0.96776
H   7.68666 -0.18602 -0.37826
H   7.01958  1.40702 -0.79140
H   7.11460  0.13037 -2.03301
C   2.98257  0.41194  2.22669
H   3.68606  1.09057  2.73994
H   2.67070 -0.37071  2.93636
H   2.07302  0.98325  1.96261
C  -0.03521 -3.74784  1.94730
H  -0.56105 -3.07304  2.64261
H   0.06427 -4.72862  2.44674
H  -0.66738 -3.88674  1.05648
C   2.69398 -2.99176  3.11930
H   3.69648 -2.56165  2.95292
H   2.82880 -3.99840  3.55654
```

```
H   2.17713 -2.36782  3.86906
C   2.55871 -4.55731  0.54297
H   1.99851 -4.85921 -0.35765
H   2.63215 -5.43791  1.20754
H   3.58211 -4.28498  0.23366
C  -3.05896 -0.63648 -0.49153
C  -3.95572 -1.41451  0.31388
C  -5.22719 -0.91644  0.65924
H  -5.88269 -1.54168  1.28208
C  -5.68610  0.33825  0.22946
C  -4.81788  1.09201 -0.57998
H  -5.15031  2.07056 -0.95402
C  -3.53476  0.64036 -0.94124
C  -3.56527 -2.79530  0.79077
H  -2.55910 -2.80391  1.23864
H  -3.53362 -3.52434 -0.04014
H  -4.28699 -3.17296  1.53575
C  -7.04486  0.86913  0.63804
H  -7.50689  1.46711 -0.16781
H  -6.98226  1.52580  1.52757
H  -7.74023  0.04926  0.89001
C  -2.67746  1.51571 -1.82465
H  -1.80636  1.91468 -1.27587
H  -3.25525  2.37533 -2.20573
H  -2.27911  0.95417 -2.68742
Si -1.73288 -2.21740 -2.22326
C  -0.64258 -1.59279 -3.67629
H  -1.00699 -0.62705 -4.06701
H  -0.67067 -2.32516 -4.50451
H   0.41333 -1.46408 -3.38750
C  -1.04358 -3.96175 -1.81818
H  -0.05433 -3.88832 -1.33577
H  -0.92475 -4.56180 -2.73910
H  -1.70786 -4.51715 -1.13429
C  -3.47998 -2.44852 -2.97634
H  -4.19811 -2.87296 -2.25565
H  -3.42648 -3.12604 -3.84766
H  -3.89272 -1.48439 -3.31880
```

3-Mg (dianion)

SCF (BP86) Energy = -1584.95862023
Enthalpy 0K = -1584.121598
Enthalpy 298K = -1584.060196
Free Energy 298K = -1584.216250
Lowest Frequency = 11.0185 cm^{-1}
Second Frequency = 21.3322 cm^{-1}
SCF (BP86-D3BJ) Energy = -1585.23172566
SCF (THF) Energy = -1585.12299944
SCF (BS2) Energy = -2641.53413960

```
Si -0.93307  1.65471  2.27560
Si  1.64047 -3.07992  1.55010
Mg -0.06702 -0.39074  0.13626
N   0.01739  1.64029  0.78919
N   1.56924 -1.60666  0.60509
N  -1.76370 -1.08604 -0.89424
C   0.50640  2.70232  0.01240
C   0.91303  2.44849 -1.34686
C   1.35009  3.48206 -2.20234
H   1.63235  3.21796 -3.23111
C   1.43600  4.82587 -1.77713
C   1.11526  5.07205 -0.43859
H   1.21701  6.09816 -0.05056
C   0.68923  4.06953  0.46400
C   0.92400  1.04386 -1.89825
H   1.25397  1.03805 -2.95095
H  -0.08221  0.57366 -1.90885
H   1.65346  0.39005 -1.36910
C   1.87040  5.93083 -2.71379
H   2.84250  5.71021 -3.20114
H   1.98299  6.88980 -2.17502
H   1.14587  6.10336 -3.53784
C   0.54161  4.48422  1.90845
H   1.20042  3.89754  2.57457
H  -0.48319  4.38592  2.31507
H   0.82502  5.54689  2.02336
C  -0.09151  2.08300  3.97185
H  -0.61957  1.52527  4.76830
H  -0.12305  3.15213  4.23583
H   0.96228  1.75566  3.98164
C  -2.58357  2.62486  2.19484
H  -3.24770  2.16839  1.43854
H  -2.43474  3.68208  1.91678
H  -3.10224  2.59974  3.17173
C  -1.38865 -0.18703  2.57090
H  -2.07560 -0.61487  1.81510
H  -1.92394 -0.25714  3.53508
H  -0.48496 -0.81805  2.67401
C   2.88127 -1.16406  0.19932
C   3.47255 -1.62358 -1.03361
C   4.78564 -1.19688 -1.38821
H   5.22099 -1.56674 -2.32944
C   5.52868 -0.32115 -0.59262
C   4.93344  0.16214  0.59463
H   5.48168  0.87306  1.22830
C   3.62519 -0.23694  0.98929
C   2.74274 -2.54708 -1.97788
H   3.21971 -3.54844 -2.06102
H   2.70928 -2.13766 -3.00886
H   1.70901 -2.70320 -1.63340
C   6.92116  0.11866 -0.99673
H   7.67726 -0.12910 -0.22335
H   6.98144  1.21527 -1.14834
H   7.23879 -0.36434 -1.93852
C   3.01925  0.39400  2.22387
H   3.73003  1.10780  2.67952
H   2.73967 -0.34557  2.99304
H   2.09466  0.94491  1.96440
C  -0.09766 -3.78977  1.91155
H  -0.74756 -3.07531  2.44290
H   0.00167 -4.68940  2.54759
H  -0.61142 -4.08675  0.98355
C   2.48358 -2.92620  3.28205
H   3.50381 -2.51567  3.19130
H   2.55846 -3.92091  3.76241
H   1.91104 -2.26707  3.95818
C   2.63129 -4.50785  0.71813
H   2.12242 -4.87835 -0.18806
H   2.75265 -5.35799  1.41587
H   3.63469 -4.15883  0.42054
C  -3.06380 -0.60355 -0.51181
C  -3.95271 -1.40456  0.29499
C  -5.23183 -0.88762  0.66448
H  -5.88940 -1.51197  1.28958
C  -5.66544  0.38156  0.27644
C  -4.79737  1.16691 -0.52009
H  -5.12368  2.15601 -0.87050
C  -3.51338  0.68465 -0.92141
C  -3.59482 -2.80730  0.71787
H  -2.50298 -2.94167  0.74372
H  -3.99198 -3.59276  0.03138
H  -4.00443 -3.04490  1.72046
C  -7.02041  0.91174  0.69797
H  -7.64282  1.20204 -0.17397
H  -6.93455  1.81946  1.32996
H  -7.58701  0.15810  1.27470
```

```
C  -2.65556  1.56804 -1.79730
H  -1.80288  2.01495 -1.24952
H  -3.25266  2.40195 -2.21127
H  -2.21938  1.00585 -2.64301
Si -1.77992 -2.18319 -2.26378
C  -0.63480 -1.61986 -3.70492
H  -0.95224 -0.64018 -4.10249
H  -0.66823 -2.35340 -4.53317
H   0.41657 -1.52947 -3.38564
C  -1.20388 -3.98154 -1.88335
H  -0.20444 -3.98248 -1.41547
H  -1.14251 -4.58699 -2.80779
H  -1.90282 -4.48265 -1.19156
C  -3.53086 -2.33152 -3.03827
H  -4.25563 -2.74741 -2.31908
H  -3.50774 -2.98264 -3.93212
H  -3.90644 -1.33843 -3.33799
```

3-Mg (anion) (based on **3-Ca**)

SCF (BP86) Energy = -1585.08452764
Enthalpy 0K = -1584.238299
Enthalpy 298K = -1584.176861
Free Energy 298K = -1584.335033
Lowest Frequency = 13.5022 cm^{-1}
Second Frequency = 19.9165 cm^{-1}
SCF (BP86-D3BJ) Energy = -1585.35709040
SCF (THF) Energy = -1585.13397866
SCF (BS2) Energy = -2641.60944177

```
Mg  0.18728 -0.21822 -0.61020
N  -1.58409 -1.30090 -0.72648
Si -2.14238 -1.58375 -2.36481
Si  1.47018  2.71493 -1.66393
N   0.16432  1.84351 -0.85381
Si  2.84918 -2.40113 -1.21486
N   2.05181 -1.13224 -0.27737
C  -3.13783 -3.53614  1.90474
H  -3.02531 -4.56701  2.27045
C  -4.16845 -1.37967  2.02401
H  -4.88779 -0.68513  2.48037
C  -1.30379 -4.08472  0.28549
H  -1.73471 -4.72372 -0.50735
H  -0.92305 -4.75799  1.07415
H  -0.46350 -3.54075 -0.16697
C  -4.99197 -3.18995  3.61752
H  -5.99013 -3.48594  3.23876
H  -5.16015 -2.41122  4.38291
H  -4.56448 -4.07267  4.12453
C  -3.49425  0.53680  0.55396
H  -3.60359  0.63940 -0.53620
H  -2.60360  1.13106  0.82319
H  -4.36369  1.01050  1.04060
C  -2.34003 -3.41919 -2.90125
H  -3.10717 -3.92799 -2.29191
H  -1.39995 -3.98721 -2.80921
H  -2.66834 -3.46819 -3.95615
C  -3.85719 -0.82674 -2.78800
H  -4.61203 -1.15003 -2.04997
H  -4.19535 -1.16724 -3.78402
H  -3.84253  0.27557 -2.79314
C  -0.83645 -0.79746 -3.52824
H  -0.61321  0.24851 -3.24783
H  -1.21632 -0.77923 -4.56540
H   0.11073 -1.36624 -3.54389
C  -1.54387  3.86902  1.83551
H  -1.41074  4.08598  2.90477
C  -2.76619  4.12675 -0.21091
H  -3.61586  4.54079 -0.77223
C  -3.54871  5.44102  1.82669
H  -3.61282  5.24838  2.91199
H  -4.57091  5.37762  1.41306
H  -3.21781  6.49136  1.70457
C  -2.17392  2.86323 -2.30534
H  -1.41095  3.22906 -3.01181
H  -3.15367  3.25602 -2.62907
H  -2.18357  1.76481 -2.40474
C   2.25362  1.55803 -2.96244
H   2.47383  0.56996 -2.52591
H   3.20146  1.97285 -3.34934
H   1.57205  1.41148 -3.81832
C   2.84411  3.35380 -0.48433
H   2.44274  4.14530  0.17349
H   3.68005  3.79396 -1.05949
H   3.24972  2.55493  0.15727
C   0.93061  4.31071 -2.59517
H   0.21502  4.90671 -2.00387
H   0.46869  4.09252 -3.57316
H   1.81916  4.94134 -2.78130
C   2.56756 -0.54123  3.40285
H   2.02732 -0.75779  4.33515
C   4.46259  0.35077  2.24674
H   5.43295  0.86752  2.25408
C   0.69103 -1.71859  2.22067
H  -0.17853 -1.13758  1.84939
H   0.42986 -2.01228  3.25143
H   0.73516 -2.62129  1.59047
C   4.37737  0.60810  4.78099
H   4.11504 -0.07147  5.61151
H   3.99897  1.61178  5.05694
H   5.47860  0.67958  4.73994
C   4.74242  0.24303 -0.23950
H   5.29546 -0.64383 -0.60183
H   5.48936  1.03064 -0.04038
H   4.09684  0.57456 -1.06706
C   1.60841 -3.71525 -1.84603
H   1.30540 -4.40414 -1.04115
H   2.07031 -4.31393 -2.65217
H   0.69163 -3.25677 -2.25167
C   4.11935 -3.35578 -0.14381
H   3.60965 -3.82584  0.71495
H   4.91435 -2.71008  0.26217
H   4.59492 -4.15725 -0.73728
C   3.72813 -1.87254 -2.84728
H   2.99964 -1.47695 -3.57693
H   4.19693 -2.76351 -3.30571
H   4.50999 -1.10935 -2.71307
C   3.80207  0.12658  3.46512
C   1.99840 -0.96138  2.18810
C   2.66408 -0.72020  0.93732
C   3.93415 -0.05250  1.00503
C  -1.88330  3.25673 -0.87552
C  -2.61071  4.46635  1.14447
C  -0.63304  2.99413  1.21164
C  -0.76186  2.69131 -0.18310
C  -3.35963 -0.90519  0.97571
C  -2.40717 -1.77170  0.33916
C  -2.31165 -3.10916  0.84643
C  -4.08599 -2.69704  2.50766
C   0.44983  2.34773  2.04454
H   0.27471  1.26362  2.17932
H   1.44538  2.43253  1.58468
H   0.48799  2.79168  3.05411
```

3-Mg (dianion) (based on **3-Ca**)

SCF (BP86) Energy = -1584.96123996

Enthalpy 0K = -1584.122095
Enthalpy 298K = -1584.060160
Free Energy 298K = -1584.218923
Lowest Frequency = 18.2517 cm^{-1}
Second Frequency = 20.8498 cm^{-1}
SCF (BP86-D3BJ) Energy = -1585.23138099
SCF (THF) Energy = -1585.12818968
SCF (BS2) Energy = -2641.51397589

```
Mg  0.29437 -0.27376 -0.17692
N  -1.59399 -1.21810 -0.70807
Si -2.00014 -1.42819 -2.39341
Si  1.98397  2.35887 -1.58641
N   0.50024  1.79874 -0.82653
Si  2.83850 -2.87559 -0.85451
N   2.08941 -1.46218 -0.18783
C  -3.77905 -3.26931  1.60331
H  -3.89765 -4.32273  1.90049
C  -4.38425 -0.96043  1.80615
H  -4.99954 -0.16626  2.25510
C  -1.91327 -4.07101  0.13309
H  -2.45213 -4.73273 -0.57165
H  -1.54811 -4.70414  0.96259
H  -1.04809 -3.65040 -0.39988
C  -5.69185 -2.66694  3.17805
H  -6.60784 -3.05209  2.68267
H  -5.99844 -1.79545  3.78433
H  -5.35773 -3.45694  3.87731
C  -3.23785  0.86480  0.51195
H  -3.33869  1.03856 -0.57254
H  -2.23968  1.25108  0.78085
H  -3.98494  1.48708  1.03572
C  -2.28986 -3.22471 -3.04585
H  -3.16937 -3.69646 -2.57379
H  -1.41602 -3.87275 -2.86647
H  -2.47418 -3.19171 -4.13736
C  -3.64547 -0.55580 -2.90452
H  -4.45276 -0.84426 -2.20790
H  -3.95608 -0.85348 -3.92427
H  -3.56202  0.54340 -2.88072
C  -0.57107 -0.72920 -3.45305
H  -0.20645  0.22774 -3.04085
H  -0.90323 -0.55285 -4.49268
H   0.28194 -1.42852 -3.48590
C  -1.15948  4.38732  1.37220
H  -1.07956  4.75785  2.40543
C  -2.19848  4.43497 -0.78643
H  -2.95305  4.83452 -1.48090
C  -2.99944  6.10492  0.96716
H  -3.01531  6.18791  2.06888
H  -4.04528  5.97374  0.62911
H  -2.66370  7.08781  0.57484
C  -1.56984  2.83508 -2.61789
H  -0.68650  2.96707 -3.26477
H  -2.43445  3.31586 -3.11158
H  -1.75032  1.74913 -2.56930
C   2.73368  0.95392 -2.63257
H   2.74861  0.02882 -2.02635
H   3.76963  1.19258 -2.93573
H   2.13855  0.76625 -3.54307
C   3.35475  2.96790 -0.37998
H   3.06672  3.92165  0.09833
H   4.30348  3.14165 -0.92396
H   3.53965  2.22692  0.41632
C   1.75058  3.87875 -2.76349
H   1.07384  4.63116 -2.32239
H   1.33956  3.58725 -3.74624
H   2.72790  4.36599 -2.94196
C   1.53022 -0.26711  3.33720
H   0.71614 -0.25847  4.07944
C   3.81654  0.25740  2.74056
H   4.79041  0.70163  2.99101
C   0.37338 -2.15341  2.16067
H   0.14992 -2.58603  1.17340
H  -0.58923 -1.91745  2.64801
H   0.85330 -2.96519  2.76700
C   2.93510  1.02463  5.00223
H   2.06593  0.86619  5.66638
H   3.06224  2.12045  4.88280
H   3.83779  0.66452  5.53786
C   4.81631 -0.33932  0.50167
H   5.45963 -1.24313  0.55949
H   5.47108  0.52940  0.71098
H   4.46605 -0.26433 -0.54090
C   1.57257 -3.94646 -1.82729
H   0.81573 -4.37615 -1.15055
H   2.07326 -4.77794 -2.35866
H   1.03777 -3.34166 -2.57967
C   3.59796 -4.00751  0.50255
H   2.80213 -4.40164  1.15855
H   4.28760 -3.42899  1.14022
H   4.15100 -4.86278  0.06983
C   4.24588 -2.61768 -2.16316
H   3.91378 -1.93627 -2.96627
H   4.52420 -3.58253 -2.63056
H   5.15454 -2.18232 -1.71497
C   2.74827  0.31957  3.67232
C   1.28379 -0.92311  2.06376
C   2.41530 -0.96916  1.11222
C   3.64833 -0.37527  1.46576
C  -1.36141  3.39587 -1.23086
C  -2.11230  4.96292  0.51588
C  -0.29474  3.34896  0.97174
C  -0.35510  2.83288 -0.37109
C  -3.40454 -0.59311  0.86632
C  -2.58006 -1.59177  0.23538
C  -2.78669 -2.95078  0.65613
C  -4.60568 -2.29488  2.18964
C   0.67772  2.78379  1.97875
H   0.59483  1.68735  2.08137
H   1.72946  2.96330  1.69893
H   0.50955  3.23305  2.97417
```

3-Ca
SCF (BP86) Energy = -1649.39893086
Enthalpy 0K = -1648.554307
Enthalpy 298K = -1648.489678
Free Energy 298K = -1648.656923
Lowest Frequency = 14.6138 cm^{-1}
Second Frequency = 19.1752 cm^{-1}
SCF (BP86-D3BJ) Energy = -1649.67679332
SCF (THF) Energy = -1649.43999727
SCF (BS2) Energy = -3719.14306129

```
Ca  0.61176 -0.17518 -0.34303
N  -1.72591 -0.71684 -0.66603
K  -5.49780 -1.15992 -0.62119
Si -2.10328 -0.77781 -2.36271
Si  2.66778  2.40386 -1.72504
N   1.17421  1.99584 -0.89362
Si  2.52706 -3.39624 -1.04703
N   2.08000 -1.95223 -0.16029
C  -4.02097 -2.41188  1.79714
H  -4.25083 -3.43152  2.13720
C  -4.30069 -0.03075  1.98418
H  -4.76217  0.84320  2.46472
```

```
C  -2.34896 -3.47051  0.23486
H  -2.84726 -3.87482 -0.66543
H  -2.32233 -4.28042  0.98288
H  -1.32079 -3.22236 -0.06922
C  -5.67012 -1.52845  3.53728
H  -6.43757 -0.73511  3.55995
H  -5.16483 -1.50903  4.52065
H  -6.18440 -2.50210  3.46201
C  -2.95100  1.60763  0.62335
H  -2.94084  1.76975 -0.46550
H  -1.93036  1.86609  0.96089
H  -3.63578  2.33536  1.08882
C  -2.89952 -2.41395 -3.01000
H  -3.84956 -2.69746 -2.51330
H  -2.21191 -3.26501 -2.87530
H  -3.12004 -2.32194 -4.08900
C  -3.32823  0.59394 -2.95685
H  -4.27380  0.65742 -2.38057
H  -3.60642  0.44086 -4.01542
H  -2.85625  1.58668 -2.87103
C  -0.48140 -0.53280 -3.32449
H   0.02352  0.40942 -3.03668
H  -0.67814 -0.46873 -4.40850
H   0.21703 -1.37589 -3.17497
C  -0.28029  4.11492  1.85666
H  -0.16373  4.22985  2.94320
C  -1.32301  4.73820 -0.20919
H  -2.04899  5.34485 -0.76937
C  -2.00666  5.98920  1.90403
H  -2.24358  5.68298  2.93800
H  -2.95906  6.20108  1.38735
H  -1.45819  6.94842  1.97204
C  -0.77974  3.64220 -2.40306
H  -0.11394  4.29270 -2.99792
H  -1.81666  3.90948 -2.67527
H  -0.56820  2.60843 -2.72214
C   3.09081  0.92753 -2.86313
H   3.00934 -0.04101 -2.33625
H   4.12635  1.00354 -3.23895
H   2.41961  0.89651 -3.73912
C   4.15261  2.69253 -0.54648
H   3.98674  3.58410  0.08391
H   5.08390  2.86096 -1.11767
H   4.31446  1.83049  0.12280
C   2.60292  3.97842 -2.81779
H   2.19854  4.83865 -2.25675
H   1.98340  3.83442 -3.71865
H   3.62163  4.24818 -3.14977
C   2.30931 -1.03598  3.47026
H   1.65217 -1.05010  4.35061
C   4.43889 -0.60131  2.45830
H   5.47546 -0.24489  2.53297
C   0.43097 -2.16485  2.22498
H  -0.35531 -1.57740  1.70439
H   0.05594 -2.30841  3.25247
H   0.45673 -3.13978  1.70992
C   4.13622 -0.00598  4.91731
H   3.41874 -0.18259  5.73624
H   4.31661  1.08401  4.86602
H   5.09501 -0.47412  5.20492
C   4.94993 -1.11596  0.05214
H   5.23014 -2.14880 -0.22441
H   5.88045 -0.57552  0.29249
H   4.50406 -0.66303 -0.84855
C   0.93416 -4.27401 -1.65675
H   0.36195 -4.69145 -0.81081
H   1.17306 -5.10246 -2.34765
H   0.27260 -3.57255 -2.19615
C   3.47244 -4.64875  0.04748
H   2.85831 -4.95168  0.91308
H   4.41190 -4.22785  0.44316
H   3.72481 -5.55947 -0.52384
C   3.54035 -3.09430 -2.64758
H   3.02141 -2.38586 -3.31739
H   3.67318 -4.03994 -3.20413
H   4.54070 -2.67956 -2.44380
C   3.61773 -0.55217  3.60362
C   1.81149 -1.53635  2.24813
C   2.63669 -1.55628  1.06832
C   3.99204 -1.08677  1.22028
C  -0.55208  3.79638 -0.91574
C  -1.20204  4.93118  1.17756
C   0.51213  3.15806  1.19544
C   0.41136  2.98039 -0.22873
C  -3.32331  0.19206  0.99114
C  -2.67693 -0.92412  0.33228
C  -3.04004 -2.24642  0.79348
C  -4.67736 -1.32441  2.41020
C   1.48823  2.33303  2.00720
H   1.22943  1.25570  2.06472
H   2.50724  2.36996  1.59158
H   1.52398  2.68696  3.05130
```

3-Ca (anion)

SCF (BP86) Energy = -1621.11408483
Enthalpy 0K = -1620.270692
Enthalpy 298K = -1620.207989
Free Energy 298K = -1620.372126
Lowest Frequency = 9.8094 cm^{-1}
Second Frequency = 19.2929 cm^{-1}
SCF (BP86-D3BJ) Energy = -1621.38052769
SCF (THF) Energy = -1621.16404168
SCF (BS2) Energy = -3119.21696465

```
Ca  0.27711 -0.23031 -0.56260
N  -1.72686 -1.41996 -0.78968
Si -2.06701 -1.68687 -2.47513
Si  1.64097  2.80813 -1.76357
N   0.29935  2.08226 -0.90530
Si  3.14991 -2.67106 -0.90789
N   2.28003 -1.38389 -0.10652
C  -3.43107 -3.51898  1.83905
H  -3.37302 -4.54312  2.23548
C  -4.35998 -1.31035  1.88915
H  -5.04998 -0.57107  2.32011
C  -1.60716 -4.19487  0.24527
H  -2.02118 -4.75159 -0.61555
H  -1.35239 -4.93757  1.02177
H  -0.68462 -3.71045 -0.11194
C  -5.28087 -3.03587  3.52366
H  -6.28740 -3.30241  3.14443
H  -5.42369 -2.22683  4.26243
H  -4.89627 -3.91886  4.06374
C  -3.58570  0.52460  0.35223
H  -3.79019  0.58334 -0.72974
H  -2.63648  1.06873  0.50683
H  -4.37215  1.08623  0.88413
C  -2.15768 -3.50381 -3.09687
H  -3.01018 -4.03924 -2.64361
H  -1.23930 -4.06552 -2.85798
H  -2.29795 -3.52501 -4.19372
C  -3.69107 -0.88876 -3.11680
H  -4.54797 -1.22318 -2.50582
H  -3.89354 -1.17597 -4.16496
H  -3.65365  0.21232 -3.06543
C  -0.60254 -0.87885 -3.42740
```

H	-0.48662	0.19866	-3.20027
H	-0.78202	-0.94734	-4.51488
H	0.36096	-1.39337	-3.24541
C	-1.53760	4.07501	1.70905
H	-1.42562	4.32650	2.77335
C	-2.76557	4.19651	-0.34648
H	-3.63860	4.53742	-0.92118
C	-3.65890	5.48631	1.66162
H	-3.74415	5.28275	2.74367
H	-4.66287	5.36533	1.21753
H	-3.38803	6.55576	1.55626
C	-2.06224	2.95125	-2.41781
H	-1.29368	3.32919	-3.11211
H	-3.04614	3.30593	-2.77131
H	-2.03820	1.85023	-2.50151
C	2.45705	1.43121	-2.81141
H	2.66665	0.52614	-2.21075
H	3.42323	1.77201	-3.22452
H	1.81270	1.14106	-3.65962
C	3.00718	3.56933	-0.64590
H	2.61689	4.43577	-0.08281
H	3.86036	3.92572	-1.25298
H	3.38861	2.83558	0.08388
C	1.18043	4.24152	-2.96195
H	0.50136	4.96263	-2.47462
H	0.68572	3.87484	-3.87777
H	2.08870	4.79176	-3.26883
C	2.09265	-0.43330	3.51677
H	1.42535	-0.62333	4.36909
C	4.06698	0.56063	2.59053
H	4.96632	1.18317	2.70194
C	0.62652	-2.00776	2.20718
H	-0.27468	-1.62701	1.68374
H	0.27755	-2.27416	3.21926
H	0.90931	-2.92833	1.67014
C	3.49602	1.06840	5.02260
H	2.99211	0.54937	5.85662
H	3.14009	2.11682	5.03191
H	4.57750	1.09607	5.24775
C	4.79568	0.20232	0.21264
H	5.36525	-0.71276	-0.03480
H	5.52767	0.98332	0.48071
H	4.28042	0.50680	-0.71307
C	1.90959	-3.96363	-1.58560
H	1.39469	-4.48962	-0.76432
H	2.41884	-4.71866	-2.21217
H	1.13197	-3.48606	-2.20744
C	4.33149	-3.59757	0.28519
H	3.76324	-4.05207	1.11506
H	5.08102	-2.92285	0.73220
H	4.87125	-4.40596	-0.23989
C	4.16600	-2.15344	-2.45799
H	3.52449	-1.62997	-3.18888
H	4.58221	-3.04584	-2.96124
H	5.00370	-1.47960	-2.21494
C	3.21648	0.38710	3.69907
C	1.79634	-1.04674	2.28338
C	2.64428	-0.84135	1.13700
C	3.81609	-0.02802	1.34047
C	-1.81717	3.38109	-0.98970
C	-2.64555	4.57319	1.00244
C	-0.55973	3.26044	1.10832
C	-0.66003	2.91105	-0.28077
C	-3.52116	-0.90476	0.83684
C	-2.60514	-1.83495	0.23594
C	-2.57515	-3.16317	0.77898
C	-4.34327	-2.61740	2.40946
C	0.58719	2.74789	1.95080
H	0.61870	1.64344	2.00677
H	1.56811	3.04888	1.54996
H	0.50707	3.12094	2.98637

3-Ca (dianion)

SCF (BP86) Energy = -1621.00201669
Enthalpy 0K = -1620.165484
Enthalpy 298K = -1620.102223
Free Energy 298K = -1620.267681
Lowest Frequency = 11.6354 cm^{-1}
Second Frequency = 17.0764 cm^{-1}
SCF (BP86-D3BJ) Energy = -1621.26575554
SCF (THF) Energy = -1621.16734923
SCF (BS2) Energy = -3119.13471923

Ca	0.30105	-0.28741	-0.20023
N	-1.84630	-1.27913	-0.74715
Si	-2.24517	-1.52001	-2.42062
Si	2.00653	2.50047	-1.74228
N	0.56215	2.03804	-0.88472
Si	2.86901	-3.15422	-0.76975
N	2.18642	-1.71530	-0.11100
C	-3.90860	-3.16191	1.79251
H	-4.01111	-4.19149	2.16939
C	-4.52135	-0.84524	1.85718
H	-5.11643	-0.02219	2.28018
C	-2.11722	-4.06170	0.27530
H	-2.64590	-4.68695	-0.46971
H	-1.81992	-4.72772	1.10676
H	-1.21070	-3.67597	-0.21727
C	-5.74292	-2.45496	3.41430
H	-6.78170	-2.42403	3.02442
H	-5.70026	-1.72822	4.24793
H	-5.59018	-3.46131	3.84452
C	-3.42522	0.88947	0.40196
H	-3.54000	0.99143	-0.68995
H	-2.42021	1.28706	0.63651
H	-4.16357	1.54561	0.89607
C	-2.58488	-3.33055	-3.00419
H	-3.47022	-3.75821	-2.50105
H	-1.72482	-3.98868	-2.79425
H	-2.77598	-3.35107	-4.09473
C	-3.82957	-0.57971	-2.99874
H	-4.67500	-0.80006	-2.32257
H	-4.12726	-0.87830	-4.02221
H	-3.67454	0.51288	-2.99427
C	-0.78699	-0.90338	-3.49486
H	-0.51984	0.14144	-3.25761
H	-1.05079	-0.95103	-4.56914
H	0.11550	-1.52236	-3.34223
C	-1.02540	4.50541	1.47474
H	-0.89883	4.84362	2.51424
C	-2.19287	4.58500	-0.61801
H	-3.00110	4.98215	-1.25114
C	-2.92201	6.19164	1.22220
H	-3.11689	6.07317	2.30421
H	-3.90083	6.21346	0.70870
H	-2.46991	7.19716	1.09028
C	-1.63237	3.04306	-2.52936
H	-0.80131	3.22948	-3.23063
H	-2.54909	3.49213	-2.95381
H	-1.75988	1.94639	-2.49403
C	2.63467	0.99332	-2.73387
H	2.67014	0.08498	-2.10132
H	3.65491	1.17093	-3.12294
H	1.97164	0.78084	-3.59069
C	3.46732	3.10388	-0.64091
H	3.22023	4.06070	-0.14574

```
H   4.38061  3.26678 -1.24592
H   3.69841  2.36511  0.14634
C   1.77322  3.95382 -2.99964
H   1.17015  4.76489 -2.55391
H   1.26416  3.62329 -3.92243
H   2.75192  4.38091 -3.28980
C   1.83973 -0.32945  3.37925
H   1.09960 -0.34273  4.19488
C   3.99648  0.33754  2.51680
H   4.96141  0.84691  2.65021
C   0.48181 -2.14490  2.27263
H  -0.07599 -2.35647  1.34374
H  -0.26560 -1.91326  3.05155
H   0.96217 -3.12274  2.54203
C   3.30629  1.12957  4.84368
H   2.50432  0.97235  5.58775
H   3.37985  2.22354  4.66675
H   4.26525  0.83007  5.31712
C   4.84415 -0.40202  0.26587
H   5.42504 -1.34638  0.27162
H   5.56124  0.42476  0.42512
H   4.41538 -0.30757 -0.74581
C   1.49380 -4.24328 -1.55176
H   0.83159 -4.65514 -0.77103
H   1.90753 -5.08575 -2.13772
H   0.86603 -3.63547 -2.22763
C   3.76607 -4.23338  0.54888
H   3.04602 -4.56740  1.31707
H   4.54541 -3.64672  1.06576
H   4.24156 -5.12835  0.10465
C   4.13152 -2.92003 -2.21749
H   3.69218 -2.28155 -3.00505
H   4.40009 -3.89047 -2.67843
H   5.06210 -2.43312 -1.87917
C   3.03314  0.35914  3.57020
C   1.51280 -1.02783  2.16932
C   2.53975 -1.09554  1.11157
C   3.75895 -0.38475  1.32215
C  -1.35674  3.58439 -1.14599
C  -2.04104  5.08032  0.68856
C  -0.16498  3.50221  0.99293
C  -0.28319  3.03038 -0.36315
C  -3.58462 -0.54346  0.85469
C  -2.78339 -1.58227  0.25768
C  -2.96533 -2.91279  0.77595
C  -4.71120 -2.15318  2.34638
C   0.86698  2.90441  1.92066
H   0.72796  1.81256  2.04384
H   1.89640  3.02592  1.54507
H   0.80634  3.36631  2.92267
```

3-Sr

SCF (BP86) Energy = -1643.29921168
Enthalpy 0K = -1642.454725
Enthalpy 298K = -1642.390020
Free Energy 298K = -1642.559780
Lowest Frequency = 11.1838 cm^{-1}
Second Frequency = 15.7016 cm^{-1}
SCF (BP86-D3BJ) Energy = -1643.57128358
SCF (THF) Energy = -1643.34411522
SCF (BS2) Energy = -3072.22526612

```
Sr -0.51227 -0.37701 -0.32875
K   1.25173  5.46161  1.12832
Si -1.52650  3.27214 -1.29243
Si -1.38685 -2.06618  3.02827
Si  0.97502 -1.90621 -3.02650
N  -0.40545  2.24602 -0.43584
N  -1.83103 -1.28932  1.53316
N   1.32268 -1.66333 -1.33798
C  -1.64491  3.00309 -3.18224
H  -0.68789  3.15860 -3.70612
H  -2.39213  3.68761 -3.62250
H  -1.98140  1.97466 -3.40167
C  -1.13744  5.15673 -1.02455
H  -1.30058  5.44466  0.03359
H  -1.84047  5.76849 -1.61588
H  -0.12037  5.44082 -1.35653
C  -3.28527  3.02796 -0.61041
H  -3.58825  1.96663 -0.63752
H  -4.02639  3.60231 -1.19460
H  -3.35120  3.35205  0.44253
C   0.79638  2.60652  0.13770
C   1.95223  3.05670 -0.61295
C   3.16682  3.34929  0.04327
H   4.03111  3.64217 -0.56898
C   3.33695  3.21397  1.43986
C   2.21131  2.78707  2.18032
H   2.30607  2.66303  3.26829
C   0.96972  2.49715  1.57663
C   1.88363  3.13349 -2.12064
H   1.18722  3.91002 -2.48570
H   1.51244  2.17803 -2.53214
H   2.87640  3.33774 -2.55384
C   4.68474  3.42717  2.09917
H   5.29795  4.16723  1.55616
H   5.26530  2.48673  2.12369
H   4.58384  3.76554  3.14531
C  -0.21507  2.11867  2.43792
H   0.02125  2.22715  3.51021
H  -0.55824  1.07520  2.28861
H  -1.09920  2.73550  2.19308
C   0.50260 -1.83736  3.20362
H   1.05940 -2.25565  2.34640
H   0.87232 -2.34940  4.10968
H   0.77533 -0.77108  3.30269
C  -1.78597 -3.94082  3.07161
H  -2.85579 -4.12091  2.86506
H  -1.56185 -4.37364  4.06338
H  -1.19710 -4.49564  2.32166
C  -2.16747 -1.36105  4.63788
H  -2.00998 -0.27316  4.73386
H  -1.71165 -1.84680  5.52025
H  -3.25405 -1.55075  4.68506
C  -3.05436 -1.22531  0.85832
C  -3.29958 -2.06432 -0.29120
C  -4.45876 -1.88797 -1.07743
H  -4.61486 -2.55611 -1.93552
C  -5.43220 -0.92262 -0.77995
C  -5.21375 -0.12627  0.36184
H  -5.96437  0.62853  0.63486
C  -4.07238 -0.25113  1.17072
C  -2.38322 -3.23350 -0.60163
H  -2.31035 -3.91326  0.26451
H  -1.33561 -2.96692 -0.84488
H  -2.77055 -3.80694 -1.46075
C  -6.66219 -0.73950 -1.64434
H  -6.60776  0.18453 -2.25081
H  -7.58133 -0.66721 -1.03577
H  -6.78936 -1.58236 -2.34431
C  -3.92458  0.64161  2.37981
H  -4.62363  1.49367  2.33168
H  -2.89354  1.02169  2.46579
H  -4.12762  0.09511  3.31779
C   2.21765 -1.17769 -4.29526
H   2.32993 -0.08588 -4.18168
```

```
H   1.88131 -1.37955 -5.32866
H   3.21591 -1.63393 -4.17766
C   0.70702 -3.71822 -3.59061
H   1.59652 -4.33232 -3.36660
H   0.53197 -3.77084 -4.68048
H  -0.15745 -4.18094 -3.08515
C  -0.68894 -0.97768 -3.35806
H  -1.56060 -1.37272 -2.79851
H  -0.95290 -1.09987 -4.42321
H  -0.60892  0.11550 -3.19822
C   2.46699 -2.18953 -0.69314
C   2.42928 -3.45342 -0.01317
C   3.56651 -3.93372  0.66081
H   3.50174 -4.90923  1.16222
C   4.77374 -3.21556  0.71540
C   4.80488 -1.97288  0.06244
H   5.73059 -1.38064  0.08610
C   3.69479 -1.45229 -0.62870
C   1.16669 -4.28500  0.00175
H   0.32991 -3.74268  0.47762
H   1.32290 -5.22220  0.56250
H   0.82727 -4.54695 -1.01423
C   5.97317 -3.74417  1.47473
H   5.88717 -3.56205  2.56345
H   6.90757 -3.26290  1.13820
H   6.09086 -4.83430  1.34273
C   3.81634 -0.10104 -1.29421
H   3.84394 -0.17860 -2.39521
H   4.73815  0.41312 -0.97174
H   2.95533  0.54483 -1.05157
```

3-Sr (anion)

SCF (BP86) Energy = -1615.01788866
Enthalpy 0K = -1614.175019
Enthalpy 298K = -1614.112094
Free Energy 298K = -1614.279026
Lowest Frequency = 9.4555 cm^{-1}
Second Frequency = 16.4693 cm^{-1}
SCF (BP86-D3BJ) Energy = -1615.27967757
SCF (THF) Energy = -1615.06773865
SCF (BS2) Energy = -2472.30205773

```
Sr  0.07046  0.11495  0.09831
Si  1.75647  3.44538  1.16153
Si  1.23755 -1.71605 -2.63336
Si -1.39591 -1.32475  2.96598
N   0.45113  2.55891  0.43706
N   1.84729 -1.12715 -1.11841
N  -1.71180 -1.21060  1.26496
C   1.28628  4.64263  2.59625
H   0.61867  5.45190  2.25088
H   2.19376  5.11867  3.01263
H   0.77750  4.11096  3.41866
C   2.74181  4.55505 -0.06342
H   3.25914  3.94793 -0.82588
H   3.50563  5.15326  0.46717
H   2.07214  5.26022 -0.58728
C   2.97314  2.17494  1.90429
H   2.50662  1.60215  2.72491
H   3.85717  2.68758  2.32439
H   3.33980  1.45569  1.15093
C  -0.64216  3.06224 -0.27651
C  -1.86968  3.44758  0.37500
C  -2.98081  3.85969 -0.38049
H  -3.89743  4.14684  0.15388
C  -2.96790  3.90551 -1.78689
C  -1.77915  3.52057 -2.42812
H  -1.73065  3.55191 -3.52593
C  -0.63494  3.10750 -1.71789
C  -1.97170  3.40523  1.88240
H  -1.36700  4.19580  2.35968
H  -1.58819  2.44864  2.27927
H  -3.01756  3.53032  2.21058
C  -4.19656  4.31275 -2.57382
H  -4.74510  5.13357 -2.07770
H  -4.91072  3.47454 -2.68943
H  -3.92993  4.65270 -3.58981
C   0.62448  2.76970 -2.48894
H   0.43495  2.79560 -3.57596
H   1.03671  1.77292 -2.24354
H   1.43767  3.48189 -2.26452
C  -0.60316 -1.15602 -2.72256
H  -1.24420 -1.59674 -1.93477
H  -1.03572 -1.49759 -3.67973
H  -0.72731 -0.05443 -2.72716
C   1.26112 -3.62276 -2.86399
H   2.28412 -4.01777 -2.73295
H   0.92026 -3.90642 -3.87668
H   0.60696 -4.12519 -2.13197
C   2.06434 -1.03521 -4.23171
H   1.98388  0.06347 -4.29322
H   1.58174 -1.46214 -5.13058
H   3.13561 -1.29987 -4.27351
C   3.14167 -1.33794 -0.61346
C   3.42298 -2.42559  0.28260
C   4.71194 -2.59442  0.81992
H   4.88979 -3.44379  1.49513
C   5.77356 -1.72163  0.52594
C   5.49510 -0.64570 -0.33477
H   6.29767  0.06513 -0.57848
C   4.22296 -0.43432 -0.89673
C   2.33388 -3.40330  0.66186
H   1.95962 -3.96704 -0.21003
H   1.45012 -2.89325  1.08761
H   2.70054 -4.13010  1.40748
C   7.14379 -1.90436  1.14611
H   7.25330 -1.33667  2.09122
H   7.94558 -1.55662  0.47039
H   7.34232 -2.96443  1.38427
C   4.00116  0.76752 -1.78659
H   4.89618  1.41341 -1.80315
H   3.14445  1.37017 -1.43618
H   3.76929  0.48174 -2.82682
C  -2.66270 -0.46848  4.13262
H  -2.73003  0.61389  3.92828
H  -2.37570 -0.59758  5.19278
H  -3.67190 -0.89864  4.00622
C  -1.19507 -3.09160  3.69711
H  -2.10016 -3.69908  3.52072
H  -1.03028 -3.04849  4.78967
H  -0.33893 -3.62030  3.24475
C   0.28295 -0.42823  3.25078
H   1.13115 -0.93232  2.74819
H   0.52736 -0.42757  4.32774
H   0.25833  0.63623  2.94534
C  -2.81119 -1.78749  0.60419
C  -2.75154 -3.11468  0.05702
C  -3.84734 -3.64657 -0.64838
H  -3.76450 -4.66679 -1.05006
C  -5.03433 -2.92498 -0.85698
C  -5.08868 -1.62062 -0.33364
H  -5.99941 -1.02265 -0.48187
C  -4.01947 -1.04354  0.37343
C  -1.50485 -3.95356  0.22084
H  -0.59859 -3.38423 -0.04977
H  -1.55398 -4.85821 -0.41011
```

```
H  -1.35508 -4.28022  1.26486
C  -6.18821 -3.50821 -1.64706
H  -6.16228 -3.20324 -2.71191
H  -7.16411 -3.17983 -1.24603
H  -6.17293 -4.61228 -1.62883
C  -4.15071  0.37421  0.88036
H  -4.01290  0.43710  1.97253
H  -5.14418  0.78716  0.63315
H  -3.39254  1.04633  0.43841
```

3-Sr (dianion)

SCF (BP86) Energy = -1614.91866138
Enthalpy 0K = -1614.078983
Enthalpy 298K = -1614.015724
Free Energy 298K = -1614.183469
Lowest Frequency = 10.9660 cm^{-1}
Second Frequency = 18.3464 cm^{-1}
SCF (BP86-D3BJ) Energy = -1615.17915798
SCF (THF) Energy = -1615.08335381
SCF (BS2) Energy = -2472.21925330

```
Sr  0.25470 -0.16368 -0.98682
Si -0.67808 -3.80086 -1.66887
Si  1.96838  0.67116  2.21563
Si -2.36208  1.93109 -2.71238
N  -0.94095 -2.45311 -0.59340
N   2.27584  0.13004  0.58791
N  -1.47086  1.79078 -1.22196
C  -1.37972 -3.54568 -3.44155
H  -2.48082 -3.45448 -3.44346
H  -1.10350 -4.38814 -4.10529
H  -0.96366 -2.62061 -3.87992
C  -1.48934 -5.41738 -1.00380
H  -1.08348 -5.69626 -0.01591
H  -1.31338 -6.26035 -1.69769
H  -2.58007 -5.29356 -0.88524
C   1.18342 -4.16558 -1.95195
H   1.69194 -3.25963 -2.33512
H   1.32945 -4.96997 -2.69893
H   1.68795 -4.46554 -1.01676
C  -1.75684 -2.61164  0.53221
C  -3.16914 -2.33644  0.49495
C  -3.96237 -2.48295  1.64676
H  -5.03873 -2.26561  1.57343
C  -3.43095 -2.89434  2.88259
C  -2.05143 -3.16542  2.92662
H  -1.60095 -3.49417  3.87542
C  -1.22036 -3.03612  1.79946
C  -3.80339 -1.88661 -0.79983
H  -3.65439 -2.62383 -1.60910
H  -3.34542 -0.94625 -1.15612
H  -4.88850 -1.71995 -0.67405
C  -4.29763 -2.99826  4.12113
H  -5.33956 -3.26854  3.86736
H  -4.34492 -2.04483  4.68561
H  -3.91532 -3.76347  4.82197
C   0.25143 -3.35604  1.91101
H   0.52036 -3.63030  2.94716
H   0.87576 -2.49887  1.60112
H   0.54209 -4.19082  1.24669
C   0.09149  0.60973  2.55186
H  -0.46175  1.34285  1.93805
H  -0.10420  0.87557  3.60743
H  -0.34061 -0.39323  2.38613
C   2.52582  2.47681  2.60838
H   3.58817  2.63441  2.35052
H   2.40157  2.70327  3.68464
H   1.92264  3.20596  2.04009
C   2.81713 -0.36022  3.61381
H   2.38323 -1.37248  3.68761
H   2.68879  0.13454  4.59543
H   3.90102 -0.46652  3.43002
C   3.61262  0.04163  0.15476
C   4.27119  1.13990 -0.50345
C   5.62078  1.04440 -0.88419
H   6.08784  1.91308 -1.37245
C   6.38557 -0.11768 -0.67739
C   5.73036 -1.21329 -0.09058
H   6.28504 -2.15284  0.05511
C   4.38211 -1.16406  0.30904
C   3.49024  2.37980 -0.86278
H   2.90759  2.77497 -0.01701
H   2.75921  2.14533 -1.66593
H   4.15668  3.17524 -1.24366
C   7.83057 -0.20486 -1.12570
H   7.92584 -0.44168 -2.20520
H   8.37685 -0.99363 -0.57643
H   8.36931  0.74791 -0.96343
C   3.73652 -2.41180  0.86521
H   4.43626 -3.26655  0.82478
H   2.83440 -2.67422  0.28530
H   3.40410 -2.28895  1.90948
C  -4.27039  2.14388 -2.48499
H  -4.73457  1.22101 -2.09563
H  -4.75875  2.38815 -3.44781
H  -4.49577  2.96100 -1.77629
C  -1.89748  3.41582 -3.85509
H  -2.08233  4.38665 -3.36129
H  -2.50121  3.38998 -4.78292
H  -0.83184  3.37028 -4.13782
C  -2.08635  0.36372 -3.76162
H  -1.01090  0.22959 -3.99555
H  -2.62912  0.44202 -4.72314
H  -2.43546 -0.54933 -3.25011
C  -1.58335  2.79971 -0.25351
C  -0.74827  3.97454 -0.25485
C  -0.87695  4.94735  0.75464
H  -0.21900  5.82903  0.71603
C  -1.80672  4.83723  1.80217
C  -2.61338  3.68483  1.81831
H  -3.34440  3.55475  2.63051
C  -2.51612  2.67935  0.84081
C   0.28368  4.18795 -1.34036
H   0.59793  3.22522 -1.77678
H   1.17231  4.71303 -0.94732
H  -0.11249  4.79265 -2.17821
C  -1.89758  5.88215  2.89583
H  -1.26305  5.63461  3.77095
H  -2.93162  5.98805  3.27395
H  -1.57026  6.87499  2.53598
C  -3.39200  1.45347  0.95454
H  -4.05097  1.33245  0.07866
H  -4.02262  1.50376  1.86014
H  -2.79359  0.52710  1.01046
```

3-Ba·(THF)

SCF (BP86) Energy = -1870.54650611
Enthalpy 0K = -1869.588371
Enthalpy 298K = -1869.516174
Free Energy 298K = -1869.704376
Lowest Frequency = 12.6256 cm^{-1}
Second Frequency = 13.7497 cm^{-1}
SCF (BP86-D3BJ) Energy = -1870.85724951
SCF (THF) Energy = -1870.59149482
SCF (BS2) Energy = -3299.55142065

```
Ba -0.33081 -0.23354 -0.39145
Si  0.05845 -4.26281 -1.52407
Si  2.90873  0.71854 -2.61470
Si -2.71265  1.08224 -3.10425
O  -0.33763  0.69585  2.22398
N  -0.38316 -2.90162 -0.51715
N   2.28459  0.68306 -0.99506
N  -2.12788  1.34711 -1.48574
C  -1.11168 -3.07566  0.66533
C  -0.49082 -3.42837  1.91705
C  -1.25302 -3.51222  3.09777
H  -0.74569 -3.79612  4.03109
C  -2.63802 -3.26186  3.12406
C  -3.25145 -2.92358  1.90420
H  -4.33615 -2.75012  1.88289
C  -2.53378 -2.83660  0.69602
C   0.98914 -3.73603  1.96158
H   1.33963 -3.85753  3.00118
H   1.22748 -4.66741  1.41638
H   1.58524 -2.94480  1.47320
C  -3.44529 -3.41508  4.39749
H  -4.29440 -2.71063  4.42980
H  -3.86989 -4.43303  4.49301
H  -2.82697 -3.24079  5.29514
C  -3.27887 -2.58011 -0.59785
H  -3.01914 -3.33903 -1.35575
H  -4.36896 -2.60235 -0.43033
H  -3.04778 -1.59960 -1.05718
C  -0.49088 -4.00258 -3.34085
H  -1.58951 -3.94098 -3.42489
H  -0.07404 -3.07126 -3.76264
H  -0.14814 -4.83701 -3.97953
C  -0.74589 -5.88860 -0.91013
H  -0.42926 -6.13896  0.11686
H  -1.84647 -5.80809 -0.90206
H  -0.47324 -6.73543 -1.56445
C   1.95155 -4.57579 -1.64222
H   2.40324 -4.78194 -0.65706
H   2.16176 -5.43995 -2.29868
H   2.46913 -3.70201 -2.07567
C   2.95855  0.72624  0.20005
C   2.87649  1.90252  1.05045
C   3.51138  1.93341  2.30928
H   3.40934  2.83843  2.92557
C   4.25798  0.84324  2.81502
C   4.35459 -0.29987  1.98840
H   4.90475 -1.17686  2.35834
C   3.74088 -0.38142  0.71996
C   2.11564  3.11079  0.55708
H   2.37707  3.35417 -0.48753
H   1.02558  2.93392  0.54239
H   2.29682  3.99098  1.19798
C   4.85933  0.87019  4.20655
H   4.14578  0.49051  4.96160
H   5.76130  0.23766  4.27879
H   5.13353  1.89374  4.51731
C   3.85975 -1.65095 -0.08960
H   4.35773 -1.49006 -1.06330
H   4.42308 -2.42441  0.45826
H   2.86317 -2.06014 -0.33459
C   4.80549  1.14040 -2.66888
H   5.16223  1.11177 -3.71301
H   4.99897  2.17141 -2.30984
H   5.41984  0.40680 -2.11112
C   2.69345 -0.90095 -3.60315
H   3.27886 -1.73518 -3.18270
H   1.63485 -1.21514 -3.60992
H   2.99840 -0.76384 -4.65613
C   2.06528  2.08627 -3.64098
H   2.41138  2.07541 -4.68999
H   0.97054  1.94640 -3.64892
H   2.26115  3.08843 -3.22243
C  -2.52890  2.37836 -0.62594
C  -3.56106  2.17697  0.35967
C  -3.90886  3.20535  1.25473
H  -4.71562  3.01823  1.97744
C  -3.27054  4.45981  1.25432
C  -2.24442  4.65109  0.31246
H  -1.71898  5.61644  0.28605
C  -1.87002  3.65967 -0.61501
C  -4.28947  0.85366  0.44389
H  -3.62716  0.02564  0.75952
H  -5.11494  0.90837  1.17435
H  -4.70678  0.55519 -0.53101
C  -3.69606  5.56516  2.19867
H  -4.51721  6.17471  1.77445
H  -4.06106  5.16314  3.15993
H  -2.86347  6.25674  2.41627
C  -0.77354  3.96625 -1.61043
H  -1.18268  4.18463 -2.61270
H  -0.18654  4.84612 -1.29371
H  -0.09298  3.10822 -1.74165
C  -2.57044  2.56683 -4.31384
H  -3.01517  2.31096 -5.29279
H  -3.10829  3.45033 -3.92754
H  -1.52015  2.85738 -4.48673
C  -4.54479  0.52427 -3.23921
H  -4.72371 -0.42095 -2.69828
H  -5.22549  1.28718 -2.82207
H  -4.83112  0.36729 -4.29526
C  -1.63970 -0.33231 -3.82289
H  -1.98477 -0.60594 -4.83557
H  -0.57998 -0.03145 -3.91904
H  -1.69090 -1.25415 -3.21543
C  -0.75572  1.99680  2.71701
H  -1.30061  2.50839  1.90935
H   0.14943  2.57992  2.98704
C  -1.61066  1.68281  3.95660
H  -1.55213  2.48948  4.70530
H  -2.66565  1.57289  3.66056
C   0.02220 -0.05611  3.41276
H   0.00732 -1.12408  3.14592
H   1.04475  0.24094  3.72254
C  -1.03109  0.33100  4.47542
H  -0.57310  0.42393  5.47406
H  -1.81599 -0.43904  4.53361
K   5.80577  1.79445  0.31639
```

3-Ba·(THF) (anion)

SCF (BP86) Energy = -1842.26105113
Enthalpy 0K = -1841.305112
Enthalpy 298K = -1841.234275
Free Energy 298K = -1841.421636
Lowest Frequency = 11.1176 cm^{-1}
Second Frequency = 12.9503 cm^{-1}
SCF (BP86-D3BJ) Energy = -1842.56040585
SCF (THF) Energy = -1842.31212354
SCF (BS2) Energy = -2699.62435852

```
Ba  0.21376 -0.02037  0.42134
Si  4.17553  0.81529  1.42756
Si  0.12028 -3.56581  2.31767
Si -1.68203  1.80920  3.24977
O  -0.75696  0.06260 -2.16344
N   2.73483  0.99085  0.46274
N   0.08535 -2.67189  0.82915
```

N -1.82198 1.30525 1.59716
C 2.66389 1.87927 -0.61346
C 3.11083 1.52703 -1.93745
C 2.94820 2.42133 -3.01252
H 3.31040 2.11856 -4.00607
C 2.35766 3.68897 -2.85911
C 1.94343 4.04969 -1.56397
H 1.50756 5.04564 -1.40104
C 2.08652 3.19130 -0.45893
C 3.79058 0.19658 -2.17086
H 4.02112 0.05123 -3.24082
H 4.73742 0.11927 -1.60607
H 3.17180 -0.65058 -1.82757
C 2.16398 4.62059 -4.03806
H 1.15051 4.53575 -4.47702
H 2.29540 5.67802 -3.74610
H 2.88433 4.40397 -4.84659
C 1.68697 3.67154 0.91897
H 2.51305 3.54268 1.63996
H 1.39899 4.73686 0.89496
H 0.82952 3.10908 1.33354
C 3.82876 1.08800 3.29569
H 3.47828 2.11488 3.49819
H 3.05212 0.39387 3.66200
H 4.73945 0.91297 3.89811
C 5.53353 2.07455 0.92113
H 5.83525 1.94250 -0.13225
H 5.17209 3.11184 1.02927
H 6.43505 1.96094 1.54969
C 4.96835 -0.93288 1.35587
H 5.29179 -1.20161 0.33590
H 5.85052 -0.99127 2.02028
H 4.24981 -1.70133 1.69038
C 0.05626 -3.24126 -0.44468
C -1.17958 -3.58399 -1.10111
C -1.18107 -4.07493 -2.41880
H -2.14511 -4.33383 -2.88089
C 0.00345 -4.26245 -3.15728
C 1.21479 -3.95268 -2.51257
H 2.16126 -4.11787 -3.04756
C 1.26546 -3.46433 -1.19313
C -2.48429 -3.43628 -0.35373
H -2.50526 -4.06705 0.55339
H -2.64139 -2.40314 0.00167
H -3.34134 -3.72052 -0.98915
C -0.02786 -4.76512 -4.58645
H -0.22184 -3.95316 -5.31517
H 0.93329 -5.22792 -4.87148
H -0.82055 -5.52044 -4.73560
C 2.60391 -3.22488 -0.53285
H 2.72385 -3.85564 0.36713
H 3.43414 -3.45035 -1.22457
H 2.72758 -2.18497 -0.18118
C 0.25124 -5.46009 2.04147
H 0.27122 -6.00406 3.00289
H -0.60516 -5.83689 1.45594
H 1.16869 -5.71898 1.48522
C 1.60989 -3.03341 3.40657
H 2.56823 -3.27051 2.91321
H 1.59644 -1.94384 3.59273
H 1.59771 -3.53368 4.39260
C -1.41966 -3.25304 3.42271
H -1.31557 -3.73776 4.41119
H -1.56121 -2.17095 3.59510
H -2.34260 -3.63239 2.95151
C -2.93069 1.49334 0.76654
C -2.97094 2.57287 -0.18879
C -4.05380 2.70935 -1.07563
H -4.04937 3.55430 -1.77955
C -5.13880 1.81286 -1.09080
C -5.09782 0.74987 -0.17158
H -5.92260 0.02243 -0.16087
C -4.04264 0.57585 0.74418
C -1.84413 3.57958 -0.24314
H -0.88910 3.13166 -0.57441
H -2.08287 4.39599 -0.94718
H -1.64599 4.01908 0.74828
C -6.31297 2.00511 -2.02884
H -7.09885 2.64965 -1.58700
H -6.00420 2.48302 -2.97554
H -6.79338 1.04282 -2.27995
C -4.09370 -0.59233 1.70271
H -4.48899 -0.29444 2.69098
H -4.74504 -1.39569 1.31683
H -3.08675 -1.00189 1.88775
C -3.03252 1.20097 4.47870
H -2.83633 1.59795 5.49211
H -4.03745 1.54499 4.17669
H -3.05618 0.10009 4.54413
C -1.63852 3.71528 3.51628
H -0.78024 4.17506 2.99672
H -2.55876 4.18770 3.12877
H -1.56058 3.96863 4.58995
C -0.01790 1.11138 3.89117
H 0.16588 1.43610 4.93088
H -0.01826 0.00565 3.89845
H 0.84447 1.46074 3.29522
C -2.12804 -0.22359 -2.54429
H -2.78636 0.17718 -1.75839
H -2.25508 -1.32223 -2.62608
C -2.27589 0.46901 -3.90576
H -3.07803 0.02114 -4.51546
H -2.51748 1.53288 -3.74909
C 0.03444 -0.14735 -3.36475
H 0.95325 0.44941 -3.25056
H 0.29109 -1.22181 -3.43983
C -0.86596 0.30676 -4.54540
H -0.86442 -0.45075 -5.34696
H -0.51258 1.25522 -4.98109

3-Ba·(THF) (dianion)
SCF (BP86) Energy = -1842.15649285
Enthalpy 0K = -1841.202981
Enthalpy 298K = -1841.132521
Free Energy 298K = -1841.318015
Lowest Frequency = 10.6535 cm^{-1}
Second Frequency = 11.9887 cm^{-1}
SCF (BP86-D3BJ) Energy = -1842.45464434
SCF (THF) Energy = -1842.32107720
SCF (BS2) Energy = -2699.54106054

Ba 0.11904 -0.03119 -1.15782
Si 3.97417 0.69076 -1.95569
Si -2.09112 3.00055 -2.57944
Si -0.49040 -3.77349 -2.12220
O -0.37331 0.12320 1.70076
N 2.90465 0.16732 -0.67624
N -1.43414 2.31430 -1.11128
N -0.92060 -2.59377 -0.91267
C 3.40281 -0.15816 0.58196
C 3.87997 0.82554 1.52502
C 4.33580 0.44241 2.80081
H 4.68326 1.22751 3.48996
C 4.35828 -0.89685 3.22766
C 3.90483 -1.86485 2.30950
H 3.93070 -2.92682 2.59683

C 3.44052 -1.53196 1.02672
C 3.86106 2.29219 1.15824
H 4.04879 2.92513 2.04432
H 4.62167 2.54866 0.39759
H 2.89007 2.57545 0.71554
C 4.82523 -1.28093 4.61719
H 3.98360 -1.44240 5.32128
H 5.41104 -2.21961 4.60824
H 5.46432 -0.49451 5.05854
C 3.03145 -2.62785 0.07099
H 3.53144 -2.50241 -0.90604
H 3.28316 -3.62350 0.47913
H 1.94492 -2.62669 -0.13993
C 3.76897 -0.38503 -3.52654
H 4.02855 -1.44080 -3.32683
H 2.72569 -0.36103 -3.89508
H 4.41532 -0.01721 -4.34764
C 5.83328 0.53403 -1.46708
H 6.09067 1.11932 -0.56803
H 6.08377 -0.52032 -1.25392
H 6.48046 0.87461 -2.29661
C 3.67133 2.49118 -2.55569
H 3.84511 3.23631 -1.75999
H 4.32292 2.73984 -3.41525
H 2.62528 2.59775 -2.89729
C -1.63368 2.98138 0.09483
C -2.81327 2.77389 0.89882
C -2.98003 3.44453 2.12388
H -3.89977 3.26236 2.70100
C -2.02193 4.34230 2.63234
C -0.87274 4.55915 1.84870
H -0.11095 5.27004 2.20391
C -0.66675 3.91540 0.61508
C -3.88929 1.83356 0.40712
H -4.29692 2.15192 -0.56903
H -3.49580 0.81427 0.24506
H -4.72381 1.77238 1.12912
C -2.20546 5.02039 3.97484
H -1.80869 4.41650 4.81671
H -1.68409 5.99479 4.01134
H -3.27339 5.20551 4.19487
C 0.56071 4.23839 -0.20395
H 0.29488 4.75090 -1.14732
H 1.25753 4.88701 0.35667
H 1.10095 3.32713 -0.51612
C -3.12731 4.58629 -2.21307
H -3.53393 5.00778 -3.15103
H -3.97411 4.37938 -1.53564
H -2.50636 5.36260 -1.73199
C -0.76196 3.53122 -3.85786
H -0.09445 4.31499 -3.45513
H -0.13732 2.66128 -4.13562
H -1.23124 3.91588 -4.78519
C -3.23231 1.78655 -3.52871
H -3.58635 2.23173 -4.47909
H -2.67117 0.86728 -3.78402
H -4.11375 1.49400 -2.92980
C -1.91781 -2.87914 0.02265
C -1.61779 -3.48473 1.29816
C -2.64002 -3.75390 2.22658
H -2.36675 -4.22479 3.18329
C -3.98769 -3.42881 1.98599
C -4.27913 -2.78932 0.76702
H -5.31759 -2.49424 0.55434
C -3.29535 -2.50947 -0.19909
C -0.18309 -3.76973 1.67897
H 0.40880 -2.83676 1.70332
H -0.12754 -4.24020 2.67781
H 0.32057 -4.42969 0.95550
C -5.07634 -3.76843 2.98339
H -5.52185 -4.76908 2.80384
H -4.69046 -3.77451 4.01932
H -5.90654 -3.03983 2.94178
C -3.69137 -1.78793 -1.46782
H -3.40510 -2.34757 -2.37206
H -4.78134 -1.60838 -1.49461
H -3.19315 -0.80470 -1.56320
C -1.91085 -4.29649 -3.31789
H -1.57761 -5.11499 -3.98490
H -2.80068 -4.65189 -2.76715
H -2.21246 -3.44466 -3.95283
C 0.13712 -5.47012 -1.43572
H 1.10649 -5.36349 -0.91783
H -0.58373 -5.91167 -0.72390
H 0.27630 -6.19197 -2.26318
C 0.90100 -3.06816 -3.21550
H 1.18034 -3.79973 -3.99775
H 0.57670 -2.14841 -3.74461
H 1.81473 -2.83177 -2.64322
C -1.56347 -0.16370 2.47512
H -2.10576 -0.97557 1.96966
H -2.18920 0.74982 2.51508
C -1.03378 -0.53482 3.87691
H -1.74203 -0.23267 4.66792
H -0.88836 -1.62484 3.95183
C 0.44923 0.91732 2.58987
H 1.47328 0.91669 2.18794
H 0.05339 1.95214 2.61958
C 0.32836 0.22007 3.96514
H 0.35568 0.95308 4.78985
H 1.16420 -0.48333 4.10903

3-Ba
SCF (BP86) Energy = -1638.08160973
Enthalpy 0K = -1637.238754
Enthalpy 298K = -1637.173039
Free Energy 298K = -1637.348476
Lowest Frequency = 6.4743 cm^{-1}
Second Frequency = 10.4679 cm^{-1}
SCF (BP86-D3BJ) Energy = -1638.35239994
SCF (THF) Energy = -1638.12938547
SCF (BS2) Energy = -3067.02711324

Ba -0.47098 -0.01918 -0.13627
Si -1.87637 -3.65062 -1.46445
Si 2.62479 0.23677 -2.49284
Si -2.97938 2.54629 -1.98469
N -1.63846 -2.38123 -0.28511
N 2.22225 -0.08934 -0.84117
N -1.71768 2.16201 -0.84893
C -2.34180 -2.19764 0.90651
C -1.63195 -2.21908 2.16349
C -2.29250 -1.91804 3.37365
H -1.71867 -1.96405 4.30982
C -3.65735 -1.60230 3.42733
C -4.35959 -1.59374 2.20475
H -5.43065 -1.34840 2.20973
C -3.74830 -1.87065 0.97253
C -0.19331 -2.69702 2.21752
H 0.08805 -2.96049 3.25146
H -0.05636 -3.57801 1.56819
H 0.56395 -1.95659 1.88159
C -4.35251 -1.28567 4.73493
H -4.70659 -0.23846 4.77106
H -5.23948 -1.92616 4.89238
H -3.67702 -1.43507 5.59403

C -4.57369 -1.79705 -0.29051
H -4.71196 -2.78848 -0.75801
H -5.57556 -1.38558 -0.08350
H -4.08562 -1.16408 -1.05300
C -2.75022 -3.11596 -3.08843
H -3.80599 -2.83979 -2.93578
H -2.24267 -2.24361 -3.53803
H -2.71979 -3.93379 -3.83147
C -2.80075 -5.16290 -0.74087
H -2.24550 -5.58387 0.11515
H -3.80923 -4.89963 -0.38008
H -2.91040 -5.95927 -1.49813
C -0.16135 -4.27388 -2.05482
H   0.39651 -4.75448 -1.23298
H -0.26846 -5.01126 -2.87077
H   0.45631 -3.44225 -2.43946
C   3.06833 -0.39093 0.20675
C   3.71275 0.62977 1.01007
C   4.56739 0.27167 2.07404
H   5.02516 1.07552 2.66768
C   4.82323 -1.07113 2.43315
C   4.17265 -2.06912 1.67394
H   4.31926 -3.12394 1.94595
C   3.31394 -1.76756 0.59519
C   3.44996 2.09006 0.72220
H   3.69534 2.36635 -0.31685
H   2.38098 2.34318 0.84425
H   4.02700 2.73750 1.40359
C   5.68039 -1.42018 3.63351
H   5.07610 -1.45682 4.55897
H   6.15659 -2.41018 3.52556
H   6.47594 -0.67383 3.80339
C   2.64242 -2.89024 -0.16181
H   2.86741 -2.86824 -1.24192
H   2.94113 -3.87374 0.23864
H   1.54233 -2.82222 -0.10013
C   4.01522 -0.93193 -3.20432
H   3.85834 -1.05392 -4.29080
H   5.02945 -0.48837 -3.11605
H   4.01359 -1.94779 -2.77106
C   1.04229 -0.00771 -3.52341
H   0.62892 -1.02533 -3.41371
H   0.26644 0.72435 -3.22990
H   1.23728 0.15984 -4.59692
C   3.30141 1.97655 -2.94556
H   3.41768 2.06398 -4.04170
H   2.61417 2.77278 -2.61590
H   4.29141 2.18359 -2.49747
C -1.19746 2.91866 0.20197
C -1.61604 2.68154 1.56360
C -0.95981 3.31911 2.63734
H -1.31369 3.11886 3.65815
C   0.10179 4.21875 2.44810
C   0.50150 4.46658 1.12049
H   1.32500 5.17104 0.93546
C -0.11029 3.84975 0.01553
C -2.83385 1.82613 1.85885
H -2.77053 0.77417 1.52218
H -3.02983 1.79453 2.94302
H -3.72758 2.23230 1.35522
C   0.75986 4.92568 3.61499
H   0.35249 5.94463 3.76038
H   0.60386 4.37719 4.55945
H   1.84847 5.03655 3.46520
C   0.37412 4.17446 -1.37792
H -0.27397 4.92077 -1.87171
H   1.39790 4.58659 -1.36047
H   0.34386 3.27674 -2.01917
C -2.77023 4.19798 -2.94041
H -3.63153 4.36443 -3.61273
H -2.71986 5.05859 -2.25048
H -1.85553 4.20246 -3.55705
C -4.73564 2.63686 -1.21674
H -5.00112 1.69199 -0.71257
H -4.80568 3.45008 -0.47277
H -5.49763 2.83289 -1.99317
C -2.99736 1.14046 -3.27905
H -3.84635 1.25850 -3.97530
H -2.07266 1.13455 -3.88257
H -3.10178 0.14804 -2.80500
K   5.92087 -0.81644 -0.50989

3-Ba (anion)

SCF (BP86) Energy = -1609.79766462
Enthalpy 0K = -1608.956749
Enthalpy 298K = -1608.892638
Free Energy 298K = -1609.064457
Lowest Frequency = 8.2590 cm^{-1}
Second Frequency = 12.1207 cm^{-1}
SCF (BP86-D3BJ) Energy = -1610.05773857
SCF (THF) Energy = -1609.85232733
SCF (BS2) Energy = -2467.10180612

Ba 0.11157 0.02894 -0.15814
Si 2.47043 -2.97536 -1.82873
Si 1.99111 3.34353 -1.65767
Si -2.62758 0.03599 -2.76345
N   1.34930 -2.29191 -0.68920
N   1.55346 2.27140 -0.36080
N -2.35158 0.32667 -1.08028
C   0.80571 -2.94094 0.41725
C   1.26440 -2.61710 1.74724
C   0.62827 -3.16506 2.87967
H   1.01347 -2.90263 3.87537
C -0.45296 -4.05600 2.77972
C -0.88014 -4.40383 1.48339
H -1.71568 -5.10914 1.36939
C -0.28808 -3.87664 0.32384
C   2.51086 -1.77334 1.93225
H   2.85965 -1.82293 2.97785
H   3.31879 -2.12496 1.26749
H   2.39034 -0.69716 1.70173
C -1.14407 -4.60466 4.01092
H -2.05643 -4.03100 4.26514
H -1.45654 -5.65493 3.86914
H -0.48279 -4.56695 4.89427
C -0.80178 -4.29066 -1.03499
H -0.06810 -4.91563 -1.57727
H -1.73725 -4.86915 -0.94816
H -0.98264 -3.41060 -1.67710
C   1.75090 -3.39120 -3.56430
H   0.98330 -4.18215 -3.52131
H   1.28408 -2.50044 -4.02004
H   2.55180 -3.73263 -4.24631
C   3.27847 -4.58460 -1.16436
H   3.83015 -4.39181 -0.22797
H   2.51655 -5.35238 -0.94413
H   3.98799 -5.01132 -1.89595
C   3.86802 -1.71436 -2.18145
H   4.45841 -1.51520 -1.27072
H   4.55776 -2.07469 -2.96660
H   3.45174 -0.75008 -2.52371
C   2.02545 2.25670 0.94657
C   1.11488 2.50935 2.03966
C   1.54087 2.37536 3.37693
H   0.82072 2.59009 4.17946

```
C   2.85431  2.01663  3.71825
C   3.75504  1.80011  2.65627
H   4.79398  1.52430  2.88783
C   3.37962  1.90606  1.30791
C  -0.27590  3.04560  1.75792
H  -0.24407  3.78238  0.93816
H  -1.02518  2.28964  1.44582
H  -0.69721  3.52139  2.66026
C   3.28371  1.85403  5.16176
H   3.31466  0.79096  5.47059
H   4.29443  2.26552  5.33679
H   2.58872  2.36906  5.84767
C   4.39658  1.61759  0.22888
H   4.64490  2.51737 -0.36314
H   5.33608  1.23494  0.66342
H   4.01312  0.87412 -0.49219
C   2.91693  4.91476 -1.05742
H   3.13384  5.59346 -1.90195
H   2.30085  5.46872 -0.32796
H   3.87334  4.67263 -0.56363
C   3.03412  2.52890 -3.05226
H   4.02438  2.19774 -2.69844
H   2.51112  1.64097 -3.45176
H   3.18783  3.22725 -3.89571
C   0.40181  3.94462 -2.54287
H   0.63751  4.54133 -3.44325
H  -0.22134  3.09028 -2.86186
H  -0.21424  4.56603 -1.87063
C  -3.27544  0.67968 -0.09033
C  -3.83336 -0.31309  0.79104
C  -4.68644  0.06200  1.84408
H  -5.09552 -0.72700  2.49155
C  -5.03612  1.40105  2.09396
C  -4.48846  2.37350  1.23937
H  -4.73221  3.43212  1.41045
C  -3.63464  2.04976  0.16933
C  -3.52208 -1.77856  0.58036
H  -2.45601 -2.02663  0.73780
H  -4.10360 -2.40502  1.27892
H  -3.75440 -2.09682 -0.44976
C  -5.98552  1.77527  3.21383
H  -7.04563  1.74409  2.89179
H  -5.89274  1.08702  4.07274
H  -5.79325  2.79842  3.58245
C  -3.09024  3.15977 -0.70030
H  -3.60400  3.20681 -1.67747
H  -3.21450  4.14186 -0.21163
H  -2.02184  3.00538 -0.92590
C  -3.41117  1.46107 -3.79086
H  -3.52274  1.16146 -4.84960
H  -4.41496  1.72370 -3.41270
H  -2.78840  2.37107 -3.75830
C  -3.71654 -1.49814 -3.16531
H  -3.26044 -2.42428 -2.77478
H  -4.71901 -1.39974 -2.71209
H  -3.85378 -1.62030 -4.25581
C  -0.89819 -0.29547 -3.52660
H  -0.98934 -0.54911 -4.59782
H  -0.24277  0.59373 -3.46619
H  -0.38719 -1.15023 -3.04426
```

3-Ba (dianion)

SCF (BP86) Energy = -1609.70163399
Enthalpy 0K = -1608.861799
Enthalpy 298K = -1608.798466
Free Energy 298K = -1608.967797
Lowest Frequency = 9.0349 cm^{-1}
Second Frequency = 15.1547 cm^{-1}
SCF (BP86-D3BJ) Energy = -1609.95839726
SCF (THF) Energy = -1609.86743282
SCF (BS2) Energy = -2467.02312957

```
Ba -0.00070 -0.00619  0.21591
Si  3.32856 -0.55976 -1.97001
Si -1.17363  3.14768 -1.98064
Si -2.15157 -2.61372 -1.93746
N   2.46681 -0.97992 -0.52316
N  -0.38453  2.61336 -0.53101
N  -2.09368 -1.63490 -0.50518
C   3.09897 -1.77592  0.44893
C   3.84482 -1.19404  1.53493
C   4.48250 -2.01302  2.48280
H   5.05129 -1.52993  3.29100
C   4.40957 -3.41725  2.44283
C   3.63700 -3.98702  1.41714
H   3.52956 -5.08143  1.37321
C   2.97998 -3.20975  0.44592
C   3.88951  0.30645  1.70899
H   4.61580  0.59279  2.49107
H   4.14299  0.83379  0.77685
H   2.89856  0.69141  2.03331
C   5.07937 -4.27344  3.49847
H   4.47544 -4.34582  4.42554
H   5.23981 -5.30628  3.13856
H   6.06463 -3.86593  3.79394
C   2.11529 -3.89842 -0.58608
H   2.45301 -3.70566 -1.61787
H   2.10560 -4.99132 -0.42251
H   1.07211 -3.53696 -0.53407
C   3.90504 -2.04059 -3.07316
H   4.51021 -2.75998 -2.49323
H   3.04204 -2.58542 -3.49446
H   4.52528 -1.68464 -3.91814
C   4.96677  0.43432 -1.71532
H   4.76797  1.44743 -1.32449
H   5.63406 -0.07713 -0.99918
H   5.51484  0.54234 -2.67103
C   2.21074  0.51530 -3.08720
H   1.76643  1.35824 -2.52950
H   2.79463  0.93568 -3.92715
H   1.38925 -0.08527 -3.51372
C   0.00650  3.54915  0.44260
C  -0.86437  3.91833  1.52883
C  -0.45223  4.86205  2.48552
H  -1.14979  5.12298  3.29508
C   0.81497  5.47168  2.45213
C   1.68567  5.07747  1.42258
H   2.69665  5.51041  1.38179
C   1.32020  4.13593  0.44337
C  -2.20756  3.24426  1.69066
H  -2.79758  3.24746  0.76146
H  -2.08121  2.17988  1.98246
H  -2.79795  3.72804  2.48972
C   1.24460  6.45977  3.51756
H   1.61026  5.95552  4.43486
H   2.06497  7.10864  3.15970
H   0.41057  7.11667  3.82874
C   2.33818  3.72198 -0.59551
H   2.00187  3.93368 -1.62417
H   3.29925  4.24201 -0.43070
H   2.52866  2.63406 -0.55433
C  -0.15862  4.35418 -3.10156
H  -0.76906  4.70777 -3.95468
H   0.17297  5.24325 -2.53602
H   0.73897  3.85890 -3.51141
C  -1.57632  1.63459 -3.07682
```

```
H  -0.65423  1.20348 -3.50246
H  -2.09386  0.84320 -2.50678
H  -2.23141  1.93012 -3.91746
C  -2.83523  4.10514 -1.73606
H  -3.19491  4.52088 -2.69690
H  -3.62626  3.44681 -1.33657
H  -2.70909  4.94559 -1.03073
C  -3.11235 -1.75862  0.45683
C  -3.00793 -2.68370  1.55584
C  -4.05118 -2.80249  2.49042
H  -3.93783 -3.52854  3.30904
C  -5.22044 -2.02321  2.42474
C  -5.30105 -1.07987  1.38677
H  -6.18636 -0.42925  1.32248
C  -4.28364 -0.92374  0.42780
C  -1.74248 -3.48429  1.75887
H  -0.91495 -2.82467  2.09991
H  -1.88130 -4.25432  2.53929
H  -1.39673 -3.97112  0.83457
C  -6.31418 -2.15057  3.46579
H  -7.27995 -1.77006  3.08558
H  -6.46757 -3.20203  3.77397
H  -6.08460 -1.58027  4.38855
C  -4.41883  0.15823 -0.61993
H  -4.40000 -0.24345 -1.64660
H  -5.36065  0.72018 -0.48370
H  -3.58138  0.87700 -0.55780
C  -3.70913 -2.37531 -3.05967
H  -3.71354 -3.10656 -3.89067
H  -4.64144 -2.51593 -2.48426
H  -3.73240 -1.36332 -3.50059
C  -2.12563 -4.52575 -1.65439
H  -1.15613 -4.86023 -1.24578
H  -2.91337 -4.83294 -0.94393
H  -2.29670 -5.06691 -2.60481
C  -0.64573 -2.20991 -3.04347
H  -0.56907 -2.93837 -3.87213
H  -0.74405 -1.20394 -3.48588
H   0.30096 -2.24411 -2.47611
```

4-Sr

SCF (BP86) Energy = -1250.47189814
Enthalpy 0K = -1249.789419
Enthalpy 298K = -1249.736637
Free Energy 298K = -1249.880847
Lowest Frequency = 14.8069 cm^{-1}
Second Frequency = 17.3316 cm^{-1}
SCF (BP86-D3BJ) Energy = -1250.66756975
SCF (THF) Energy = -1250.48434050
SCF (BS2) Energy = -2393.32464151

```
O  -0.03364 -2.50026  0.57020
C   1.14957 -3.30809  0.82310
C  -1.24447 -3.28109  0.77568
H   1.15228 -3.60475  1.89115
H   2.00771 -2.64668  0.61495
H  -2.07742 -2.60037  0.53015
H  -1.29759 -3.57546  1.84293
Si -3.42762  0.20951  1.47544
Si -3.15366 -0.16731 -1.58610
O   0.12333  5.31996 -0.13344
N  -2.49952  0.01320  0.01340
C  -2.24898 -0.02531  2.97356
C  -4.20051  1.95386  1.68085
C  -4.86406 -1.04185  1.69926
C  -1.66382 -0.10168 -2.81848
C  -4.02121 -1.84137 -1.93448
C  -4.37676  1.19615 -2.15195
C   1.29059  4.50318  0.01286
C  -1.04764  4.55645  0.17767
H  -2.79094  0.08124  3.92987
H  -1.44093  0.73006  2.98381
H  -1.78154 -1.02709  2.97400
H  -4.77599  2.03089  2.62081
H  -4.88522  2.19239  0.84880
H  -3.42195  2.73744  1.70669
H  -5.35399 -0.91356  2.68114
H  -4.51170 -2.08616  1.63529
H  -5.63889 -0.90629  0.92439
H  -2.02305 -0.16383 -3.86076
H  -0.96419 -0.95393 -2.70221
H  -1.09194  0.84448 -2.74801
H  -4.37193 -1.90179 -2.98040
H  -4.89704 -1.98278 -1.27846
H  -3.33872 -2.69294 -1.76381
H  -4.67934  1.05172 -3.20461
H  -3.93940  2.20556 -2.06348
H  -5.29498  1.17953 -1.53904
H   1.40284  4.17635  1.07034
H   2.15599  5.13327 -0.25339
H  -1.91247  5.22612  0.03542
H  -1.02435  4.22966  1.24116
Sr  0.00410 -0.00786 -0.05802
Si  3.42549 -0.46274 -1.39737
Si  3.13203  0.41825  1.57269
O  -0.05649 -5.32078  0.15177
N   2.50687 -0.02963  0.01511
C   2.18440 -1.08649 -2.72944
C   4.70410 -1.86954 -1.14128
C   4.38489  0.98669 -2.20801
C   1.61449  0.90977  2.66197
C   4.31952  1.92377  1.58810
C   4.02621 -0.97671  2.53631
C  -1.21561 -4.52221 -0.11274
C   1.12932 -4.54828 -0.06712
H   2.70802 -1.39751 -3.65065
H   1.60945 -1.96483 -2.37854
H   1.46637 -0.30012 -3.03081
H   5.17788 -2.15647 -2.09724
H   5.50977 -1.55692 -0.45427
H   4.24010 -2.77520 -0.71301
H   4.92512  0.65268 -3.11201
H   3.70560  1.80311 -2.51068
H   5.12871  1.41453 -1.51389
H   1.93954  1.24324  3.66317
H   1.03729  1.75212  2.23158
H   0.92708  0.05954  2.84150
H   4.59640  2.20674  2.61948
H   5.25350  1.69551  1.04548
H   3.86872  2.80920  1.10700
H   4.34800 -0.62983  3.53466
H   3.37008 -1.85242  2.68301
H   4.92461 -1.32332  1.99747
H  -1.23679 -4.21610 -1.18258
H  -2.09377 -5.15910  0.08703
H   1.98474 -5.20434  0.16669
H   1.19915 -4.24270 -1.13486
O   0.02530  2.50862 -0.60432
C  -1.16520  3.33574 -0.73112
C   1.22457  3.27875 -0.89702
H  -1.26339  3.65399 -1.78838
H  -2.00962  2.68201 -0.45470
H   2.06682  2.58550 -0.73220
H   1.19234  3.59265 -1.95969
```

4-Sr (anion)

SCF (BP86) Energy = -1250.46460166

Enthalpy 0K = -1249.783697

Enthalpy 298K = -1249.730994

Free Energy 298K = -1249.874149

Lowest Frequency = 14.4217 cm^{-1}

Second Frequency = 21.3040 cm^{-1}

SCF (BP86-D3BJ) Energy = -1250.65773808

SCF (THF) Energy = -1250.51673291

SCF (BS2) Energy = -2393.32853106

O	-0.06765	2.51026	-0.50199
C	1.05494	3.34255	-0.87956
C	-1.29636	3.27919	-0.49157
H	0.91118	3.69011	-1.92298
H	1.94255	2.68991	-0.82667
H	-2.08159	2.56501	-0.18524
H	-1.50121	3.64025	-1.52038
Si	-3.65639	0.00238	-1.43150
Si	-3.19095	-0.04831	1.55227
O	0.23181	-5.37724	0.24768
N	-2.56258	0.05187	-0.06624
C	-2.93196	0.89300	-2.96489
C	-4.04532	-1.78950	-2.01129
C	-5.37406	0.84156	-1.15160
C	-1.78648	-0.37155	2.82091
C	-4.04265	1.55917	2.19267
C	-4.47633	-1.45448	1.86522
C	1.38162	-4.52245	0.14557
C	-0.92053	-4.68539	-0.25812
H	-3.60610	0.76947	-3.83409
H	-1.94492	0.47748	-3.24844
H	-2.80347	1.97653	-2.79052
H	-4.73837	-1.80488	-2.87334
H	-4.49656	-2.39028	-1.20116
H	-3.10748	-2.28400	-2.32603
H	-5.98006	0.78338	-2.07505
H	-5.26224	1.91024	-0.89528
H	-5.95632	0.36469	-0.34355
H	-2.20457	-0.58590	3.82197
H	-1.13024	0.50992	2.92106
H	-1.15508	-1.22849	2.52685
H	-4.36895	1.45066	3.24401
H	-4.92808	1.82239	1.58956
H	-3.34374	2.41415	2.14998
H	-4.81028	-1.46042	2.91970
H	-4.04348	-2.44694	1.64734
H	-5.37372	-1.34182	1.23227
H	1.58657	-4.28369	-0.92015
H	2.23549	-5.09037	0.55563
H	-1.77695	-5.37411	-0.14844
H	-0.78579	-4.44454	-1.33547
Sr	-0.01304	-0.12596	-0.59074
Si	3.13958	0.56072	1.47551
Si	3.65870	-0.38204	-1.34611
O	-0.05237	5.30474	0.08450
N	2.54252	-0.05030	-0.03859
C	1.69533	1.15334	2.59185
C	4.32681	2.07921	1.35159
C	4.08447	-0.72113	2.56289
C	3.02155	-1.77870	-2.49014
C	5.42295	-0.96245	-0.81505
C	3.94614	1.13752	-2.48887
C	-1.15457	4.46327	0.45961
C	1.15907	4.53481	0.07058
H	2.07899	1.65295	3.50069
H	1.03804	1.86884	2.06580
H	1.07191	0.30459	2.92183
H	4.64746	2.41947	2.35402
H	5.23686	1.84399	0.77250
H	3.83457	2.93333	0.85285
H	4.37223	-0.28443	3.53760
H	3.45130	-1.60295	2.76795
H	5.00359	-1.07896	2.06880
H	3.69830	-1.91404	-3.35553
H	2.96392	-2.74441	-1.95554
H	2.01231	-1.55102	-2.88569
H	6.03892	-1.16471	-1.71120
H	5.95779	-0.20976	-0.20947
H	5.37919	-1.89522	-0.22518
H	4.65248	0.90735	-3.30840
H	2.98873	1.44553	-2.94802
H	4.35012	1.99792	-1.92522
H	-1.01726	4.09311	1.49981
H	-2.05831	5.09696	0.42748
H	1.96257	5.21890	-0.25503
H	1.39530	4.17277	1.09521
O	-0.00522	-2.53961	0.45327
C	-1.17019	-3.39531	0.51850
C	1.18073	-3.22430	0.92431
H	-1.38842	-3.62392	1.58241
H	-1.99817	-2.80377	0.09159
H	2.00751	-2.50748	0.76885
H	1.06292	-3.44203	2.00655

S6. Buried volume calculations

Table S9: %V_{bur} values for each amide ligand in **1-AE**, **2-AE** and **3-AE**.

	N(1)	N(2)	N(3)
**1-Mg**	32.5	32.5	31.7
**1-Ca**	29.3	29.0	29.3
**1-Sr**	27.8	27.6	27.3
**2-Mg**	31.9	32.0	32.1
**2-Ca**	29.3	29.7	29.4
**3-Mg**	40.7	29.7	29.0
**3-Ca**	29.1	29.9	28.9
**3-Sr**	27.0	28.0	27.8
**3-Ba·(THF)**	21.6	22.7	21.5

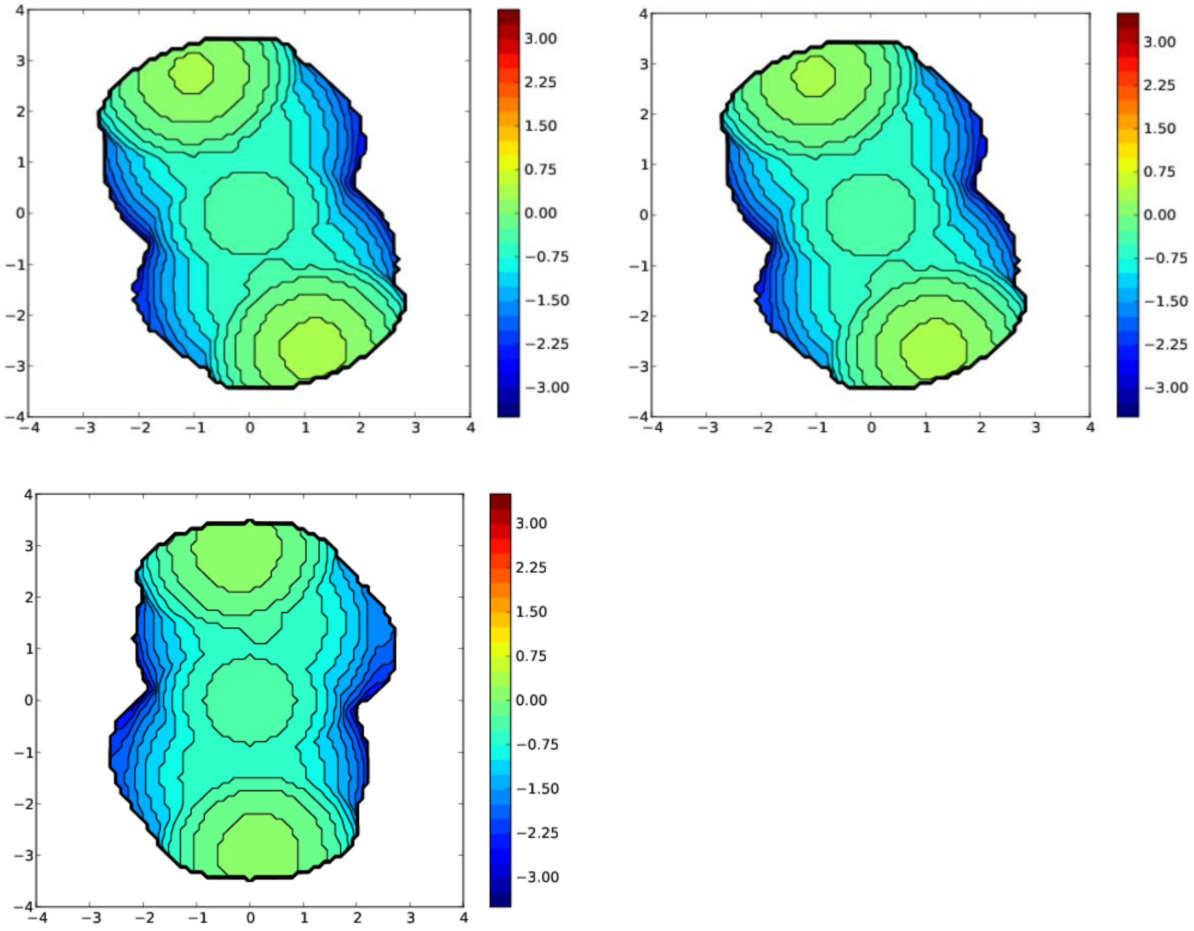

Figure S44: Steric maps of amide ligands in **1-Mg**.

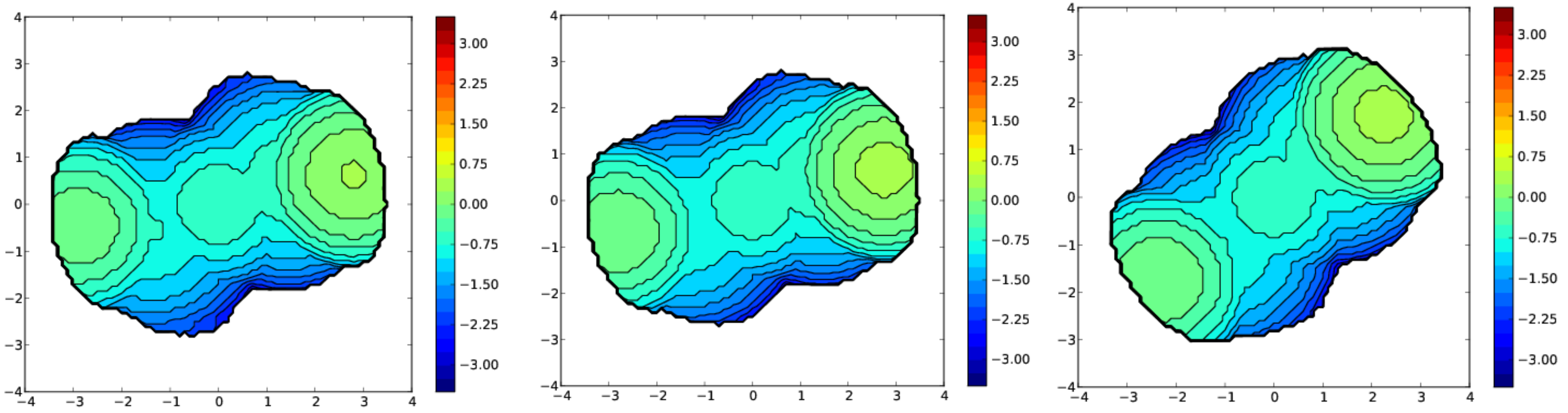

Figure S45: Steric maps of amide ligands in **1-Ca**.

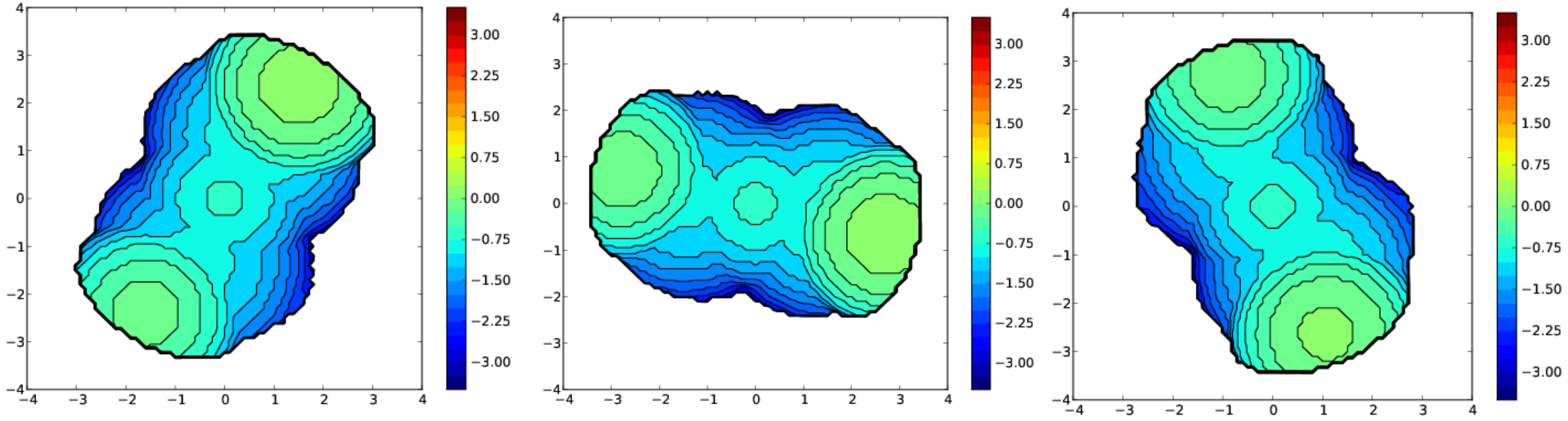

Figure S46: Steric maps of amide ligands in **1-Sr**.

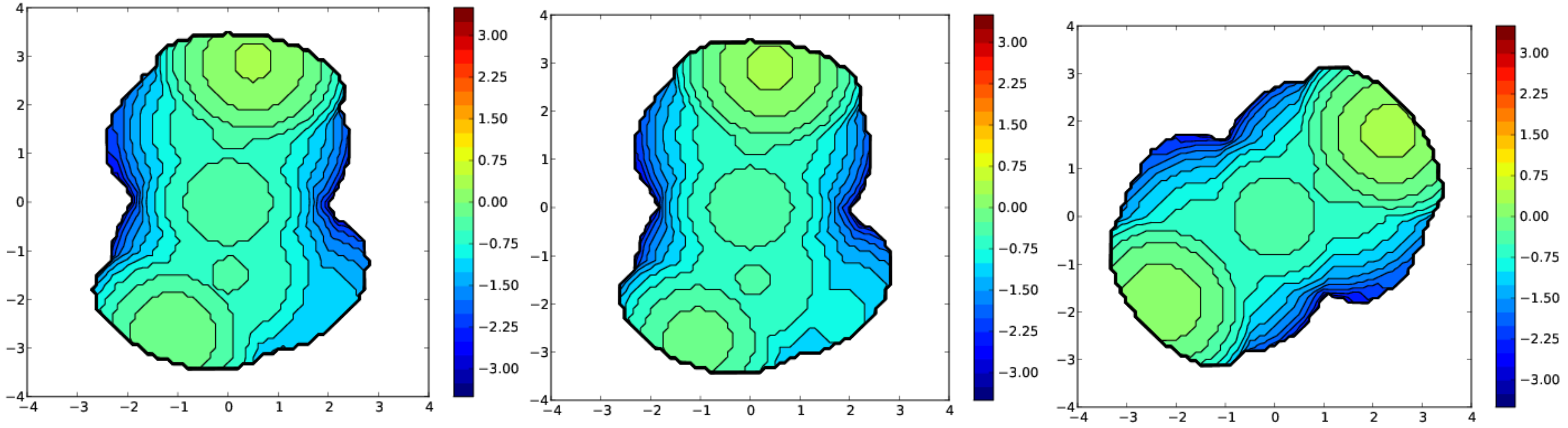

Figure S47: Steric maps of amide ligands in **2-Mg**.

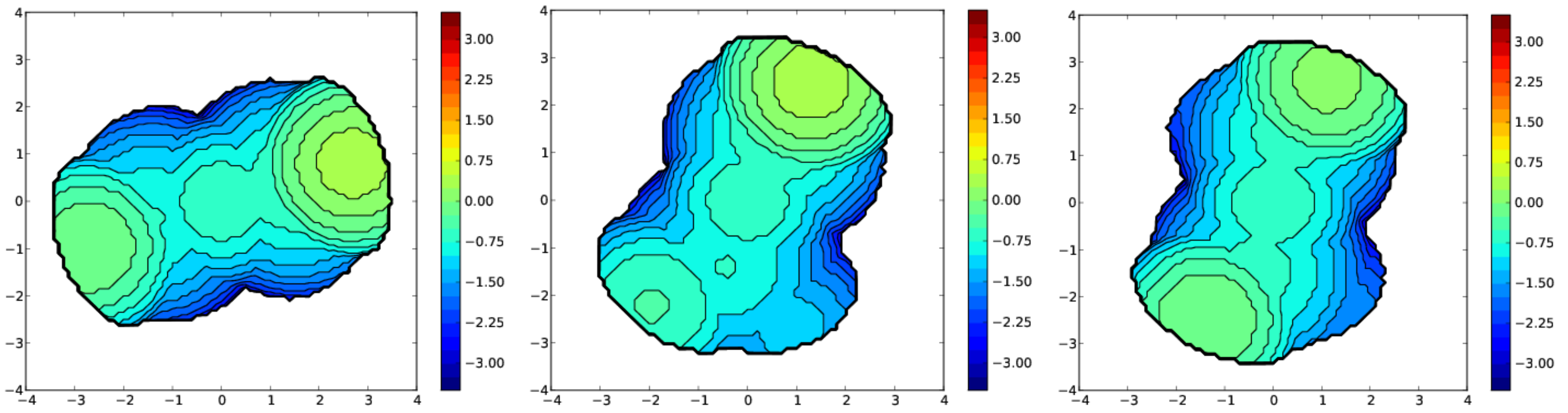

Figure S48: Steric maps of amide ligands in **2-Ca**.

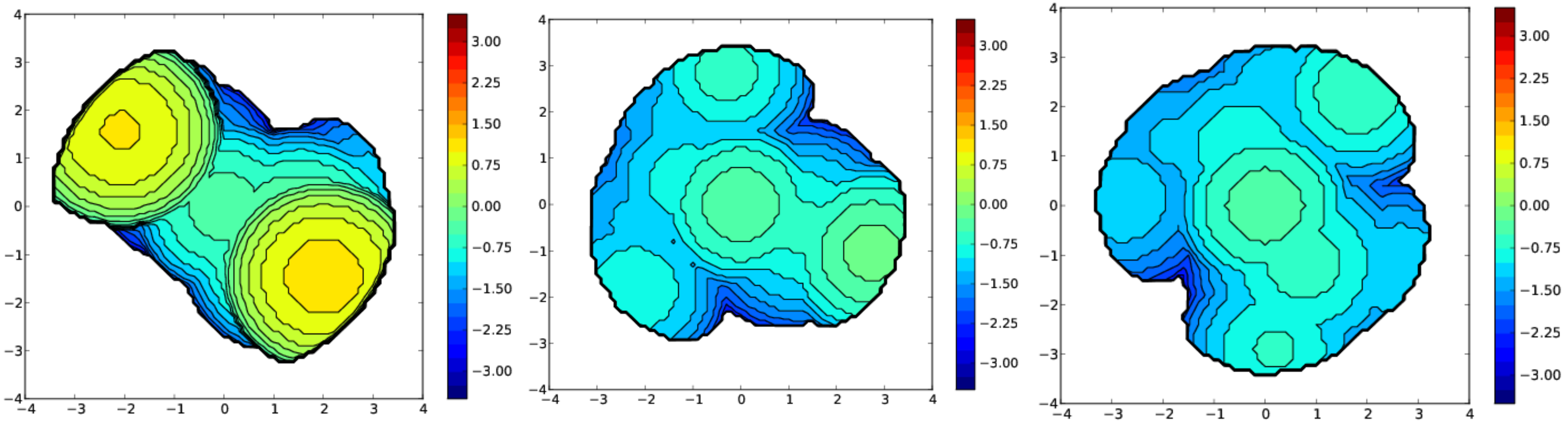

Figure S49: Steric maps of amide ligands in **3-Mg**.

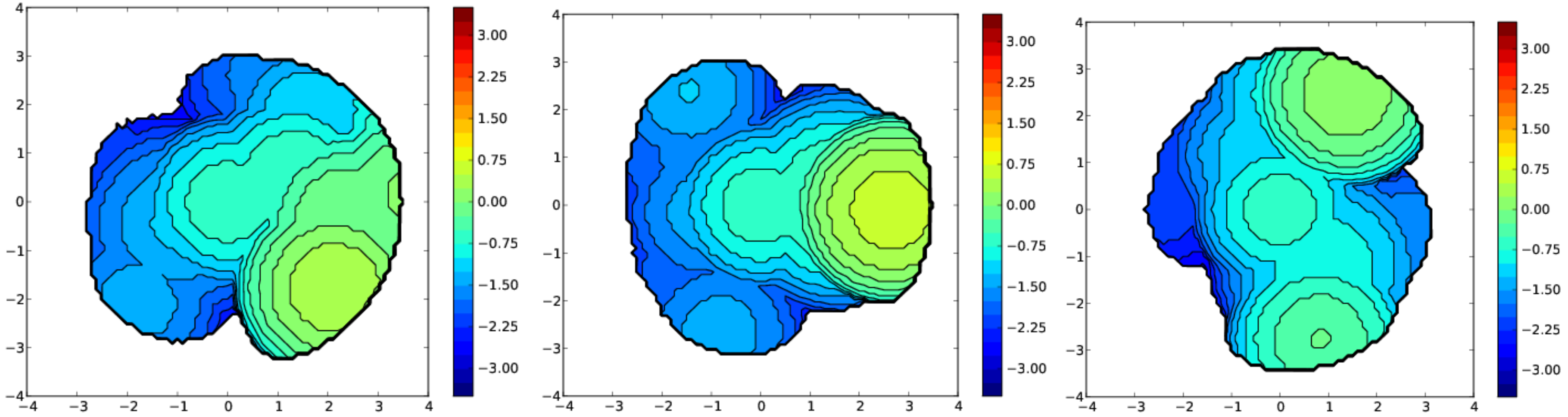

Figure S50: Steric maps of amide ligands in **3-Ca.**

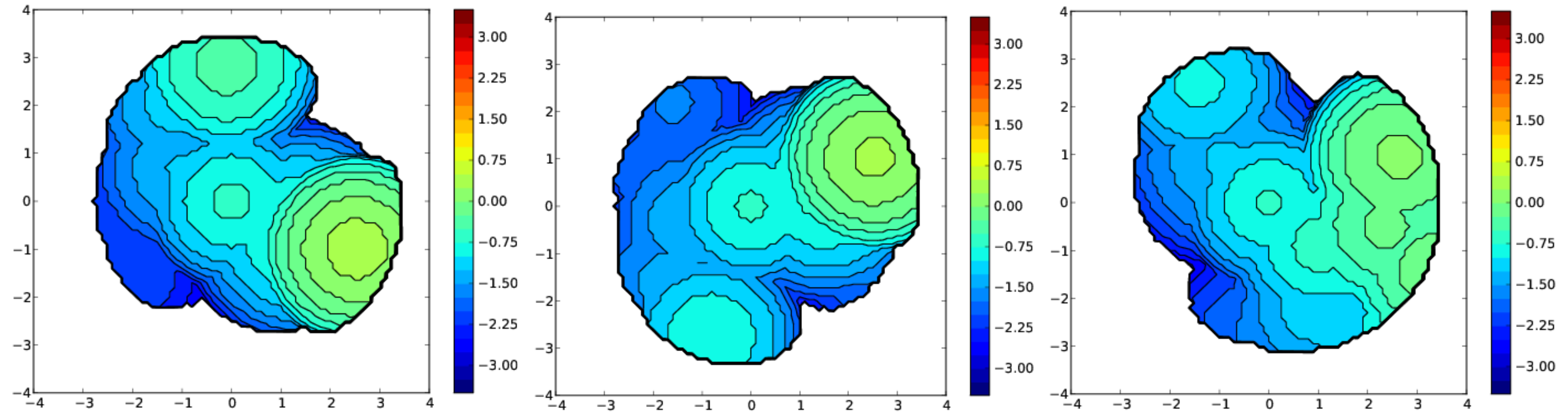

Figure S51: Steric maps of amide ligands in **3-Sr**.

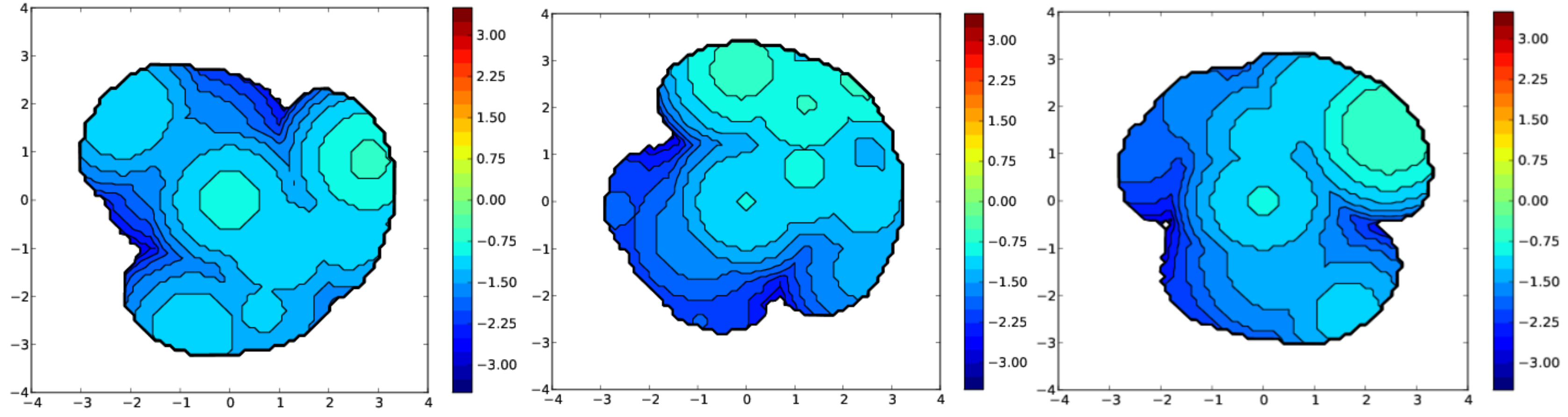

Figure S52: Steric maps of amide ligands in **3-Ba·(THF)**.

S7. References

1. X. He, B. C. Noll, A. Beatty, R. E. Mulvey, K. W. Henderson, *J. Am. Chem. Soc.*, **2004**, *126*, 7444-7445.
2. G.M. Sheldrick, *Program for Area Detector Absorption Correction*, Institute for Inorganic Chemistry, University of Göttingen: Göttingen, Germany, 1996.
3. *CrysAlisPRO*, version 42.80a; Oxford Diffraction / Agilent Technologies UK Ltd / Rigaku: Yarnton, U.K., 2017.
4. G. M. Sheldrick, *Acta Crystallogr. Sect. A* **2008**, *64*, 112-122.
5. G. M. Sheldrick, *Acta Crystallogr. Sect. C* **2015**, *71*, 3-8.
6. O. V. Dolomanov, L. J. Bourhis, R. J. Gildea, J. A. K. Howard, H. Puschmann, *J. Appl. Crystallogr.* **2009**, *42*, 339-341.
7. L. J. Farrugia, *J. Appl. Crystallogr.* **2012**, *45*, 849-854.
8. *POV-Ray*, Persistence of Vision Raytracer Pty. Ltd.: Williamstown, Australia, 2013.
